# Advancements in 3D skin bioprinting: processes, bioinks, applications and sensor integration

**DOI:** 10.1088/2631-7990/ad878c

**Published:** 2024-11-19

**Authors:** I Deniz Derman, Taino Rivera, Laura Garriga Cerda, Yogendra Pratap Singh, Shweta Saini, Hasan Erbil Abaci, Ibrahim T Ozbolat

**Affiliations:** 1 Engineering Science and Mechanics Department, Penn State University, University Park, PA, United States of America; 2 The Huck Institutes of the Life Sciences, Penn State University, University Park, PA, United States of America; 3 Biomedical Engineering Department, Penn State University, University Park, PA, United States of America; 4 Department of Dermatology, Columbia University Irving Medical Center, New York, NY, United States of America; 5 Materials Research Institute, Penn State University, University Park, PA, United States of America; 6 Cancer Institute, Penn State University, University Park, PA, United States of America; 7 Neurosurgery Department, Penn State University, University Park, PA, United States of America; 8 Department of Biomedical Engineering, Columbia University, New York, NY, United States of America; 9 Department of Medical Oncology, Cukurova University, Adana, Turkey

**Keywords:** 3D bioprinting, skin models, skin tissue engineering, biofabrication, biosensors

## Abstract

This comprehensive review explores the multifaceted landscape of skin bioprinting, revolutionizing dermatological research. The applications of skin bioprinting utilizing techniques like extrusion-, droplet-, laser- and light-based methods, with specialized bioinks for skin biofabrication have been critically reviewed along with the intricate aspects of bioprinting hair follicles, sweat glands, and achieving skin pigmentation. Challenges remain with the need for vascularization, safety concerns, and the integration of automated processes for effective clinical translation. The review further investigates the incorporation of biosensor technologies, emphasizing their role in monitoring and enhancing the wound healing process. While highlighting the remarkable progress in the field, critical limitations and concerns are critically examined to provide a balanced perspective. This synthesis aims to guide scientists, engineers, and healthcare providers, fostering a deeper understanding of the current state, challenges, and future directions in skin bioprinting for transformative applications in tissue engineering and regenerative medicine.

## Highlights

Comprehensive review of advanced skin bioprinting techniques, including extrusion, droplet, laser, light-based, and intraoperative methods.
Integration of biosensors with bioprinted skin constructs, enhancing wound healing and real-time monitoring.
Critical assessment of bioinks for skin bioprinting, focusing on the role of collagen, gelatin, decellularized extracellular matrix, chitosan, and fibrin in skin tissue engineering.
Challenges in achieving vascularization and pigmentation in bioprinted skin, with a focus on clinical translation and personalized medicine applications.

## 1. Introduction

Skin, the largest organ in the human body, serves as a dynamic interface between our internal organs and the external environment. Its multifaceted functions encompass protection, thermoregulation, sensation, nutrient storage, and vitamin D synthesis. The complexity of this remarkable organ lies in its complex structure, composed of three primary layers (the epidermis, dermis, and hypodermis) each with distinct roles and contributions to overall skin function [1]. Despite its high regenerative capacity, large-scale deep injuries, (i.e. severe burns) often result in scar repair rather than full regeneration, impacting the recovery of hair follicles (HFs), sweat glands and restoration of skin pigmentation [2]. This limitation significantly affects patients’ prognosis and life quality, leading to difficulties in temperature regulation and potentially life-threatening conditions. Deep burns also affect personal appearance, potentially causing psychological issues, such as depression. While autologous skin transplantation is a common treatment, limitations like donor site scarcity, secondary injuries, and infection risks exist [3, 4]. Conventional grafts, with reduced biological functions and aesthetic mismatch with existing tissues, fall short of fully recapitulating skin function. Tissue engineering offers innovative approaches aiming to develop grafts for skin restoration [5, 6]. Diverse methods, such as foam casting, electrospinning, phase separation, and decellularized matrices, have been used to fabricate skin constructs [7]. Particularly, three-dimensional (3D) bioprinting stands out for its potential impact, enabling the fabrication of scalable tissue analogs with submicron fidelity [4].

Skin bioprinting integrates 3D printing, cell biology, and biomaterials to create functional skin constructs [2]. It involves layer-by-layer deposition of biocompatible ‘bioink’ materials, loaded with biologics, such as but not limited to skin cells and growth factors, to mimic the native skin architecture. The process starts with acquiring patient-derived cells, formulating a bioink, and using a specialized 3D bioprinter to deposit the bioink in a prescribed arrangement. The bioprinted constructs are cultured in a controlled environment, promoting cell viability, differentiation, and tissue remodeling. In addition, 3D bioprinted constructs also hold potential for restoring key skin functions, such as pigmentation. By accurately patterning keratinocytes (KCs) and melanocytes (MCs) within the constructs, bioprinting can facilitate melanin synthesis and transfer, crucial processes for pigmentation restoration [8, 9]. This capability opens new avenues for addressing conditions characterized by pigmentation loss or irregularities, such as vitiligo or post-inflammatory hyperpigmentation. Therefore, skin bioprinting not only offers promise for patients with extensive burns, chronic wounds, or skin disorders but also holds potential for enhancing aesthetic outcomes and improving the quality of life for individuals affected by pigmentation-related concerns. Additionally, skin bioprinting can serve as a valuable tool for pharmaceutical and cosmetic industries enabling the development and testing of new drugs, cosmetics, and skincare products using more realistic and animal-free models [10, 11]. Although skin bioprinting is still in its initial stages of development, considerable progress has already been made. Researchers continue to refine the technology, exploring new bioinks, optimizing 3D culture techniques, and enhancing functional properties of bioprinted skin constructs. With further advancements, skin bioprinting has the potential to reshape the future of wound healing, personalized medicine, and the overall understanding of skin biology [12–15]. Bioprinted skin constructs show great promise for a range of applications, including drug testing, regenerative medicine, and innovative wound care procedures. These constructs are additionally made ‘smart’ by the incorporation of biosensors, which give them the capacity to perceive and react to changes in their environment [16–18]. Recent advances in bioprinting and biosensor technologies have ushered in a new era of personalized medicine and real-time health monitoring, providing creative solutions to aging problems in dermatology, prosthetics, and medical diagnostics [19, 20]. The combination of skin biosensors and bioprinting enables the precise replication of the complex structure and function of the native skin [17–19].

This Review features the state-of-the-art developments in skin bioprinting. It begins with an overview of the skin anatomy and wound healing processes and a comprehensive exploration of bioprinting techniques, including extrusion-, droplet-, laser- and light-based methods, used in skin bioprinting. It delves into the complex aspects of bioprinting HFs, sweat glands, and achieving skin pigmentation. Next, a wide range of bioinks are discussed for their role in successful bioprinting of skin tissue. Further, induced-pluripotent stem cell (iPSC) and vascularization related efforts in the context of skin regeneration are emphasized as crucial elements in constructing functional and native-like skin tissues. Lastly, the review highlights the strategic integration of skin biosensors with bioprinted skin, presenting a symbiotic approach that combines real-time monitoring capabilities with the structural and functional complexity of bioprinted skin. This integration aims to create a synergistic platform, where the precision of skin bioprinting meets the dynamic responsiveness of biosensors, offering unprecedented opportunities for personalized diagnostics, therapeutic monitoring, and improved outcomes in the realm of regenerative medicine and dermatological care.

## 2. Anatomy of skin

**Figure 1.**
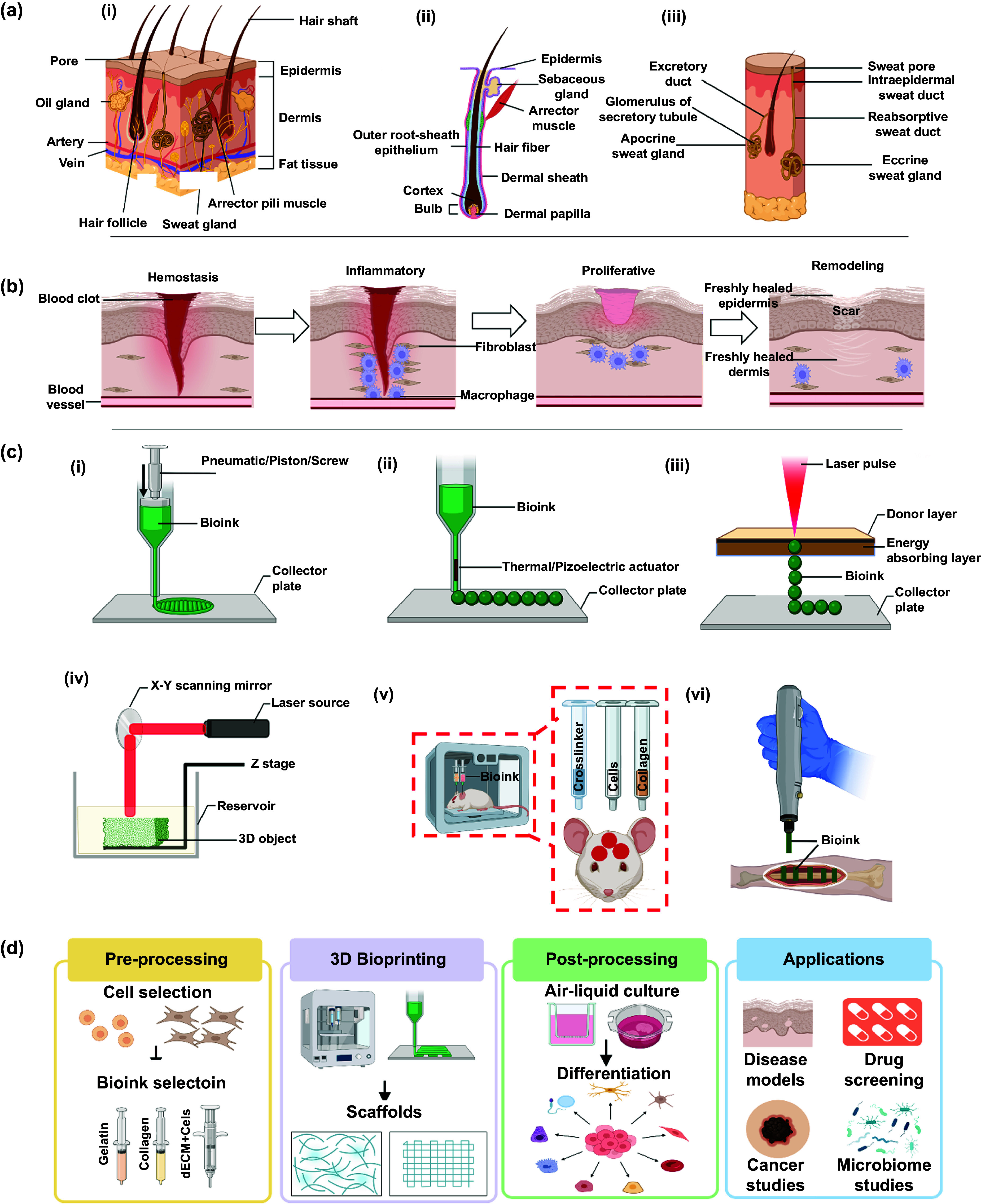
Comprehensive overview of skin anatomy, wound healing, and bioprinting techniques. (a) Anatomy of (i) skin, (ii) hair, and (iii) sweat glands. (b) Steps in wound healing: hemostasis, inflammation, proliferation, and tissue remodeling. (c) Different bioprinting methods: (i) extrusion-based, (ii) droplet-based, (iii) laser-based, (iv) light-based, (v) intraoperative, and (vi) handheld bioprinting. (d) Skin bioprinting unfolds with *in-vitro* cell culture and the selection of bioinks tailored to diverse applications. Precision bioprinting creates customized [BioRender object] constructs designed for wound complementarity. Subsequent post-processing, including stem cell differentiation and maturation, refines the construct. Rigorous evaluation ensures structural and functional integrity before application in various fields, such as disease modeling, drug screening, cancer studies and microbiome studies. This figure was created with BioRender.com.

Skin, the body’s largest organ comprises about 15% of total adult body weight and plays a crucial role in protecting the body against external agents, preventing water loss, and facilitating thermoregulation [21]. It maintains continuity throughout the body’s surface, complemented by the presence of mucous membranes that line various anatomical regions. Structurally, the skin is composed of three layers: the epidermis, dermis, and hypodermis (figure 1(a-i)). Epidermis, the outermost layer of skin, comprises KCs, lacks blood vessels, serves as a dynamic barrier against external factors and undergoes continuous renewal, completing its turnover within 52–75 d [22]. This layer can be divided into five layers from the basal layer to the surface, namely the stratum basale, stratum spinosum, stratum granulosum, stratum lucidum, and stratum corneum. Within these layers, four distinct cell types are present: KCs, MCs, Langerhans cells, and Merkel cells [2]. As cells progress towards the surface, their density decreases, with the stratum corneum primarily comprising flattened, enucleated corneocytes embedded in a lipid-rich matrix [23]. Throughout the epidermis, KCs dominate, followed by MCs, Langerhans cells, and Merkel cells in smaller proportions [24].

Continuously renewing, the epidermis gives rise to derivative structures, such as pilosebaceous apparatuses, nails, and sweat glands [25]. The basal cells within the epidermis undergo cycles of proliferation, ensuring the replenishment of the outer epidermis. Basal cells, serving as the earliest developmental stage of later KCs, include two different proliferative cells: stem cells with an unlimited capacity for self-renewal and transit amplifying cells, which transition from the cell cycle to enter a state between stem cells and cells that eventually differentiate following numerous divisions [26]. Desmosomes connect basal cells to one another and to superficial squamous cells, while hemidesmosomes attach them to the underlying basal membrane, crucial for the integrity and homeostasis of the epidermis [27]. MCs present in the basal layer and HFs contribute to pigmentation [28], with the ratio of MCs to KCs remaining constant throughout life at 1:10 [29]. As a dynamic tissue, the epidermis exhibits constant unsynchronized motion as its cell populations move not only past one another but also past MCs and Langerhans cells while progressing toward the skin surface [30, 31].

The epidermal cell density varies across different skin regions, reflecting differences in turnover rates. For instance, the eyelids have a thinner epidermis compared to the palms and soles. The basal layer harbors the highest density of proliferating KCs, ensuring continuous replenishment of the epidermis. As cells move towards the surface, their density decreases, culminating in the stratum corneum’s lipid-rich, corneocyte-filled structure.

The dermis, positioned between the epidermis and hypodermis, is considerably thicker and is further divided into papillary and reticular dermis. This layer has blood vessels and nerves that supply nutrition and sensation to the skin [32]. Additionally, various appendages such as sweat glands, HFs, and sebaceous glands are present within the dermis (figures 1(a-ii) and (a-iii)).

Moving to the spinous layer, also known as the prickle cell layer, KCs increase in size and establish strong intercellular connections through desmosomes [33, 34]. This layer’s strong interdigitation, characterized by spinous extensions between KCs, contributes to its thorny appearance. Cells close to the basal layer remain mitotically active and are like basal layer cells but are less basophilic, reflecting structural and functional similarities between the two [35]. The predominantly polyhedral-shaped KCs fatten towards the granular layer, where cytoplasm becomes acidophilic [35]. In this layer, two types of bone marrow-derived antigen-presenting dendritic and Langerhans cells, are found, with dendritic processes extending to the stratum corneum [36]. Type I Langerhans cells exhibit a classic dendritic shape with tennis racket-shaped granules, while Type II cells are less dendritic and contain more mitochondria, fewer Birbeck bodies, and a more electron-dense cytoplasm [37].

The granular layer comprises multiple layers of nucleated KCs of polygonal shape without a limiting membrane, containing lamellar membrane-bound lipid granules that synthesize profilaggrin, which, after proteolytic processing, aggregates keratin filaments into dense bundles [38]. High levels of lysosomal enzymes present in the granular layer are required for the elimination of cell components from resilient anuclear corneocytes, the terminally differentiated KCs [39]. The lucid layer is only present in thick skin areas such as the palms and soles, containing non-vital KCs with clear intracellular protein eleidin [40]. Finally, the cornified layer forms the outermost epidermal layer, consisting of flattened anucleated cells filled with keratin and surrounded by a lipid-rich extracellular matrix (ECM) organized into lamellar bilayers, along with enzymes, antimicrobial peptides, and structural proteins [41]. Superficial corneocytes are continuously shed off and replaced by corneocytes from deeper layers, facilitated by desmosomal degradation towards the surface [39].

The dermis plays a crucial role in providing pliability, elasticity, and tensile strength to the skin [42]. It acts as a protective barrier against mechanical injuries, retains water, aids in thermal regulation, and contains sensory receptors for stimuli perception [43]. Moreover, the dermis interacts closely with the epidermis, working in tandem to maintain the characteristics and functions of both tissues. Additionally, they cooperate during the process of repairing and remodeling the skin [44]. Unlike the epidermis, the dermis does not undergo a distinct, parallel sequence of differentiation. However, the structure and organization of its connective tissue components exhibit predictable variations depending on the depth within the skin. The matrix components, such as collagen and elastic connective tissue, also demonstrate depth-dependent differences and undergo turnover and remodeling under normal physiological conditions, pathological processes, and in response to external stimuli [45, 46].

The deepest layer, known as the hypodermis or subcutaneous tissue, is situated beneath the dermis and above the muscle layer. Comprising adipocytes, it plays a significant role in insulation against cold temperatures and acts as a protective buffer against violent trauma. Additionally, the hypodermis provides buoyancy and functions as an energy storage depot. Beyond adipocytes, fibroblasts in the hypodermis play a key role in synthesizing the ECM, secreting various cytokines, and promoting wound repair. Macrophages present in the hypodermis contribute to essential functions, such as phagocytosis, adaptive immunity, and active participation in wound healing [2]. Together, these cellular elements in the hypodermis contribute to various functions of skin, ranging from insulation to immune response and wound healing.

Across the skin layers, cell density varies considerably. The epidermis, accounting for 10–50 cells per square millimeter (mm), is predominantly composed of KCs, constituting approximately 95% of its cellular makeup, with MCs, Langerhans, and Merkel cells comprising the remaining 5%. In the dermis, with a lower density of 2–5 cells per square mm, fibroblasts constitute the primary cell type, alongside immune cells, blood vessels, and skin appendages, collectively representing about 2%–3% of the dermal composition. In the hypodermis, containing fewer cells compared to the epidermis and dermis, adipocytes are the predominant cell type, accounting for around 90% of the cellular population, while blood vessels, nerves, and a minor population of fibroblasts constitute the remaining 10% [42, 47, 48].

## 3. Process of wound healing

Wound healing is a complex process that occurs in three interconnected yet distinct phases: (i) hemostasis and inflammation, (ii) proliferation, and (iii) remodeling, collectively forming the wound-healing cascade (figure 1(b)) [49]. Hemostasis initiates immediately after the skin injury, involving platelet activation and formation of a provisional fibrin matrix within the wound. This triggers the inflammatory phase, marked by the secretion of cytokines, attracting neutrophils and macrophages. Macrophages, critical to wound healing, aid in phagocytosis and produce factors promoting fibroblast proliferation, angiogenesis, and KC migration. During the proliferative phase, fibroblasts migrate to the wound, generating disorganized collagen and myofibroblasts, ultimately leading to wound contraction. Signaling pathways, including angiotensin II and transforming growth factor beta (TGF-*β*), modulate this phase [50]. The subsequent phase, known as the remodeling phase, typically begins around three weeks after the initial injury and extends for over a year. In this phase, all the processes that are activated in the earlier stages are gradually suppressed. This phase replaces granulation tissue with a permanent scar, involving matrix metalloproteinases and collagen production. Each phase works in harmony to restore tissue integrity and maintain homeostasis in wound healing, and any deficiency may impede the process.

The restoration of the barrier function is a crucial aspect of wound healing, as it aims to prevent further damage or infection. This process involves a complex interplay and communication among various cells and mediators right from the initial stages [51]. However, prolonged phases of wound healing or exaggerated responses of the body to the injury can hinder the normal healing process and potentially lead to scarring. Currently, researchers actively study the transition from the inflammatory phase to the proliferative stage of wound repair, considering it a key area of investigation [52]. Additionally, skin epithelial cells are dynamic components that undergo continuous turnover, with old cells being shed from the stratum corneum, through a process known as ‘keratinocyte desquamation’. These cells are replenished by differentiated elements originating from the basal layer, where stem cells undergo proliferation and differentiation. The rate of cell renewal can be influenced by numerous factors, including trauma, hormonal fluctuations, skin conditions, and individual well-being [53]. Notably, the regenerative capacity of the skin following a wound is inversely related to the evolutionary advancement of the species under consideration.

In the context of wounds exceeding a certain size or complexity, the limitations of natural wound healing processes become apparent, particularly when dealing with extensive tissue loss or chronic conditions (such as skin ulcers). Chronic wounds often exhibit a prolonged inflammatory phase, impaired cellular functions, and reduced growth factor availability, contributing to delayed healing and potential complications [54, 55]. In these cases, traditional wound healing mechanisms may not only be insufficient but can also lead to the formation of fibrotic scar tissue, compromising the functional restoration of the affected area [56, 57]. The challenges posed by large wounds necessitate innovative approaches, and bioprinting emerges as an innovative solution. This technology allows for the precise fabrication of customized tissue constructs that closely mimic the native architecture of the affected tissue [58, 59]. In the realm of skin ulcers, which are notorious for their complexity and resistance to conventional treatments, bioprinting holds tremendous promise [60–62]. By depositing bioinks layer by layer, bioprinting facilitates the controlled placement of living cells, growth factors, and biomaterials, creating a microenvironment conducive to optimal cellular behavior. The ability to bioprint complex tissue structures becomes particularly significant in overcoming the challenges associated with natural healing processes in large or chronic wounds. This approach not only addresses the limitations of traditional methods but also opens avenues for enhanced regeneration, offering hope for improved outcomes in cases where natural healing falls short. The precision and customization afforded by bioprinting marks a paradigm shift in the field of wound care, presenting a transformative solution for promoting comprehensive tissue restoration in challenging clinical scenarios.

## 4. B ioprinting of skin

Despite considerable advancements in tissue engineering and regenerative medicine, limitations such as complex reconstruction steps, long culture duration, high costs, low cell viability, and susceptibility to infection obstruct further scientific investigations and clinical applications [63]. Towards this, 3D bioprinting emerges as a promising technique to build highly complex and multicomponent structures, thereby advancing skin tissue engineering for applications not only in wound healing and disease modeling but also in other emerging application areas such as disease modeling, cancer research and microbiome. Skin bioprinting bridges the gap in medical and wound care by enabling the creation of realistic and customized skin substitutes. Bioprinted skin substitutes can precisely match the size, shape, and location of the wound, enhancing healing and lowering the possibility of complications [64, 65].

### 4.1. Need for 3D bioprinting of skin

Skin grafting is a surgical procedure where healthy skin is taken from one part of the body (donor site) and transplanted to another area (recipient site) [66]. There are two main types of skin grafts: split-thickness skin grafts, which involve taking the top two layers of skin, and full-thickness skin grafts, which involve taking all layers of skin from the donor site [67]. Skin grafts are commonly used for various medical reasons, including treating burns, infections, cosmetic reconstruction, and wound healing.

The success of a skin graft depends on factors such as the blood supply at the donor site and the metabolic activity of the skin graft [68]. The integration of graft occurs in phases, including plasmatic imbibition, inosculatory phase, and capillary ingrowth, which establish blood flow to the transplanted tissue [69]. Several techniques are used in skin grafting, including donor site selection, graft sizing and expansion, and graft fixation [70]. Donor sites are chosen based on factors like color match, potential morbidity, and location [71]. Graft sizing involves preformed templates or expansion methods to cover larger areas with smaller sections of skin. Graft fixation is crucial for adherence to the recipient site, and various methods such as staples, dressings, and fibrin glue are used for this purpose.

The process of skin graft healing involves several stages, as described by Medawar in the mid-1940s [72, 73]. Initially, the graft appears white but turns pink over a few days after application to the recipient area, with blanching upon pressure and prompt capillary refill. Collagen replacement begins around the seventh day post-grafting and completes in approximately six weeks [74]. Vascular remodeling in the graft may take months, with host vessel ingrowth forming a characteristic pattern [75]. Lymphatic drainage develops by the fifth- or sixth-day post-grafting, with subsequent weight loss until pre-graft levels are attained by the ninth day. Skin grafts undergo primary contraction due to the recoil of dermal elastic fibers, followed by secondary contraction during healing [76]. Various factors influence graft contraction, including thickness and proportion of dermis. Grafts with more intact dermal collagen tend to inhibit contraction better. Skin grafts also undergo reinnervation, with nerves growing into the grafts from wound margins and the graft bed [77]. The timing and extent of reinnervation vary with graft thickness and recipient site. Skin grafts may undergo pigmentation changes during healing, influenced by factors such as donor site and patient characteristics [78]. Skin substitutes, classified by origin and use, offer alternatives for wound cover and closure [79]. These substitutes aim to mimic natural skin properties, promoting wound healing and tissue regeneration. Successful skin grafting relies on meticulous surgical techniques and avoidance of complications such as hematoma, infection, and fluid accumulation beneath the graft.

Skin substitutes play a vital role in the restoration and regeneration of skin, but they also have limitations. Current substitutes often do not fully mimic the functionality of native skin, mainly due to challenges in vascularization and innervation. Other disadvantages of existing grafts include mechanical fragility, susceptibility to contamination, low engraftment rates and cost [80]. In addition, most substitutes are predominantly acellular or do not contain a wide range of cell types, thereby hindering their ability to fully reproduce the skin characteristics of the native skin [81]. The commercial landscape of skin tissues and skin tissue equivalents, encompassing both acellular and cellular options, serves diverse medical and cosmetic purposes. Acellular substitutes, lacking live cells, provide temporary wound coverage but face limitations such as mechanical fragility and susceptibility to contamination. Cellular substitutes, incorporating live cells, aim for better tissue regeneration yet encounter challenges like limited cell types and inadequate vascularization [82]. These limitations underscore the necessity for innovative approaches like bioprinting. By enabling precise control over tissue architecture and cell distribution, bioprinting offers a promising avenue to overcome these challenges. It holds the potential to create customized skin constructs with enhanced functionality and compatibility, thus advancing the field of regenerative medicine. Investing in bioprinting research and development stands as a strategic pathway to address the shortcomings of current skin tissue products and propel the field forward.

Bioprinting presents a promising avenue for skin tissue engineering, particularly in the realm of cosmetic testing and drug development. Companies like Poietis are leading the way by pioneering 3D bioprinted skin models, which offer a more accurate representation of human skin compared to traditional 2D cell assays or animal testing methods. These 3D bioprinted skin models enable cosmetic companies to comply with regulations banning animal testing while providing essential data for product development. Moreover, the pharmaceutical industry can leverage these advancements to conduct early phase testing of dermatological drugs more effectively, potentially reducing costs and accelerating the time-to-market for new skincare treatments.

With the European Union (EU) Cosmetic Regulation banning animal testing for cosmetic purposes, there is a growing need for skin equivalents as alternatives, given the ethical concerns and the physiological differences between human and animal skin, driving the pharmaceutical and cosmetics industries towards the development of suitable skin models for testing new substances and formulations [83]. The growing demand for skin models in pharmaceutical and cosmetics industries has led to increased interest in 3D skin bioprinting, promising a revolutionary approach to testing new substances and formulations. This technology offers advantages such as faster, cheaper, and more effective assessment of product safety and efficacy. By providing standardized and automated testing platforms, 3D bioprinted skin models offer a more ethical and cost-effective alternative for evaluating pharmaceutical and cosmetic formulations before clinical studies and market launch. Moreover, the ban on animal testing for cosmetic products in the EU has prompted cosmetic companies to seek alternative testing methods. Partnerships with bioprinting companies enable the production of skin models with live cells, meeting regulatory requirements while reducing reliance on animal testing.

Major players like Proctor & Gamble [84], L’Oréal and Poietis [85] have invested over $44 million in research and development of 3D bioprinted skin models, reflecting the significant potential of this field. L’Oreal, in partnership with the University of Oregon, have pioneered a bioprinting method using melt electrowriting to create artificial skin closely resembling natural human skin. This breakthrough offers customizable models for studying skin biology, accelerates the development with a reduced reconstruction time of 18 d, and holds potential for clinical translation, promising new therapeutic options in tissue engineering without animal testing [86]. Similarly, Chanel, in collaboration with LabSkin Creations, has achieved a breakthrough in skincare research by utilizing 3D bioprinting to create reconstructed human skin with pigmentation spots, aiming to better understand the mechanisms behind skin pigment irregularities and enhance the efficacy of skincare products [87, 88]. Brazilian cosmetics giant Grupo Boticário has created engineered skin using bioprinting, revolutionizing cosmetic testing and advancing regenerative medicine [89]. These 3D bioprinted skin models offer more accurate testing conditions and have broader medical applications, such as aiding in burn victim treatment. In a parallel endeavor, CTIBiotech, leveraging CELLINK’s BIO X 3D bioprinter, pioneers the production of full thickness skin models from human cells, providing physiologically- relevant platforms for studying skincare concerns such as sebum production and acne [90]. These breakthroughs in tissue engineering not only diminish reliance on animal testing but also bolster the screening of active ingredients, propelling innovation within the $40 billion cosmetic industry.

A collaborative research project between BASF Care Creations and CTIBiotech has led to the development of the first 3D bioprinted human skin model featuring immune macrophages, enhancing the study of skincare bio-actives [91]. By leveraging CTIBiotech’s advanced bioprinting technology, BASF aims to accelerate the development of innovative skincare ingredients by providing a platform for studying the immune function of macrophages in reconstructed skin models.

JALA Group, a prominent cosmetics company in China, achieved a groundbreaking milestone by successfully printing Asian skin using 3D bioprinting [92]. Collaborating with Labskin Creations, they developed the first Asian skin model through extensive experimentation, aiming to cater to the specific needs of Oriental women and advance cosmetics research for the Asian market. Additionally, other companies such as Rokit Healthcare, are investing in government-funded projects for advancing bioprinting technologies, with a specific focus on human skin tissue. They have successfully developed a diabetic foot ulcer regeneration platform using bioprinting for customized tissue regeneration, aiming to decrease the rate of amputation for patients with diabetic foot ulcers [93]. By utilizing personalized bioink and autologous fat tissue, their innovative solution promotes skin reconstruction, leading to significant wound size reduction and complete closure of ulcers in patients in just weeks, potentially revolutionizing diabetic wound healing. Also, their novel bioprinting technique for treating dermal scarring focuses on tissue regeneration using autologous cells and 3D bioprinting [94]. The method involves collecting autologous stem cells from patients through liposuction, mixing them with a hydrogel to create bioink, and bioprinting uniform dermal patch grafts using the INVIVO 3D Bioprinter, offering potential applications in dermatological wounds, burns, diabetic foot ulcers, and cosmetic procedures.

In the expanding field of 3D bioprinting, the skin bioprinting sector is experiencing significant growth and innovation. The global 3D bioprinting market, valued at $1.20 billion in 2022, is expected to grow at a compound annual growth rate of 20.10% to reach $5.19 billion by 2030 [95]. Spin-offs from universities, such as OxSyBio and CELLINK, are playing a crucial role in advancing skin bioprinting technology through their research and development efforts [96]. These diverse players collectively contribute to the rapid evolution and expansion of the skin bioprinting market.

### 4.2. Bioprinting techniques

Various bioprinting techniques have been explored for developing skin constructs including extrusion-based bioprinting (EBB), droplet-based bioprinting (DBB), laser-based bioprinting (LaBB), light-based bioprinting (LiBB), intraoperative bioprinting (IOB), as described below (table 1).

**Table 1.** Examples of skin constructs fabricated using different 3D bioprinting techniques.

Bioprinting Technique	Bioink	Cells	Study Type	Results	References
Extrusion-based bioprinting	Fibrin-based hydrogel	Human fibroblasts (hFBs), human keratinocytes (hKCs)	*In-vivo* and *In-vitro* Skin constructs were transplanted onto the backs of immunodeficient mice	The study demonstrated successful differentiation of bioprinted human skin, both *in-vitro* and *in-vivo*, with structural similarity to native skin	Cubo *et al* [101]

Extrusion-based bioprinting	Dermal compartment: skin-derived dECM bioink mixed with fibrinogen, hypodermal compartment: a dipose-derived dECM bioink mixed with bovine fibrinogen, additional material: sodium chloride and aprotinin solution	Human dermal fibroblasts (HDFs), Human epidermal keratinocytes (HEKs), Human preadipocytes (HPAs), HUVECs and adipocytes	*In-vitro*	Successfully maturing biofabricated skin constructs using a novel printing platform, the study yielded fully matured 3D skin models with perfusable vascular channels and improved epidermal stratification	Kim *et al* [104]

Droplet-based bioprinting	Collagen type I, thrombin and fibrinogen	Neonatal human dermal fibroblast (NHDF), human dermal microvascular endothelial cells (HMVEC) and neonatal human epidermal keratinocytes (NHEK)	*In-vivo* Athymic nude mice (Foxn1nu/Foxn1nu)	Printed skin grafts accelerate wound healing, promote neovascularization, and closely resemble normal skin tissue, offering a promising alternative to conventional wound dressing such as Apligraf	Yanez *et al* [132]

Droplet-based bioprinting	Collagen type I	Human primary neonatal dermal fbroblasts (hDFs) and human primary neonatal keratinocytes (hEKs)	*In-vivo* 6 week-old male Balb/c nude mice	The human cell-derived skin equivalent fabricated through droplet-based bioprinting/nebulization accurately mimics the morphology of native skin, including the dermis and epidermis, and promotes wound healing more effectively than collagen alone	Lee *et al* [133]
Laser-based bioprinting	Alginate and EDTA blood plasma hydrogel	Human adipose-derived stem cells (hASCs)	*In-vitro*	Laser-based bioprinted 3D grafts of hASCs demonstrated successful adipogenic differentiation, evidenced by lipid accumulation and gene expression of adipogenic markers, with pre-differentiated hASCs printed in grid patterns also showing lipid accumulation	Gruene *et al* [155]

Light-based bioprinting	GelMA/PEGDA hydrogel and cellendes hydrogel	Hs27 human foreskin fibroblasts, HaCaT human keratinocytes, GFP-HUVEC	*In-vitro*	LiBB demonstrates high cell viability, tissue complexity and offering a promising avenue for producing physiologically relevant full-thickness skin constructs with precise architectural features and marker expression patterns	Hafa *et al* [174]

Intraoperative bioprinting	Fibrinogen with/without adipose-derived ECM (adECM), thrombin + factor XIII (Fxiii)	Adipose-derived stem cells (ADSCs)	*In-vitro* and *in-vivo* 10–12 weeks old female RNU nude rats	The study utilized IOB to effectively reconstruct full-thickness skin defects in immunedeficient rats, achieving accelerated wound closure and enhanced adipogenesis, vascularization, and formation of hair follicle-like structures	Kang *et al* [119]

Handheld bioprinting	Alginate-collagen and fibrin based (thrombin and fibrinogen)	Human dermal fibroblasts and keratinocytes.	*In-vitro* and *in-vivo* murine and porcine experiments	The handheld bioprinter enabled precise *in-situ* deposition of biomaterial and tissue sheets onto surfaces or wounds, showcasing varied sheet morphologies and maintaining cell viability	Hakimi *et al* [190]

#### 4.2.1. EBB.

EBB stands out as a vital bioprinting technique for producing replacement tissues and organs. This is primarily due to its cost-effectiveness and ease of control during the bioprinting process [97]. This method employs extrusion forces for the controlled deposition of bioinks (containing living cells) onto a substrate (figure 1(c-i)). EBB finds extensive use in biomedical applications, particularly in tissue engineering. This method has been widely utilized due to its versatility in depositing multiple bioinks within a single construct, with a single print head or by employing multiple print-heads. Researchers leverage this feature to generate constructs with spatial variations in bioinks, cell types and densities, and signaling molecules [98]. Moreover, extrusion-based bioprinters allow for operating at higher cell densities compared to other bioprinting techniques [99]. However, there are certain limitations associated with EBB. The flow of cells through the nozzle and the stress they experience while in the nozzle prior to deposition can lead to decreased cell viability and function, as cells are exposed to shear stress [100]. Among various bioprinting methods, EBB stands out as the most prevalent and readily accessible technique due to its shallow learning curve.

**Figure 2.**
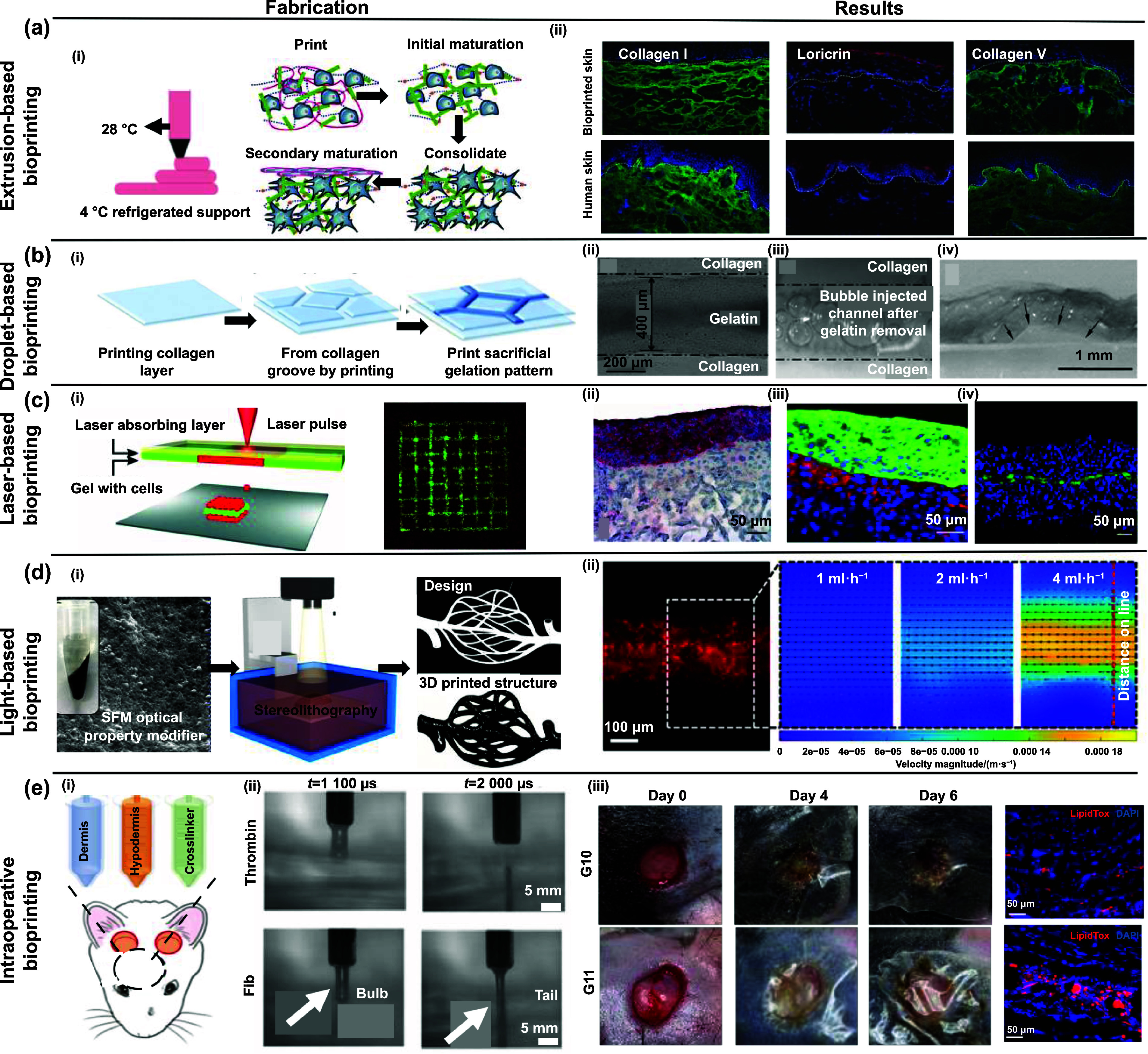
Overview of different 3D bioprinting techniques and their applications. (a) Extrusion-based bioprinting technique. (i) Schematic depiction of the 3D bioprinting process, followed by consolidation and maturation stages utilizing a custom-made bioink, (ii) comparison of epidermal differentiation and dermal marker profiles between bioprinted skin and normal human skin from a healthy donor. [10] John Wiley & Sons. © 2016 WILEY-VCH Verlag GmbH & Co. KGaA, Weinheim. (b) Droplet-based bioprinting technique. (i) Illustration outlining the step-by-step process for constructing a multi-layered collagen scaffold measuring 10 × 10 mm. The procedure involves the utilization of a 3D bioprinter for embedding and subsequent removal of sacrificial gelatin patterns, (ii) gelatin line pattern printing observed between the designated dotted lines within the collagen groove, (iii) the gelatin line pattern, once printed, was embedded within a multi-layered collagen scaffold, and selectively eliminated. The introduction of air bubbles facilitated inspections to assess the targeted removal of gelatin under stereomicroscopy, (iv) cross-sectional images of the hydrogel scaffold containing channels, obtained after 1 week of incubation. Arrows highlight the margins of the channels in the cross-section. [116] John Wiley & Sons. Copyright © 2009 Wiley Periodicals, Inc. (c) Laser-based bioprinting technique. (i) Schematic of the LBB illustrating how the cell-hydrogel compound is propelled forward in a jet through the pressure created by a laser-induced vapor bubble. An overhead view of a printed grid structure showcases the micropatterning capabilities of LBB, highlighting fibroblasts (green) and keratinocytes (red), (ii) hematoxylin and eosin (H&E) staining provides a tissue-like pattern revealing all bioprinted cells, (iii) immunoperoxidase staining specifically highlights cytokeratin 14 in reddish-brown, emphasizing the bi-layered structure of keratinocytes. All cell nuclei are counterstained in light blue with hematoxylin, (iv) a cross-sectional view of the bioprinted structure, captured immediately after bioprinting, displays transduced fibroblasts (red) and keratinocytes (green). [117] John Wiley & Sons. Copyright © 2012 Wiley Periodicals, Inc. (d) Light-based bioprinting technique. (i) Scanning electron microscope (SEM) images showcasing melanin nanoparticles, schematic depiction of the 3D projection stereolithography process, design representation of a complex blood vessel structure, and a photograph capturing the hydrogel structure 3D-printed using digital beam patterns, (ii) visualization of velocity magnitude fields correlated with the external injection rate within an artificial blood vessel model. Reprinted with permission from [118]. Copyright (2018) American Chemical Society. (e) Intraoperative bioprinting technique. (i) Overview of IOB utilizing a DBB method for the reconstruction of hypodermis and dermis compartments in a surgical context. IOB was implemented on nude rats, each with three 6-mm full-thickness skin defects on the crania, (ii) refinement of the jetting process for bioink solutions involves the ejection of solutions from a micro-valve device, causing them to break into streams of multiple droplets upon exiting the nozzle orifice, (iii) display of representative wound images and LipidTox staining images at Day 28, providing a visual assessment of the healing progress following the IOB procedure for full-thickness skin reconstruction. Reproduced with permission from [119]. © 2023 The Authors. Publishing services by Elsevier B.V. on behalf of KeAi Communications Co. Ltd CC BY-NC-ND 4.0.

In the context of skin bioprinting, the studies conducted by Cubo *et al*, Rimman *et al*, Pourchet *et al*, Kim *et al* and Admane *et al* represent significant advancements [10, 101–104]. Cubo *et al* focused on precise layer-by-layer deposition of bioinks containing human skin cells, resulting in constructs that closely mimic natural skin attributes [101]. The bioprinted constructs exhibited meticulous cell organization, structural integrity resembling intact skin tissue, and the presence of vital skin-specific markers. One year later, Rimman *et al* devised a holistic bioprinting strategy for generating standardized 3D bioprinted skin models with human primary cells, addressing the need for reproducibility in substance testing and regenerative medicine. Their approach integrated a bioprinter, a light-induced bioink polymerization unit, user-friendly software, and standardized procedures, enabling the creation of complex skin tissue structures. Despite challenges in achieving a fully-stratified epidermis, their method supported long-term cell viability, proliferation, and ECM production, showcasing its potential. In parallel, Pourchet *et al* bioprinted a specialized bioink, containing cells and a supportive matrix construct human skin tissue substitutes without the need for an additional supporting scaffold (figure 2(a)) [10]. The bioprinted skin substitutes displayed attributes resembling natural skin, including precise cell arrangement, structural integrity, and expression of skin-specific markers, demonstrating functional proliferation, differentiation, and synthesis of essential ECM components. These studies collectively underscore the potential of advanced bioprinting techniques for skin regeneration. Kim *et al* developed a novel method for the fabrication of perfusable/vascularized (P/V) full-thickness skin models [104]. Their approach involved the use of a polycaprolactone (PCL)-based transwell combined with a sacrificial gelatin hydrogel to ensure structural integrity. Sequential extrusion of the PCL mesh and collagen-rich adipose-fibrinogen bioink formed the hypodermal compartment, while a gelatin-based vascular bioink, crosslinked with thrombin, created vascular channels. This method enabled the precise placement of human umbilical vein endothelial cells (HUVECs) within channels, promoting vascularization. Additionally, the platform allowed for the provision of different mediums for tissue maturation, including endothelial growth medium through the vascular channels. In another study, Admane *et al* demonstrate the fabrication of 3D bioprinted skin constructs that closely mimic the native human skin in structure, mechanics, and biochemistry [103]. These constructs replicate the undulated dermal-epidermal junction and exhibit molecular similarities to human skin, including key pathways related to skin development and physiology. Through enzymatic crosslinking of the silk-gelatin bioink, they maintain structural stability over time. Compared to traditional collagen-based equivalents, they offer enhanced stability and morphology, which are promising in applications, such as cosmetics and drug testing.

The viability of cells in EBB processes is influenced by various factors such as shear stress, dispensing pressure, nozzle geometry, printing speed, and deposition velocity [105, 106]. First, shear stress, a result of the force applied to expel the bioink through the nozzle, poses a significant risk to cell viability, which it also varies based on factors like nozzle diameter and viscosity [107]. Dispensing pressure affects cell viability parabolically, with higher pressures leading to increased shear stress and decreased cell viability [107, 108]. Nozzle geometry, particularly conical needles, influences shear stress distribution, affecting cell damage rates [109]. Printing speed correlates with extrusion pressure impacting cell viability inversely, where rapid movements subject cells to heightened stress [110]. Additionally, temperature fluctuations within the printing environment play a pivotal role [111, 112]. Optimizing these parameters ensures the maintenance of high cell viability that is essential for the functionality and success of bioprinted tissue constructs.

In the context of skin bioprinting, EBB stands out as a promising technique due to its versatility and cost-effectiveness [98, 113]. This method allows for the use of various biomaterials, facilitating the creation of complex skin structures with the potential to mimic the native skin tissue. Despite its advantages, challenges such as potential cell damage during extrusion and lower resolution (considering the highly thin nature of skin layers) compared to alternative methods need to be addressed [114]. Ongoing research in skin bioprinting aims to refine the precision and resolution of EBB, positioning it as a valuable tool for developing complex and functional skin constructs in regenerative medicine. Also, EBB has been widely employed either independently or in conjunction with DBB, showcasing its versatility and applicability in skin bioprinting [115]. The continuous exploration and refinement of such techniques signify ongoing efforts to advance skin tissue engineering, paving the way for innovative solutions in regenerative medicine and personalized skin therapeutics.

#### 4.2.2. DBB.

DBB is a technique that involves the physical manipulation of a bioink solution to generate droplets (figure 1(c-ii)). It utilizes the forces of gravity, atmospheric pressure, and fluid mechanics to propel droplets onto a receiving substrate [120]. There are two primary categories of DBB: continuous inkjet and drop-on-demand bioprinting. Among these, drop-on-demand bioprinting has gained broader applications due to its unique advantages as it is particularly well-suited for the controlled delivery of biomaterials. Drop-on-demand bioprinting involves the controlled release of the bioink from a nozzle at a specific pressure [121, 122]. These droplets then pass through charging plates, where they acquire an electric charge. By passing through deflection plates, the placement of the droplets is controlled by adjusting the strength of the electric field [123, 124]. In continuous inkjet bioprinters, droplets are continuously produced regardless of whether they are needed or not. To minimize material waste, such bioprinters typically include a droplet recovery device. However, the recovery process may introduce contamination, which is undesirable in bioprinting [125]. Moreover, the uncontrolled deposition nature of the process makes it unsuitable for bioprinting purposes. Drop-on-demand inkjet bioprinting is the preferred method due to its several advantages. Compared to continuous inkjet bioprinters, drop-on-demand bioprinters offer more economical and precise control over the bioprinting process, making them ideal for patterning of biologics. These bioprinters typically consist of one or multiple printheads, each containing a fluid chamber. The bioink stored in the fluid chamber is held in place by surface tension at the nozzle orifice [126]. Pressure pulses are then applied to the fluid chamber using thermal, piezoelectric, or electrostatic actuators. This causes a droplet to be expelled when the bioink overcomes the surface tension. The ability to generate droplets on demand allows for enhanced efficiency and accuracy in bioprinting applications [125]. Drop-on-demand bioprinters include two primary types: thermal inkjet (TIJ) and piezoelectric inkjet (PIJ) bioprinting. TIJ utilizes thermal energy to create vapor bubbles within the print chamber, causing the rapid expansion of these bubbles to expel tiny droplets of bioink from the nozzle onto the substrate [127]. This method offers high resolution and precision due to the controlled ejection of small droplets and is cost-effective and widely available. However, the heating process can potentially affect cell viability due to high temperatures involved, limiting its use. In contrast, PIJ uses a piezoelectric actuator to induce mechanical deformation of the print chamber, generating pressure waves that eject droplets from the nozzle [128]. This method avoids thermal stresses, preserving cell viability and accommodating a broader range of bioinks, including those sensitive to temperature changes. Nevertheless, the mechanical stress from the piezoelectric actuator can still affect cell viability, and the technology is more complex and expensive compared to TIJ. TIJ offers high precision and is cost effective, but it is limited by potential thermal damage and bioink compatibility [129]. PIJ preserves cell viability better and is versatile with different bioinks, but it is more expensive and can still introduce mechanical stress to cells. Each method’s unique advantages make them suitable for different bioprinting applications, depending on specific requirements such as cell type, bioink properties, and the desired resolution and precision of bioprinted structures.

The collective efforts of researchers, exemplified by Lee *et al* and their progressive studies, have significantly advanced the field of skin tissue engineering through DBB. The transition from conventional 2D cell cultures to multi-layered 3D approaches has opened new avenues for investigating skin biology and exploring novel therapeutic strategies [130]. Building on this foundation, subsequent studies by Lee *et al* (figure 2(b)), Skardal *et al*, Lee *et al*, Yanez *et al*, Kim *et al* and the recent work by Lee *et al* [133] showcase the evolving landscape of DBB technologies [116, 131–135]. In recent research, Lee *et al* explored DBB for fabricating human skin equivalents (hSKE), showcasing its distinct advantages over EBB [133]. Their study successfully replicated native human and mouse skin morphology, demonstrating precise mimicking of the keratinization process in the epidermis while confirming that DBB/nebulization maintains high cell viability comparable to conventional culture conditions. In a wound healing model, hSKE outperformed collagen, exhibiting superior wound healing with enhanced collagen deposition, regular dermis regeneration, and improved vascularization. The introduction of multi-layered hydrogel scaffolds, incorporation of fluidic channels, and the application of amniotic fluid-derived stem cells illustrate the versatility of DBB in enhancing structural integrity and promoting therapeutic effects in large skin wounds. Furthermore, creation of bioprinted human skin tissue with layered organization, including the epidermis and dermis, and the development of bilayer skin grafts with functional microvasculature emphasize the importance of mimicking native skin attributes for successful skin reconstruction [2].

The printing process in DBB exerts significant influences on cell viability. Mechanical impacts during printing, such as cell clogging at the print head and damage from falling droplets, pose challenges to maintaining cell integrity [136, 137]. Optimizing printing parameters becomes crucial to mitigate these risks. Additionally, preventing cell aggregation through gentle stirring and biosurfactant use is vital for maintaining printing quality and cell viability [138, 139]. Accurate cell positioning and managing shear stress inside the nozzle further impact cell viability and bioprinting resolution [140]. Moreover, non-contact bioprinting methods, like acoustic droplet bioprinting, introduce additional considerations such as acoustic radiation force and wavelength effects on cell viability. In this regard, Ng *et al* found that higher cell concentration slows droplet impact, improving bioprinting accuracy and cell survival [141]. At the same time, droplet evaporation creates a hypertonic environment, impacting viability negatively. Optimal parameters for high viability (>90%) include using a 4 million cells/mL bioink, 20 nl droplets, and limiting print time to 2 min per layer. Also, low air pressure (<5 psi) facilitates high cell viability, especially with low viscosity bioinks like collagen solutions for skin bioprinting [141, 142].

In the content of skin bioprinting, DBB offers distinct advantages by providing precise deposition of small droplets containing cells and bioinks in a non-contact manner [125]. This precision enables the creation of complex, finely detailed structures that closely resemble the complex architecture of skin tissue [143]. The gentle droplet ejection process contributes to high cell viability, minimizing potential damage during bioprinting [144]. Additionally, the capability of DBB to produce multilayered structures aligns well with the need for accurately replicating the stratified nature of skin [145]. However, challenges include the limited range of printable biomaterials, potentially restricting the variety of components that can be used in the bioprinting process [125]. Additionally, concerns about nozzle clogging may arise, particularly when a cell density over 1 million ml^−1^ is utilized affecting the reliability of the bioprinting process. Ongoing research focuses on optimizing DBB techniques for skin applications, aiming to leverage their precision and cell-friendly characteristics while addressing limitations to advance the field of skin tissue engineering. Overall, DBB signifies a persistent commitment to advancing skin tissue engineering, with a focus on precision, functionality, and therapeutic efficacy.

#### 4.2.3. LaBB.

LaBB is a technique that employs a laser as the energy source to deposit bioink onto a substrate (figure 1(c-iii)) [146]. This process involves the utilization of a pulsed laser to propel the bioink from liquid donor-ribbon to a receiver-slide metal film [147]. LaBB begins with laser energy deposition, where a pulsed laser beam deposits energy onto the absorbing layer, causing rapid evaporation and plasma formation that generates a vapor bubble at the bioink-support interface [148]. The bubble’s growth and collapse create mechanical stress that propels the bioink towards the substrate, with bubble dynamics being crucial for influencing the printing regime and cell stress. Jet formation occurs next, where a jet propels the bioink towards the substrate, and the jet’s stability and velocity are key for precise deposition.

Several critical parameters must be controlled for controlled printability in LaBB. Laser parameters, such as wavelength (UV or near-IR), energy per pulse (typically 1–20 *µ*J), repetition rate, and beam focus affect the precision and speed of bioprinting. Bioink properties, including viscosity, surface tension, and layer thickness, influence jet formation, stability, and droplet size, with higher viscosity reducing splashing and improving print resolution. Substrate characteristics, such as wettability and coating, affect droplet spread and print resolution, and proper surface treatment can enhance cell adhesion and viability. Environmental conditions like humidity and temperature also impact bioink viscosity and structural stability. LaBB exhibits exceptional precision in positioning of cells, as evidenced by several studies highlighting the capability of placing a single cell per droplet [149, 150]. Despite its high precision, it is important to note that LaBB can be expensive to perform and faces challenges in terms of stability and scalability [151]. Despite these limitations, LaBB has demonstrated significant potential when combined with other biofabrication techniques. By integrating LaBB with complementary approaches, its advantages can grow apace, thereby expanding its applications in the field of bioprinting [152, 153].

In 2010, Koch *et al* introduced the LaBB technique for human skin construction and stem cell therapy, establishing its reliability for precise cell placement [154]. The laser printing process, specifically utilizing laser-induced forward transfer (LIFT), has demonstrated significant influences on cell viability, particularly in the context of bioprinting skin cells and human mesenchymal stem cells (hMSC). Firstly, the survival rate of cells post-transfer was notably high, with skin cell lines exhibiting a survival rate of (98 ± 1) % and hMSC showing a survival rate of (90 ± 10) % compared to the control. This indicates that the LIFT process itself did not induce significant cell death. Furthermore, assessments of cell proliferation revealed that the transferred cells maintained their ability to proliferate effectively. Fibroblasts, KCs, and hMSC showed no major differences in their proliferation rates compared to the control over a period of 7 d. This suggests that the bioprinting process did not adversely affect the cells’ proliferative capacity. In a series of studies, LaBB demonstrated its potential in tissue engineering. Gruene *et al* employed LaBB to create 3D tissue grafts using human adipose-derived stem cells (ADSCs), showcasing the technique’s adaptability without adverse effects on cell viability or behavior [155]. Koch *et al* focused on skin tissue generation, highlighting LaBB’s precision in arranging fibroblasts and KCs to mimic dermis and epidermis, respectively, preserving cell viability, and enabling tissue-specific functions (figure 2(c)) [117]. The study by Micheal *et al* utilized LaBB for a fully-cellularized skin substitute, achieving successful bioprinting and showcasing its promise in creating complex tissues for applications like burn therapy [156]. These findings collectively underscore LaBB’s potential for *in-vitro* modeling and engineering functional living grafts with applications extending to organ bioprinting in the future.

LaBB shows minimal detrimental effects on cell viability. Studies report near 100% post-bioprinting viability across various cell types [157, 158]. The process does not increase DNA strand breaks or apoptosis, nor does it affect proliferation or differentiation potential. Minimal expression of heat shock proteins indicates low cellular stress [159, 160]. Overall, LaBB maintains cell viability and functional, supporting its use in biomedical applications. Related to this topic, Zhang *et al* explored how laser energy influences droplet formation and printing dynamics in a cell-laden bioink [161]. Increasing laser fluence boosts jet velocity and alters droplet morphology. Living cells raise transfer thresholds and change jet size. Non-ideal jetting behaviors occur due to material properties and cell interactions. LaBB phase diagrams reveal bioink printability, influenced by viscoelastic properties and cell content. Compared to DBB, LaBB results in higher jet velocities and different mechanisms for non-ideal jet formation [162]. Understanding these dynamics is crucial for skin bioprinting applications.

In the context of skin bioprinting, LaBB’s advantages lie in its high precision, enabling the creation of complex skin structures with fine details, along with key advantages such as being nozzle-free method, which eliminates nozzle-clogging and contamination risks, offers the capability to a bioprint high-density cell suspensions and bioinks with a wide viscosity range (1–300 mPa·s^−1^), and facilities high cell viability and resolution down to single-cell printing [163, 164]. However, challenges such as restricted range of printable materials, inability to bioprint thick structures, potential thermal damage and the cost and complexity of laser-based bioprinters exist along with limited scalability of bioprinted constructs [165–168]. In addition, bioprinting of heterocellular stratified skin tissues necessitates the switching of the ribbon in a manual way, which is tedious and needs further development for automation purposes.

#### 4.2.4. LiBB.

LiBB, a 3D printing technique that utilizes light to selectively solidify bioinks containing living cells, holds promise in tissue engineering (figure 2(c-iv)). The method’s high resolution allows for precise and complex 3D structure reconstruction, and the selective exposure to light ensures accurate control over the bioprinting process. LiBB, including techniques like stereolithography (SLA), digital light processing (DLP), and volumetric bioprinting (VBP), uses concentrated UV or alternative light sources for polymerization of the bioink according to a computer design [169]. SLA offers flexibility for applications like insulin delivery, while DLP provides high-resolution, rapid curing for tissue regeneration. VBP constructs structures rapidly within seconds with visible light, demonstrating high biocompatibility. Advantages include precision and versatility but concerns over UV light and extended fabrication times in layer-wise approaches are challenges that require ongoing refinement [170]. It can be combined with other bioprinting methods to create complex tissues for regenerative medicine and drug testing [171–173]. However, challenges include the potential for cell damage due to prolonged light exposure and the limited selection of biomaterials responsive to photopolymerization. Achieving scalability for larger tissues and organs poses additional hurdles. Ongoing research seeks to enhance the overall effectiveness of LiBB methods.

In a recent study, Hafa *et al* presented LUMINATE (light sheet-based ultrafast microscopic non-contact and 3D enhanced bioprinting), an integrated light sheet bioprinting system for creating viable full-thickness skin constructs, emphasizing speed, resolution, and real-time characterization [174]. Simultaneously, Shin *et al* introduced an innovative approach by incorporating melanin nanoparticle-infused silk fibroin hydrogels (SFM) into transparent poly (ethylene glycol)-tetraacrylate (PEG4A) bioink for LiBB (figure 2(d)) [118]. This integration significantly improved bioprinting resolution by minimizing light scattering, enabling precise construction of complex structures like blood vessels. The resulting SFM/PEG4A hydrogel exhibited enhanced mechanical strength and biocompatibility. This advancement underscores the potential for high-resolution LiBB, offering a pathway for creating complex biological structures with superior cellular viability and proliferation.

LiBB, including methods such as SLA, DLP or VBP presents certain influences on cell viability during bioprinting. Firstly, direct exposure of cells to UV light used for photopolymerization can induce cytotoxic effects and genetic damage [175]. However, recent advancements, such as visible light-based photoinitiator systems or less toxic photoinitiators, aim to mitigate these effects and enhance cell viability [176]. Several photoinitiators have been highlighted for their effectiveness in this regard. Irgacure 2959, with a maximum absorption efficiency at 275 nm, is a commonly used photoinitiator known for its water solubility and good biocompatibility [177]. LAP (Lithium phenyl-2,4,6-trimethylbenzoylphosphinate), absorbing at 375 nm, is a visible light photoinitiator offering rapid polymerization and minimal cytotoxicity, making it ideal for bioprinting applications involving living cells. Eosin Y, which absorbs at 514 nm, is often used in combination with co-initiators like triethanolamine and N-vinylpyrrolidone and is suitable for creating intricate structures with high cell viability. VA-086, with an absorption peak at 385 nm, is known for its ability to initiate polymerization under mild conditions, thus maintaining cell viability during bioprinting. These photoinitiators are critical in ensuring the success of the bioprinting process by facilitating the polymerization of bioresins while maintaining cell viability and structural integrity [178]. The cytotoxicity of photoinitiators should be minimal to ensure high cell viability, with hydrophilic groups in photoinitiators reducing cellular uptake and cytotoxic effects. Additionally, photoinitiators with higher molar extinction coefficients are preferred as they allow better control over the cure depth and print resolution. Longer wavelengths are generally less harmful to cells, making visible-light photoinitiators preferable for bioprinting applications involving living cells. Additionally, the crosslinking method employed in LiBB can impact cell viability. For instance, using DLP to encapsulate cells may lead to cytotoxicity, especially if magnetic markers are introduced to facilitate spheroid formation [179]. However, strategies to minimize cytotoxicity, such as using fewer toxic markers or optimizing the crosslinking process, can improve cell viability.

In the context of skin bioprinting, the advantages of LiBB include precision in reproducing skin layers, and customization for diverse patient needs. Challenges encompass limited biomaterial options, depth penetration issues, possible toxicity exposure due to the use of photoinitiators and scalability concerns. Despite challenges, ongoing research positions LiBB as transformative in skin tissue engineering. Advancements showcase the potential to overcome limitations, offering precise and customizable solutions for the future of skin bioprinting.

#### 4.2.5. IOB.

IOB, also known as ‘*in situ*’ bioprinting, refers to the direct bioprinting of bioinks at a defect site under surgical settings (figure 1(b-v)) [180]. This technique is particularly beneficial for reconstructing curved surfaces and sophisticated geometries, which are challenging for conventional bioprinting approaches that typically work on flat substrates. However, before IOB can be implemented in clinical scenarios, thorough testing is required to ensure its safety and aseptic nature. Despite this, the field has made significant advancements in recent years, with numerous studies highlighting its potential, even for the repair of skin-involved composite tissues [131]. Unlike traditional methods, IOB ensures strong and precise reconstruction directly onto live subjects, minimizing the risk of graft disintegration and contamination. It addresses the complex anatomical variations of skin tissues, allowing for rapid, on-site regeneration without the need for *in-vitro* culture or extensive modifications. IOB’s real-time design capability promotes personalized and efficient skin tissue repair, making it a game changer in regenerative surgical care [181].

The skepticism expressed by Campbell and Weiss regarding the feasibility of IOB has been met with significant advancements in bioprinting strategies, robotics and bioinks [182]. These developments have paved the way for the application of this technology in pre-clinical settings, presenting potential avenues for improved patient outcomes. However, as IOB is still in its early stages, it is imperative to acknowledge and address the challenges associated with its clinical implementation. Further exploration is crucial to refine the technology, enhance intraoperative delivery protocols, optimize biomaterials, and fine-tune cell selection, to create clinically and economically viable systems. Studies conducted by Binder *et al*, Albanna *et al*, Wang *et al*, and Albouy *et al* underscore the transformative potential of IOB in skin reconstruction [183–186]. These investigations have demonstrated precise placement of bioinks containing skin cells onto burn wounds, accelerated wound healing with autologous skin cells, multi-tissue layering for defect closure, and successful regeneration of dermis and epidermis layers using a robotic arm. The use of a pharmaceutical-grade bioink loaded with dermal fibroblasts has shown significant enhancements in wound healing, reduction of scar tissue formation, and improved vascularization [187–189]. In a recent study, Kang *et al* delved into the realm of IOB for craniomaxillofacial (CMF) skin reconstruction (figure 2(e)) [119]. Utilizing a DBB bioprinter during surgeries, IOB crafts personalized 3D structures with human-derived bioink. This real-time adaptable method accelerates healing, achieving near-complete wound closure within two weeks while enhancing tissue integration. In CMF reconstruction, IOB emerges as an innovative and efficient approach, providing dynamic and responsive solutions compared to traditional methods. This collective body of research signifies a paradigm shift, transforming initial skepticism into tangible and promising results, thereby offering precision and patient-specific solutions for revolutionizing wound care.

Moreover, the introduction of handheld bioprinters (figure 1(c-iv)), exemplified by the work of Hakimi *et al*, represents a notable advancement in IOB technologies [190]. These handheld devices offer direct control over bioink deposition, eliminating the need for medical imaging and reducing operational complexities associated with the use of bioprinting. This innovation not only streamlines the intraoperative process but also enhances the potential for widespread clinical adoption. In summary, the evolving landscape of IOB holds great promise for meeting clinical demands and ushering in a new era of precision and patient-specific solutions in wound care and tissue regeneration [191, 192].

### 4.3. Bioinks for skin bioprinting

The selection of an appropriate bioink is crucial as it directly influences the functionality and success of bioprinted tissues and organs [193]. When choosing a bioink, several key factors should be considered, including mechanical, rheological, and biological properties that align with target tissues. These properties ensure that the bioink possesses the necessary characteristics for proper tissue formation [193]. Furthermore, it is important for bioinks to support cellular growth and proliferation without causing any detrimental effects on cell phenotype [194]. The printability of bioinks for skin bioprinting is crucial and influenced by various biophysical and biochemical attributes such as shear-thinning, gelation kinetics, recoverability, biodegradation, and biocompatibility [195, 196]. The viscosity of bioinks should be low enough to protect cells from shear stress and prevent clogging, yet the bioinks should solidify rapidly to maintain shape. Hydrogel-based bioinks are commonly used for skin regeneration and wound healing, with key features including extrudability, filament classification, shape fidelity, and bioprinting accuracy [197, 198]. For skin bioprinting, a suitable bioink involves the combination of suitable biomaterials and cells, such as KCs, MCs, and fibroblasts with relevant ECM components [199]. Various hydrogels are commonly used as bioink materials due to their unique properties. They do not only serve as a structural material but also function as a delivery system for bioactive molecules [200]. An ideal bioink should fulfill three essential conditions: (i) exhibit biocompatibility to promote high cell viability, (ii) possess appropriate rheological behavior to enable successful bioprinting, and (iii) demonstrate rapid crosslinking ability to retain the desired 3D structure after bioprinting [201]. The commonly used bioinks for skin bioprinting are discussed in the following sub-sections.

#### 4.3.1. Collagen.

Collagen is a common bioink due to its presence as a structural protein in human tissues and its various beneficial properties. It serves as a main component of the skin ECM, providing structural integrity and physical support to the skin [202, 203]. Its appeal for skin tissue engineering is further enhanced by its low immunogenicity [204]. Additionally, it is biodegradable, biocompatible, and readily accessible, making it suitable for use in tissue engineering applications. Its porous structure and permeability allow for the exchange of nutrients and waste products when used as a scaffold [205]. Collagen-based scaffolds also impact the functions of skin cells, influencing cell shape, differentiation, migration, and the synthesis of skin ECM proteins to enhance skin regeneration [206, 207]. Consequently, collagen is a popular choice for skin bioprinting.

However, the inherent weaknesses of collagen necessitate supplementary considerations for skin bioprinting. Despite its benefits, collagen alone may fall short in terms of mechanical strength, shape constancy, and microstructure needed for optimal cell attachment and proliferation at 37 °C [208]. In addition, there are difficulties with direct bioprinting of collagen, particularly when loaded with cells or spheroids. Difficulties include the risk of cell damage or reduced viability during bioprinting due to shear forces and pressure, as well as the need to optimize bioprinting parameters to maintain the delicate balance between collagen viscosity and crosslinking. Achieving precise spatial control of cell placement within the collagen matrix is also challenging, and ensuring proper fusion and integration of bioprinted layers to form functional skin requires careful consideration of biological and mechanical factors [209, 210]. Chemical crosslinking is not widely used in studies to increase collagen’s printability due to concerns related to biocompatibility, potential alterations to mechanical properties and cell responsiveness, and challenges in mimicking natural crosslinking mechanisms [211]. Instead, methods include combining collagen with other substances like chitosan, fibrillar collagen, fibrinogen, and thrombin, as well as carefully regulating cell suspensions and densities [212, 213].

The studies by Nocera *et al* and Osidak *et al* underscore the versatility of collagen in bioprinting applications. Nocera *et al* demonstrated the successful interaction of cells with bioprinted collagen constructs, affirming their biocompatibility by supporting cell attachment, proliferation, and metabolic activity [208]. Osidak *et al* addressed collagen’s limitations by optimizing concentrations and bioprinting conditions, resulting in enhanced mechanical properties of collagen constructs suitable for various tissues [214]. The shear-thinning behavior and precise temperature control demonstrated in these studies showcase collagen’s adaptability for complex bioprinting processes. Furthermore, the incorporation of spheroids within collagen constructs, opens avenues for creating high cell density structures with diverse morphological and biomechanical properties [214]. Overall, collagen, even without crosslinking agents, provides a conducive environment for cellular activities, making it a valuable bioink for bioprinting applications.

#### 4.3.2. Gelatin.

Gelatin is a biodegradable polymer obtained through the partial hydrolysis of collagen [215]. Unlike its precursor collagen, gelatin is a water-soluble natural polymer with superior water absorption capabilities. The behavior of gelatin solution depends on factors like temperature, pH, concentration, and preparation methods, showcasing its ability to form hydrogels through a sol-gel transition process at low temperatures [216, 217]. These gelatin-based hydrogels exhibit a unique thermo-reversible character, enabling their bioprinting [218]. Gelatin exhibits high biocompatibility and degradability, which is advantageous for tissue regeneration as it allows for the replacement of the bioink with newly synthesized skin over time. In addition, gelatin has appropriate rheological properties, making it suitable for EBB [201]. However, gelatin alone often lacks sufficient mechanical strength and viscosity for bioprinting applications [201]. It is rarely utilized as a standalone bioink due to the inherent complexities associated with fine-tuning its temperature and viscosity for bioprinting purposes, as well as its inability to undergo a reversible reaction during bioprinting [219, 220]. To overcome these limitations, gelatin is frequently combined with other biomaterials, such as alginate. The combination of gelatin and alginate creates a synergistic effect, where gelatins high elasticity complements alginate’s high viscosity. This synergistic combination enhances the printability and shape fidelity of the resultant bioink [221]. Researchers have explored various strategies to optimize gelatin-based bioinks for skin tissue engineering. In this regard, GelMA has emerged as a versatile and promising bioink for wound healing applications. Its unique properties, such as heightened temperature sensitivity and light-induced crosslinking, contribute to its adaptability in creating precise and mechanically-stable structures for natural bioinks. For example, Xu *et al* developed a dual-crosslinked hydrogel combining GelMA and silk fibroin methacrylate, called ‘SilMA’, to accelerate wound healing [222]. This innovative hydrogel exhibits superior mechanical strength, controlled biodegradation, and enhanced tissue regeneration *in vivo*, showcasing GelMA’s vital role alongside SilMA for effective wound healing.

Ng *et al* explored polyelectrolyte gelatin-chitosan (PGC) hydrogels, focusing on viscosity as a critical factor for bioprinting [223]. This study identified an ideal viscosity range and higher concentrations of gelatin in PGC hydrogels exhibiting enhanced suitability for bioprinting, accompanied by favorable biocompatibility for cell attachment and proliferation. Piola *et al* further optimized gelatin and xanthan gum combinations, showcasing the optimum bioprintability and resolution. Crosslinking with glutaraldehyde demonstrated biodegradability, aligning with the gradual absorption needed for wound healing. Biocompatibility assessments confirmed cell proliferation within bioprinted structures, underlining the suitability of these gelatin-based bioinks for tissue engineering applications [224]. The natural properties of gelatin, coupled with its versatility in bioprinting, position it as a favorable choice for skin tissue engineering. Innovative approaches, incorporating natural extracts and combining gelatin with other biomaterials, have further optimized gelatin-based bioinks for enhanced printability, mechanical strength, and bioactive properties. These advancements hold significant promise in developing effective solutions for restoring skin integrity.

#### 4.3.3. Skin decellularized ECM (dECM).

The ECM constitutes the acellular component of tissues and organs, creating a microenvironment crucial for specific cellular functions. Each tissue possesses a meticulously structured ECM composed of numerous components and proteins that preserve the native tissue architecture. Notably, the skin dECM can be derived through proper protocols and repurposed as a scaffold for tissue regeneration [225, 226]. Moreover, the utilization of skin dECM, encapsulating endothelial progenitor cells (EPCs) and ADSCs, has been demonstrated to promote neovascularization, re-epithelialization, and wound closure *in vivo* [227, 228]. The dECM plays an indispensable role in supporting cell survival, proliferation, and offers the essential physical and mechanical microenvironment for cells [229–231]. Numerous research groups have utilized dECM-based bioinks, with the expectation that these bioinks would enhance cell proliferation and differentiation by providing essential biomolecules, including growth factors [232, 233].

In a recent study, Kang *et al* utilized IOB with human adipose-derived ECM (adECM) and ADSCs for CMF skin defect reconstruction in rats, achieving successful skin repair within two weeks, highlighting the potential of adECM and IOB for stable tissue-engineered skin for CMF reconstruction [119]. In a related context, studies conducted by Kim *et al* and Jang *et al* exemplify the remarkable potential of dECM-based bioinks in skin tissue engineering [229, 234]. These bioinks demonstrated superior stability, minimal shrinkage, and enhanced functionality in creating organized epidermis differentiation. Moreover, *in-vivo* experiments showcased accelerated wound closure, re-epithelialization, and neovascularization, emphasizing the therapeutic benefits of dECM [229]. The integration of growth factors and cytokines in these bioinks contributed to their positive effects, underscoring their potential as advanced solutions for skin tissue engineering [234]. As these studies pave the way for further research, including clinical validation, the use of dECM-based bioinks holds promising prospects for developing effective skin substitutes in burns and autologous skin transplantation.

#### 4.3.4. Chitosan.

Chitosan, derived through the deacetylation process of natural chitin, is a linear polysaccharide [235]. Being a natural biomaterial, chitosan has garnered considerable attention for its applications in wound dressing and skin tissue engineering. Notably, chitosan exhibits robust antibacterial properties, further enhancing its appeal as a biomaterial for artificial skin production [236, 237]. Chitosan’s unique properties, including its linear polysaccharide structure and robust antibacterial characteristics, underscore its potential for artificial skin production. The studies conducted by Hafezi *et al*, Zhu *et al* and Ullah *et al* exemplify its applications in innovative bioink formulations for skin regeneration, demonstrating impressive cell viability and survival rates. For example, Hafezi *et al* reported a bioink tailored for skin regeneration. Comprising crosslinked chitosan and genipin, the bioink boasts optimal rheological properties, ensuring suitability for EBB. The bioink, housing KCs and fibroblasts, demonstrated over 88% cell viability in 7 d [238]. Zhu *et al* utilized a bioink containing guanidinylated/PEGylated chitosan (GPCS), collagen, and gelatin for bioprinting of a hSKE with multi-layered KCs. GPCS demonstrated excellent biocompatibility, enhancing fibroblast proliferation and interaction between KCs. The engineered skin showed improved intercellular junctions and epidermal stratification compared to conventional collagen-based scaffolds, making it a promising model for biomedical research [239]. Ullah *et al* reported a bioink, polyethylene oxide-co-chitosan-co-poly(methylmethacrylic-acid), for bioprinting, emphasizing the importance of chitosan in enhancing structural stability and DNA interactions. The bioink demonstrated favorable properties, making it a promising candidate for skin tissue engineering applications [198].

#### 4.3.5. Fibrin.

Fibrin assumes a crucial role in fostering cell proliferation, differentiation, and vascularization [240]. Its gelation is a spontaneous process resulting from interactions between fibrinogen and thrombin [241, 242]. The incorporation of cells within fibrin offers several advantages, such as uniform dispersion and robust adhesion to specific sites within bioprinted constructs, attributable to the abundance of cell adhesive sites [243, 244]. Fibrin-derived bioinks demonstrate favorable characteristics for DBB, such as excellent printability, accurate maintenance of shape, and compatibility with living tissues in the realm of 3D bioprinting [245]. These attributes present promising opportunities for ongoing advancements and diverse applications in the field.

Studies conducted by Hoppenbrouwers *et al* and Bacakova *et al* underscore the efficacy of fibrin in improving skin wound healing [246, 247]. In diabetic rats, a fibrin matrix accelerated wound closure and enhanced perfusion without inducing an immune response. Fibrin-coated electrospun nanofibers, as investigated by Bacakova *et al*, demonstrated stability and significantly enhanced human dermal fibroblast activities, suggesting promise for skin tissue engineering applications. Horch *et al* utilized fibrin sealant as a biomatrix for transplanting autologous KCs, showcasing effective reepithelialization and addressing challenges associated with standard sheet grafts [248]. Cavallo *et al* created a bioink by blending fibrinogen and alginate for bioprinting of skin equivalents demonstrated excellent printability and *in-vitro* biocompatibility, emphasizing its potential for wound healing applications [249]. In a study by Mazlyzam *et al*, a bilayer hSKE (B-FF/FK SE) was developed using fibrin as a scaffold [250]. This clinically compliant skin graft composite, exhibiting properties like native human skin, holds promise for wound healing applications and showcases its relevance for future research and pharmaceutical testing. Collectively, these studies highlight the versatility and clinical potential of fibrin-based constructs in advancing the field of tissue engineering and regenerative medicine.

#### 4.3.6. Hyaluronic acid (HA).

HA holds great promise in tissue engineering due to its natural compatibility, cytocompatibility, and modifiable properties through chemical adjustments [251]. Derived from the ECM, HA offers inherent biocompatibility, supporting cell adhesion and tissue regeneration [252]. Its cytocompatibility facilitates diverse applications across tissue types. Additionally, its tunability through chemical modification allows precise adjustment of mechanical properties for bioprinting purposes, enhancing versatility [251]. However, challenges arise due to the potential for rapid degradation, demanding careful formulation for stability. Despite these obstacles, HA bioinks open a compelling avenue for regenerative medicine.

In a stride towards efficient skin regeneration, Si *et al* introduced a 3D bioprinted HA-based wound dressing with sustained drug release [253]. The crosslinked hydrogel exhibited controlled rheological properties, high swelling, and extended degradation. Incorporating Nafcillin with minimal cytotoxicity, the hydrogel demonstrated a controlled drug release profile. Bioprinted constructs maintained cell viability, presenting a versatile solution for customizable wound dressing applications. Building on this innovation, Bavaresco *et al* delved into bioprinted collagen and HA scaffolds crosslinked with dehydrothermal treatment (DHT) for skin tissue engineering [254]. DHT emerged as a crucial factor in increasing crosslinking, improving resistance to collagenase, and enhancing mechanical properties. The addition of HA influenced pore diameter, with DHT effectively reversing this effect. Notably, collagen scaffolds exhibited no cytotoxicity, while those with HA displayed cytotoxicity for Vero cells. These findings underscore the potential of DHT-treated bioprinted collagen and HA scaffolds, offering tunable properties for skin tissue engineering. Expanding the horizons of wound care, Zhou *et al* employed a catechol-HA bioink system, creating elastic cellular constructs with inner channels that mimic vascular structures [255]. Thrombin-free fibrin gels, encapsulating fibroblasts, demonstrated high elasticity and supported cell proliferation. This study underscores HA’s pivotal role in enhancing mechanical properties, presenting a promising method for effective skin regeneration. Collectively, these studies showcase the continuous evolution of HA in bioprinting, with each innovation contributing to the broader landscape of skin regeneration and wound healing.

#### 4.3.7. Pectin.

Pectin, a natural plant-derived polysaccharide, is gaining prominence in bioprinting due to its biocompatibility, biodegradability, and unique gelation properties in the presence of calcium ions [256]. This allows for the encapsulation of cells during bioprinting, supporting cell viability and activities. Pectin-based bioinks offer tunable mechanical properties, enabling the creation of constructs with specific characteristics to mimic diverse tissue micro-environments [257, 258]. Additionally, pectin facilitates controlled release of bioactive molecules, making it advantageous for incorporating growth factors in bioprinted constructs [259]. Ongoing research explores the potential of pectin, alone or in combination with other materials, for advanced applications in regenerative medicine and skin tissue engineering. Pereira *et al* developed thiol-norbornene-modified pectin for bioprinting, demonstrating tunable properties conducive to full-thickness skin formation [260]. Modified through thiol-norbornene chemistry, these cell-instructive hydrogels exhibited tunable properties, supporting the formation of full-thickness skin with distinct dermal and epidermal layers. The hydrogels, incorporating dermal fibroblasts and KCs, demonstrated fast gelation, cell spreading, and *in-vitro* skin formation. For wound healing, Jáuregui *et al* introduced an aerosol spray containing 0.1% w/v papain in 6% w/v pectin, showcasing enhanced stability and significant efficacy in a rabbit experiment [261]. The formulation’s success highlights pectin’s potential for enzymatic wound healing across various wound types. Turkkan *et al* explored the use of pectin in 3D scaffolds for skin tissue engineering [262]. Their scaffolds, composed of silk fibroin and citrus pectin, exhibited high porosity and supported fibroblast attachment, proliferation, and migration. This study emphasizes pectin’s role in designing scaffolds that promote essential cellular processes for skin regeneration. Overall, pectin emerges as a versatile material in regenerative medicine, demonstrating promise in bioprinting and skin tissue engineering applications.

#### 4.3.8. Alginate.

Alginate, a natural polysaccharide sourced from seaweed, has emerged as a pivotal polymer in the field of bioprinting [263, 264] Comprising repeating units of *β*-D-mannuronic acid and α-L-guluronic acid, alginate initially found utility in drug delivery and tissue engineering due to its biocompatibility and cost-effectiveness. Its significance in bioprinting lies in its rapid gelation ability upon exposure to cations (i.e. Ca^2+^) ions under physiological pH and temperature conditions. However, a drawback of alginate is its limited support for cell adhesion, attributed to the absence of cell adhesive sites. To address this, RGD is often incorporated as cell-binding molecules. As a result, alginate is commonly utilized in tandem with other hydrogels for applications in skin bioprinting. For example, Ramakrishnan *et al* developed an innovative alginate-based bioink (ALG-GEL-DCEL-FIB) tailored for skin bioprinting [265]. This bioink exhibited exceptional printability, precise shape retention, and sustained cellular viability during prolonged culture, leading to the creation of biomimetic tissue histology. Comprehensive assessments of its physicochemical (zeta potential, ion exchange capacity), rheological, and biocompatibility properties underscored the promising potential of alginate-based bioinks in advancing skin tissue biofabrication and therapeutic applications. In another study, Rezvanian *et al* engineered alginate composite films loaded with simvastatin for wound dressings [266]. Blending alginate with pectin or gelatin resulted in an alginate/pectin film with favorable wound dressing properties, superior mechanical strength, and controlled drug release. *In-vitro* cell viability assays indicated non-toxicity, suggesting the potential suitability of alginate/pectin formulations for simvastatin-based wound dressings. Comprehensive *in-vivo* studies are recommended for a thorough evaluation of toxicity and efficacy. Cheng *et al* delved into the properties of an alginate-gelatin-plantar dermis (Alg-Gel-PD) bioink and its impact on mouse MSCs [267]. In 2D, the bioink extract showcased enhanced cell migration and proliferation, coupled with partial differentiation towards sweat gland cells. In the realm of bioprinting, MSCs exhibited heightened proliferation, migration, and differentiation, accompanied by YAP1 activation. Hashimoto *et al* contributed to the field by developing alginate wound dressings incorporating hybrid peptides derived from laminin and elastin for wound healing [268]. These hybrid peptides promoted cell attachment and proliferation *in vitro*. Alginate dressings combined with these peptides significantly enhanced epithelialization and tissue regeneration in a rabbit ear skin defect model. This study underscores the potential of these alginate constructs in treating wounds with impaired healing, emphasizing their biocompatibility and suitability for bioprinting applications.

### 4.4. Bioprinting other skin components

#### 4.4.1. Bioprinting HFs and sweat glands.

The HF is a complex structure comprising two compartments: the epidermal compartment and the dermal compartment (figure 1(a-ii)). The interactions between these compartments are crucial for the development and growth of HFs [269, 270]. Effective communication between the dermal and epidermal cells is considered essential for successfully reconstituting HFs for research or therapeutic purposes. The dermal portion of HF can be further divided into two compartments: the dermal papilla and dermal sheath. The dermal papilla is located at the base of HFs, while the dermal sheath, also known as the connective tissue sheath, lines the epithelium of HF from the bulge level downwards. The dermal sheath is linked directly to the base of the dermal papilla through a stalk, with a basement membrane (BM) serving as a separator between the dermal papilla and dermal sheath from the epithelial segment on HF [271].

Additionally, skin pigmentation relies on the transfer of melanin from MCs to surrounding KCs, primarily occurring within KCs themselves [272]. The ratio of MCs to KCs in the native skin is approximately 1:20, with a minimum density of 1 × 10^4^ MCs cm^−2^ needed for full pigmentation restoration [273]. Co-culturing KCs and MCs enhances MC differentiation, migration, and proliferation through KC-derived growth factors [274]. Proper attachment of MCs to the epidermal-dermal junction requires BM components, which also regulate tyrosine uptake for melanin synthesis [275, 276]. Bioprinting allows for the precise deposition of BM components, promoting crucial cell interactions between adjacent bioprinted cell layers.

In the literature, various models have been established to study the interactions between the dermal and epidermal compartments, as well as for the reconstitution of HFs [277–279]. Most hair reconstitution experiments were conducted on immunodeficient mice [280] or utilize mouse cells [281, 282] to achieve HF formation, the use of 3D printed molds has facilitated the development of a controlled organization and microenvironment favorable for skin construction. Abaci *et al* used 3D printing to design vascularized human skin constructs that combine fibroblasts, KCs, and dermal papilla cells to drive HF differentiation [283]. The spatial arrangement resembling native HF organization allows for HF organization within skin constructs, as well as successful induction when grafted onto immunodeficient mice [283]. The ability to generate HFs from human cells *in vitro* can be advantageous for the development of more physiologically-relevant skin models, as well as it shows potential for regenerative medicine.

**Figure 3.**
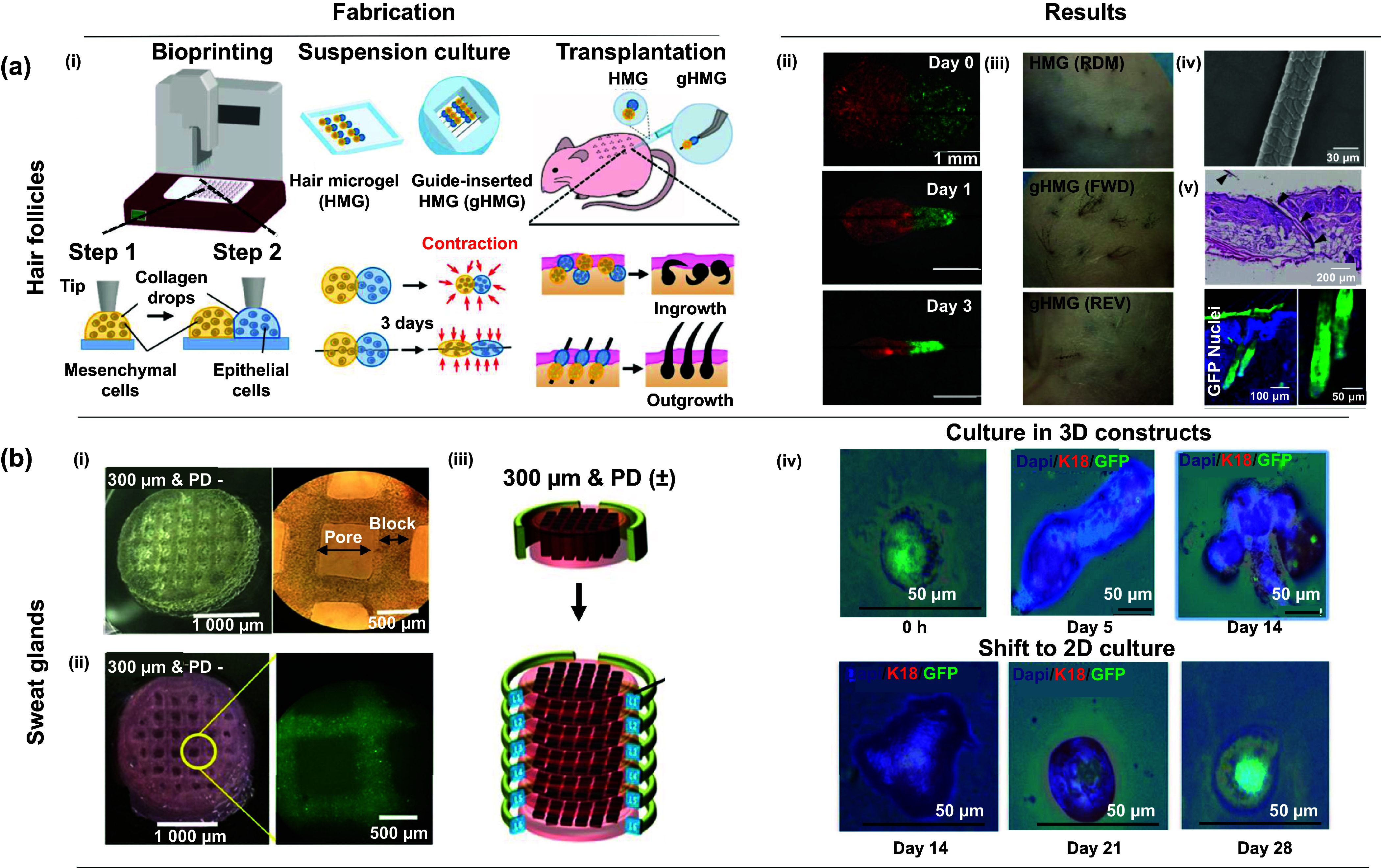
Examples of 3D bioprinting for hair follicles and sweat glands. (a) Bioprinted microgels for hair regeneration, showing cell arrangement, and hair formation in nude mice. (i) Microgels for hair (HMGs) and guide-inserted HMGs (gHMGs) were created through bioprinting. Sets of collagen droplets containing mouse embryonic mesenchymal and epithelial cells were arranged side by side and sequentially crosslinked. In the subsequent suspension culture, the contracted microgel beads, termed HMGs, exhibited increased collagen and cell density after 3 d. Hair-inducing potential of HMGs was assessed by transplanting them into the dorsal skin of nude mice. Similarly, gHMGs were produced by placing collagen droplets on aligned surgical suture guides and their effects were studied post-transplantation into nude mice, (ii) microgel beads underwent spontaneous contraction along the guide during a 3 d culture. Merged fluorescent and phase-contrast microscope images were used, and Vybrant DiI and DiO cell-labeling solutions were employed to differentiate mesenchymal and epithelial cells, (iii) three different tissue grafts resulted in the generation of hair shafts. The dorsal skin of nude mice was observed 3 weeks after transplantation to assess the outcomes, (iv) regenerated hair shafts were captured through scanning electron microscopy 3 weeks post-transplantation of gHMGs, (v) newly formed HFs were observed in the forward direction 3 weeks after gHMG transplantation. Skin cross-sections were visualized using H&E staining. Green fluorescent protein (GFP)^+^ cells, representing nuclei in blue and donor cells in green, were evident in the regenerated HFs. Reproduced from [286]. CC BY 4.0. (b) Porous constructs displaying cell distribution and differentiation into sweat gland cells. (i) Porous construct architectures, both block and pore, were identified immediately after printing, (ii) distribution of embedded cells and the structural stability of printed constructs were assessed after a 24-h culture, (iii) cell distribution on each layer of the scaffold was examined, (iv) epidermal progenitor cells embedded in constructs underwent differentiation into sweat gland cells, confirmed by immunostaining with K18 at Day 5, and aggregation into sweat gland-like structures was observed at Day 14. All markers appeared in red and DAPI staining of nuclei was represented in blue. Upon transition to 2D culture, the structure disappeared by Day 21, and the number of differentiated cells decreased by Day 28. Reproduced from [290]. CC BY 4.0.

The field of regenerative medicine has witnessed significant strides in the bioprinting of skin appendages, with a particular focus on HFs. Studies, such as Abaci *et al*, Zhao *et al*, Chen *et al*, Kang *et al* [65], and Nanmo *et al*, showcase innovative approaches in leveraging bioprinting technologies for the regeneration of HFs (figure 3(a)) [283–287]. Following the approach developed by Abaci *et al* for HF differentiation, Catarino *et al* used EBB to create a complex HF model [288]. Initial bioprinting of dermal papilla cells and HUVECs allowed for spheroid formation, and subsequent bioprinting of KCs and MCs resulted in an organization like the native HF [288]. These studies demonstrate the creation of biomimetic scaffolds, the use of adaptive robots for precise bioprinting, and scalable automated methods for hair-inducing tissue grafts. These advancements hold considerable promise for addressing HF disorders and revolutionizing the treatment of hair loss.

Additionally, the regeneration of sweat glands, a challenging yet crucial aspect of skin appendage reconstruction, has been explored through bioprinting. Huang *et al*, Liu *et al*, Wang *et al*, Zhang *et al* and recent efforts employing 3D bioprinting have contributed to our understanding of the microenvironmental cues necessary for sweat gland differentiation and regeneration (figure 3(b)) [289–292]. These studies emphasize the importance of tailored bioinks, controlled pore structures, and artificial microenvironments in guiding stem/progenitor cell fate toward functional sweat glands. The evolving field of bioprinting provides not only insights into the regeneration of specific skin appendages but also opens avenues for broader applications in tissue regeneration through microenvironmental manipulation.

#### 4.4.2. Bioprinting for skin color and pigmentation.

One of the new application areas of 3D bioprinting within the context of skin reconstruction is skin pigmentation and coloration similar to that of the native human skin. This application can be considered for a variety of purposes, from addressing aesthetic issues to treating diseases like vitiligo, which is characterized by patches of white skin where the skin’s pigmentation has been lost [293, 294]. The goal of bioprinting forskin pigmentation is to duplicate the skin’s natural color. Melanin, the pigment responsible for skin color, is produced as a part of this process. It is essential to achieve a lifelike appearance for both cosmetic and medical reasons. Vitiligo sufferers are one of the main medical populations for which skin color bioprinting can be used [294]. By producing skin grafts with the proper pigmentation to match the patient’s unaffected skin, bioprinting can offer a remedy. MCs are added to bioinks and then precisely deposited to produce the desired pigmentation pattern [295]. It is still difficult to get stable and realistic pigmentation in the bioprinted skin. Research is still being done in the areas of preserving color over time, ensuring even distribution, and dealing with problems related to changes in pigmentation (such as tanning). In terms of body image, societal standards of beauty, and the potential for abuse, the cosmetic use of bioprinting for skin tone raises ethical concerns. Future outcomes are probably going to be better thanks to developments in 3D cell culture methods, bioprinting technologies, and our knowledge in skin pigmentation. The idea of receiving customized care that is specifically matched to a person’s needs and skin tone is intriguing.

In this regard, Li *et al* showed the presence of dermal stem cells (DSCs) acting as a reservoir for epidermal MCs. These multipotent stem cells demonstrated the ability to migrate to the epidermis and differentiate into MCs [296]. Building on this discovery, they developed 3D human skin constructs, incorporating normal human MCs, DSCs, and melanoma cells. Unlike traditional 2D culture, 3D skin constructs successfully recapitulated stage-specific properties of melanoma cells, showcasing their potential in MC homeostasis studies [297]. However, manual casting led to non-uniform pigmentation [298]. To address this limitation, Ng *et al* pioneered a two-step DBB process, offering precision in cell deposition and overcoming manual casting challenges. The process involved bioprinting fibroblast-laden collagen, followed by direct bioprinting of KCs and MCs in a controlled manner. This resulted in bioprinted pigmented human skin constructs exhibiting uniform pigmentation, precise cell distribution, and hierarchical porous structures, presenting a stark improvement over the manual casting method [9]. The transition from 2D to 3D skin models, facilitated by advancements in bioprinting technologies, marks a revolution in skin biology and pathology studies. The ability to mimic native skin structures with precision and uniformity via bioprinting is a major strength in advancing our understanding of skin-related processes and diseases *in vitro*.

### 4.5. Utilization of iPSCs

In regenerative medicine and skin research, iPSCs are a useful tool for researching skin development, simulating illnesses, and developing customized cell treatments because of their capacity to be reprogrammed into a variety of cell types, such as KCs, fibroblasts, and MCs [299]. iPSCs provide a platform to comprehend skin biology, screen for therapeutic efficacy and toxicity, and possibly provide autologous cell sources for skin transplants. They also offer a scalable and patient-specific method. The adaptability of iPSCs has great potential for the creation of novel and customized therapies in dermatology, and it considerably advances our understanding of skin-related illnesses [300].

Researchers, exemplified by Itoh *et al* [301, 302], have successfully generated human 3D skin equivalents (HSEs) using iPSC-derived KCs, fibroblasts, and MCs [301, 302]. The incorporation of a functional iPSC-derived epidermal-melanin unit in these HSEs represents a significant advancement in disease modeling, offering a platform to study skin biology and drug responses. However, challenges such as low MC numbers and the need for *in-vivo* investigations warrant further exploration and optimization. Furthermore, addressing the crucial role of innervation in skin diseases, Muller *et al* established the development of innervated tissue-engineered skin constructs by differentiating neurons from iPSCs [303]. This innovative approach mimics the complexity of native skin, providing a physiologically-relevant environment for studying sensory neuron behavior. The functional relevance demonstrated by iPSC-derived neurons, including substance P release and action potential generation, highlights the potential of this model for exploring the complex interplay between sensory neurons and skin tissue in various skin patho-physiologies. These iPSC-based models not only enhance our understanding of skin diseases but also offer promising platforms for drug discovery and personalized medicine. Guo *et al* [304] successfully engineered a human skin model innervated with itch sensory neuron-like cells derived from iPSCs [304]. These neurons responded specifically to itchy stimuli and cytokines related to skin inflammation. The work provides a platform for studying skin diseases and testing therapeutic agents.

While iPSC-derived KCs and fibroblasts are commonly studied, protocols for differentiation of other relevant cell types of the skin have been developed in recent years. Due to the importance of vasculature in skin constructs, the ability to differentiate endothelial cells (ECs) from iPSCs presents an alternative to primary ECs for skin tissue engineering [305–307]. However, iPSC-derived ECs (iECs) display immaturity and a lack of vascular network self-organization in skin constructs *in vitro*, compared to primary ECs [305]. Similarly, decreased vessel density and immaturity of iECs were observed *in vivo* [306], indicating that iEC differentiation protocols should be further optimized. To develop models that better recapitulate skin conditions, other cell types can be differentiated and incorporated into 3D constructs. Protocols for the generation of iPSC-derived brown and white adipocytes have already been established and show similar characteristics to primary adipocytes when transplanted into mice [308, 309]. Additionally, a variety of immune cells have also been successfully differentiated from iPSCs, including NK cells, macrophages, and T cells [310, 311]. Development of more complex skin constructs through the incorporation of a variety of iPSC-derived skin cell types holds potential for customized disease modeling and drug discovery. Since iPSCs are reprogrammed from small tissue biopsies and expanded in culture, they can provide multiple skin cell types to form physiologically-relevant constructs that model diseases in a personalized fashion. Primary fibroblasts from recessive dystrophic epidermolysis bullosa have been reprogrammed to iPSCs and corrected through CRISPR/Cas9 to restore collagen VII in hSKEs [312]. Organoid differentiation from iPSCs represents another model for studying disease and development. Lee *et al* developed a protocol for the differentiation of human iPSCs into hair-bearing skin organoids, which could also be useful as a cell source for skin regeneration [313] and skin tissue engineering. Nonetheless, the structure of these organoids does not resemble normal skin, as the hair bulbs protrude outwards, and the core contains the most cornified tissue [313]. Additionally, skin organoids lack complex vasculature and neural networks, as well as other cell types such as immune cells [313, 314].

Former studies show the ability to bioprint undifferentiated iPSCs that retain their pluripotency and differentiation potential, as well as to subsequently differentiate them into cartilage, cardiac, and neural tissues after bioprinting [315–317]. These studies present the potential for bioprinting viable iPSCs that can be differentiated into skin cell types to create customized skin constructs. Previously, human iPSC-derived mesenchymal stem cells (hiMSCs) have been bioprinted to form a hydrogel scaffold that improves the recovery of wounded endometrium [318]. Additionally, Lin *et al* demonstrated that hiMSCs can be induced with KGF to differentiate into epidermal-like cells [319]. Therefore, bioprinting hiMSCs followed by epidermal differentiation could be used to develop iPSC-derived skin constructs. Bioprinting of differentiated iPSCs has also been achieved for constructs including cardiac and hepatic tissues, but not for skin. A combination of iPSC-derived cardiomyocytes, primary fibroblasts, and primary ECs was bioprinted to form tubular cardiac constructs [320] and iPSC-derived hepatic cells have been bioprinted to form a liver lobule-like model [321]. While iPSC-derived skin cell types have not been used to bioprint skin constructs, optimization of bioprinting conditions and methods could result in physiologically-relevant models. Autologous iPSC-derived skin constructs also show potential for regenerative medicine due to the reduced risk of immune rejection and the ability to generate tissues from a few reprogrammed cells [322].

### 4.6. Vascularization of skin

One of the major obstacles in tissue engineering for skin reconstruction is the vascularization of the skin, which is crucial for developing blood vessels essential to maintaining tissue health [323, 324]. For bioprinted skin constructions to be viable over the long term, it is imperative to create a functioning circulatory network. Complex and networked vascular networks required for the delivery of nutrients and oxygen are usually difficult to create using conventional techniques. 3D Bioprinting appears to be a viable way to address this issue [325]. By precisely stacking cells and biomaterials, this cutting-edge method makes it possible to create complex vascular networks. Through the strategic and localized organization of cell deposits, bioprinting can replicate the vascular architecture seen in the native skin tissue. This leads to the production of more functional and realistic skin replacements by promoting efficient nutrition exchange and strengthening the structural integrity of the bioprinted skin [326, 327]. Bioprinting can overcome the long-standing obstacle of vascularization, hence propelling the field of skin tissue engineering towards more sustainable and therapeutically applicable solutions [328].

**Figure 4.**
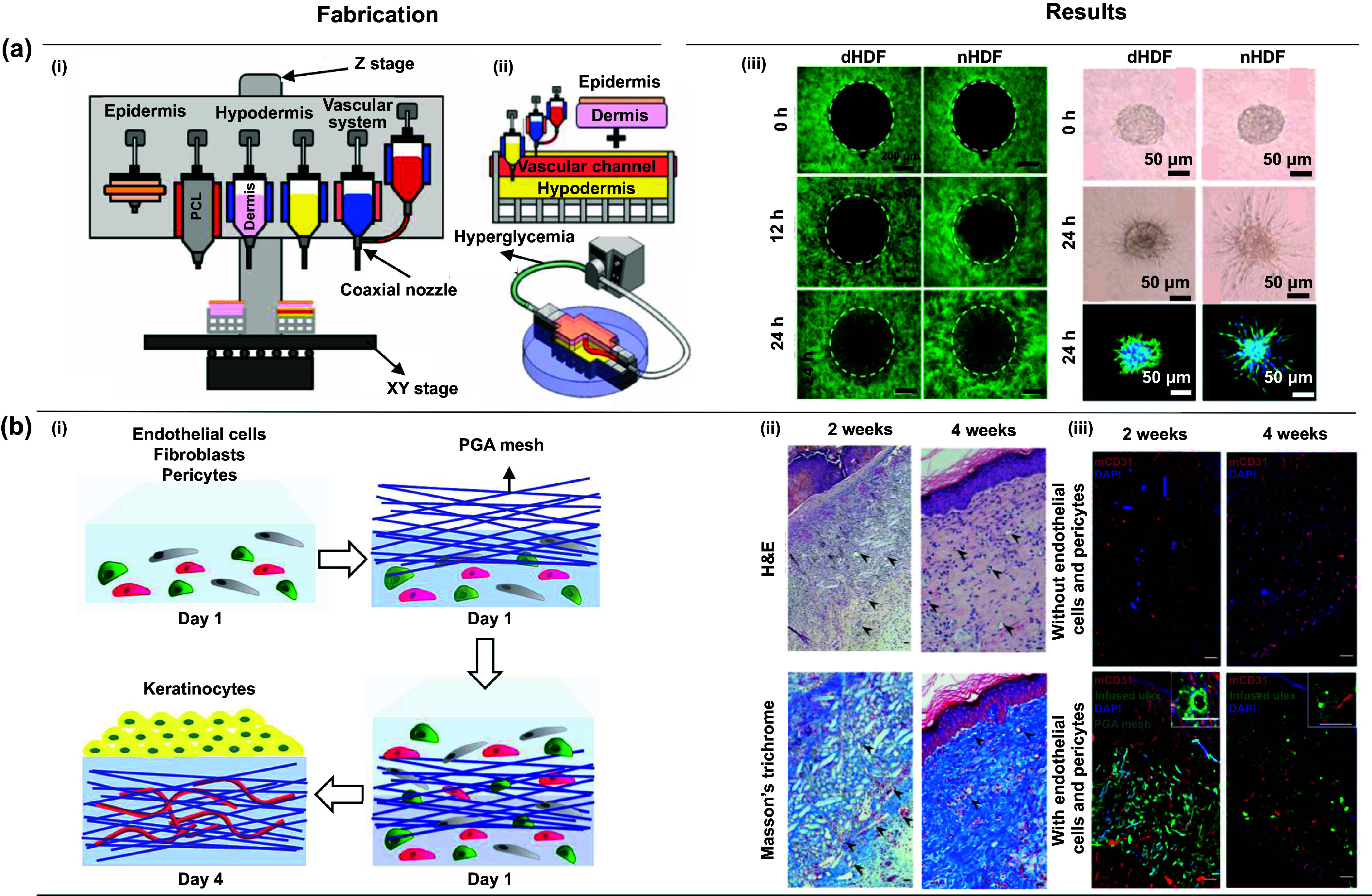
Examples of 3D bioprinting for vascularizated skin tissues (a) Custom bioprinting system for diabetic skin modeling, and HUVEC spheroid spreading. (i) A custom-made bioprinting system was utilized, allowing the simultaneous application of EBB and associated modules to engineer the complexity of diseased human skin, (ii) diabetic epidermis was modeled by fostering intercellular interaction between diabetic human dermal fibroblasts (dHDFs) and normal human epidermal KCs (nHEKs), (iii) calcein-AM staining images were employed to assess and compare the degree of wound healing, (iv) the sprouting ability of HUVEC spheroids was evaluated through immunofluorescent staining, revealing the results. Reprinted from [331], © 2021 Elsevier Ltd All rights reserved. (b) Illustration of xeno-free skin graft bioprinting, highlighting microvessel formation and host angiogenesis. (i) An illustration demonstrating the bioprinting of xeno-free human skin grafts, (ii) evaluation of xeno-free skin grafts with ECs and pericytes (PCs) at 2- and 4 week intervals post-engraftment onto immunodeficient mice. Representative images of H&E and Masson’s trichome-stained sections illustrate the degradation of the polyglycolic acid (PGA) mesh after 2 weeks. Scale bars indicate 100 *μ*m, (iii) assessment of microvessel formation in grafts derived from the graft and host in xeno-free skin grafts with and without ECs and PCs at 2- and 4 weeks post-engraftment onto immunodeficient mice. CD31 Staining highlights a higher degree of host angiogenesis (depicted in red) and the presence of perfused human EC-lined vessels (shown by infused Ulex staining) in grafts containing ECs and PCs. Reproduced from [335]. CC BY 4.0.

There are two main categories of vascularization methods: use of angiogenic biomaterials and prevascularization constructs. Angiogenic biomaterials stimulate the growth of blood vessels into implanted tissue constructs. However, the slow growth rate of newly formed micro vessels, at only 5 *µ*m·h^−1^, makes them unsuitable for rapid vascularization of larger implants [323–329]. On the other hand, prevascularization of constructs aims to establish microvascular networks within tissue constructs before implantation. These networks merge with existing blood vessels at the implantation site, leading to improved blood supply [330]. Bioprinted patient-derived skin substitutes can be used to model disease conditions *in vitro*. For example, Kim *et al* used a combination of EBB and DBB to develop a diabetic skin model using patient-derived fibroblasts and diabetic KCs, and coaxial bioprinting was used to incorporate a perfusable vascular system [331]. The model recapitulated the disease hallmarks, such as delayed wound healing and increased insulin resistance, and showed restored function of the epidermis when treated with test drugs (figure 4(a)) [331]. The findings from this study confirm the effectiveness of the platform for modeling skin diseases and testing therapeutic interventions in a more physiologically- relevant environment.

Bioprinting has also been used to develop vascularized skin substitutes that promote wound healing *in vivo*. Dai *et al* used EBB to create a dermo-epidermal substitute with fibroblasts, KCs, and EPCs [332]. Vascularization through the incorporation of EPCs in the dermal layer improved wound healing when the substitute was grafted into skin wounds on immunodeficient mice due to microvessel formation and matrix organization [332]. EPCs have also been used in combination with ADSCs to promote neovascularization in a bioprinted skin patch [333]. Regulating the microenvironment during pre-vascularization of the skin patch resulted in improved wound healing, neovascularization, and blood flow when implanted into immunodeficient mice [333]. Proper vascularization of skin equivalents dictates the success of the constructs for *in-vivo* applications, and the use of EPCs may offer an alternative to primary ECs.

A recent study by Rimal *et al* has showcased the remarkable progress in the development of scaffold-free vascularized hSKEs [334]. The innovative approach, incorporating techniques such as cell coating and accumulation, demonstrated enhanced tissue viability, improved epidermal barrier properties, and preserved vessel network morphology. The utilization of 3D printed bioreactors with continuous media flow to the dermal compartment represents a significant advancement, allowing for dynamic flow regulation that effectively controls angiogenesis and prevents dermal degradation. The findings from these studies provide a valuable platform for modeling skin diseases and testing therapeutic interventions in a more physiologically-relevant environment.

Moreover, investigations by Baltazar *et al* [335] and Oliveira *et al* have addressed critical challenges in vascularized skin constructs, including mesh material selection and transplantation methods (figure 4(b)) [335, 336]. In another study, Karande *et al* have engineered an innovative bioink, comprising human endothelial and pericyte cells alongside collagen, to fabricate constructs capable of forming biologically-relevant vascular structures [337]. This milestone underscores the potential of 3D bioprinting in precision medicine, offering bespoke solutions for wound healing by fostering the integration of synthetic grafts with the patient’s skin tissue. These collective advancements contribute significantly to the evolving landscape of vascularization strategies in skin tissue engineering. They not only enhance the functionality of *in-vitro* skin models but also open promising avenues for the development of advanced therapeutic interventions and a deeper understanding of skin-related pathologies.

### 4.7. Clinical translation of bioprinted skin

Clinical translation of bioprinted skin represents a paradigm shift in dermatological care, holding immense potential for personalized and precise interventions. The promising translation of skin bioprinting from academic research to clinical practice opens various potential applications in regenerative medicine, including cell therapy (such as cell-based immunotherapy and stem cell therapeutics) and tissue engineering [338, 339]. Recent findings suggest that bioprinting holds potential for regenerating not only the skin tissue but also its appendages [2, 340]. A critical clinical need is the demand for skin grafts. Bioprinting a skin graft emerges as a viable alternative to the conventional, often painful approach of skin grafting. This innovative method aims to minimize donor requirements and improve the overall effectiveness of skin graft treatments. The application of dermal substitutes, particularly those based on collagen/elastin matrices, has emerged as a promising strategy for addressing the challenges associated with extensive burns, where limited donor sites for autografts are available [341–343]. The studies conducted by Demircan *et al* and Ho *et al* highlight the efficacy of these dermal substitutes in facilitating wound closure and minimizing scarring in patients with significant burn injuries [344, 345]. A bovine-derived collagen/elastin matrix utilized by Demircan *et al* demonstrated exceptional outcomes, with successful graft survival and notably low Vancouver scar scale scores, indicative of minimal scarring [345]. Additionally, Ho *et al* reported a double-layered tissue-engineered skin, composed of a collagen matrix embedded with fibroblasts and an epidermal layer, showcasing accelerated wound healing and formation of skin closely resembling the native skin [344]. These findings underscore the potential of dermal substitutes as valuable tools in reconstructive procedures for extensive burns, offering improved outcomes and reduced scarring compared to traditional approaches.

Cubo *et al* presented a pioneering study in the clinical translation of 3D bioprinted human skin, addressing critical limitations in conventional methods [101]. Their innovative approach, utilizing bioinks comprising human plasma and primary cells, facilitated the rapid production of skin substitutes closely resembling natural human skin. Impressively, they achieved bioprinting of 100 cm^2^ of skin in under 35 mins, a substantial improvement in efficiency over manual techniques. Through meticulous histological and immunohistochemical analysis, the researchers confirmed the structural fidelity and functional capacity of the bioprinted skin, including proper differentiation and vascularization. Moreover, the potential for cost reduction and scalability through automation and standardization positions this method for widespread clinical adoption and industrial production. The work by Liu *et al* also represents a significant step forward in the clinical translation of bioprinted human skin, offering promising prospects for wound healing and safety assessment in clinical settings [346]. By formulating a bioink comprising gelatin, sodium alginate, and fibrinogen, the study addresses crucial challenges in conventional methods, such as stability and robustness. Their four-step strategy for fabricating full-thickness skin, combined with histological and immunohistochemical analysis, demonstrates the construct’s resemblance to the native skin. While promising wound healing and safety testing, further optimization is needed to enhance complexity and functionality.

The 3D bioprinted skin grafts described by Baltazar *et al* closely resemble native human skin in structure, function, and molecular composition [347]. By incorporating human KCs, fibroblasts, pericytes, and ECs into the bioink, they successfully replicated the multilayered architecture of human skin, complete with a vascularized dermal layer and an epidermal layer. *In vitro* studies demonstrated the formation of interconnected microvascular networks and the maturation of KCs to form a barrier similar to the native skin. Moreover, when implanted in immunodeficient mice, the grafts integrated with the host, formed perfused vascular networks, and displayed features characteristic of the human skin, such as rete ridges and BM formation. The clinical translation of 3D bioprinted skin holds significant promise for the treatment of nonhealing cutaneous ulcers and other skin disorders. These bioengineered skin grafts offer a potential solution to the limitations of current avascular substitutes by providing a vascularized dermal layer crucial for long-term engraftment and integration with host tissue. Furthermore, the use of allogeneic cells derived from easily accessible sources, such as cord blood and adult peripheral blood, facilitates large-scale production and may enable the development of off-the-shelf skin grafts for clinical use. While challenges remain, such as immune rejection of allogeneic cells, advancements in genetic modification techniques hold the potential to mitigate these risks and pave the way for the widespread clinical application of 3D bioprinted skin substitutes.

The standard treatment for severe skin wounds is skin grafting but efforts to develop alternative solutions have been ongoing for years. The goal is to create substitutes that resemble the native skin, containing all its components. However, most engineered skin products are still in early research stages, including 3D bioprinted constructs and matrix-cultured products. While some assist wound healing, they do not fully replicate the native skin and often require additional procedures. Bioprinting shows promise in avoiding donor site issues and creating personalized skin substitute, but its clinical use is limited due to cost and variable outcomes. Developing innovative healthcare technologies entails high initial costs. However, advancements in technology and increased market competition may eventually reduce these costs. Nonetheless, current 3D skin bioprinting remains more expensive than conventional dermal substitutes and autologous skin grafts, which are approximately $2000 and $17 000, respectively [348, 349].

Nevertheless, despite the promising outlook, it is crucial to recognize that skin bioprinting is still in its early stages of clinical development. The integration of automated standardized processes is necessary to effectively translate bioprinted skin into clinics. Numerous challenges, including experimental, ethical, budgetary, and regulatory hurdles, impede the swift implementation of this technology in clinical practices.

The growing interest of skincare companies in 3D bioprinting signifies a revolutionary approach to testing cosmetic and pharmaceutical products. This innovative technology, capable of arranging cells in a physiologically-relevant manner, offers advantages for both industries. In pharmaceuticals, 3D bioprinting allows for *in-vitro* testing of medicines and chemicals, ensuring safety before clinical studies. In cosmetics, it streamlines the evaluation of potential toxic and allergic effects, expediting the testing process [350, 351]. This transformative technology not only makes drug and product testing faster and more cost-effective but also introduces ethical considerations with standardized and automated methods. Bioprinted skin models, representing various skin types, are crucial for comprehensive cosmetic testing [293, 352].

On the other hand, clinical translation of skin products, especially those incorporating innovative components, faces several challenges. The scientific, regulatory, and ethical domains provide a variety of difficulties for the clinical translation of skin care products. A robust regulatory framework encompassing quality assurance measures is imperative, covering various stages from model design to post-bioprinting evaluation before transplantation. One of the most significant difficulties is the stringent regulatory oversight exercised by health authorities, including the Food and Drug Administration (FDA), which requires extensive proof of safety and effectiveness prior to authorizing a product for clinical use [353–355]. Recently, the FDA issued guidelines on ‘Technical Considerations for Additive Manufactured Devices’, applicable to medical devices, which also extend to bioprinted skin products [356, 357]. Skin tissue engineered through bioprinting is often classified as a combination product, which involves pharmaceuticals, medical devices, and biologics. Depending on their composition, surgically implantable engineered tissues are regulated by the FDA either as devices or biologics and require thorough testing in clinical trials before use by surgeons. Stem cell-based products used in skin bioprinting are classified as somatic cellular therapies and are subject to FDA guidelines for human cell, tissue, and cellular- and tissue-based products. These guidelines, outlined in Part 1 271 of the Public Health Act, establish stringent requirements for donor eligibility and procedures for stem cell handling, distinct from traditional Good Manufacturing Practices guidelines. While engineered tissues used for research purposes may not require FDA approval during initial testing phases, certain regulations govern their shipping and disposal as outlined in Title 21 of the Federal Code of Regulations. Overall, adherence to these regulatory standards is crucial to ensure the quality, safety, and efficacy of bioprinted skin products for clinical use in burn reconstruction [356]. It is a continuous challenge to ensure the long-term safety of these goods, especially those that use innovative technologies, and to conduct thorough examinations into potential side effects and allergenicity [358]. Another difficult task is standardizing production procedures to ensure repeatability and constant product quality [359]. Furthermore, it is essential yet sometimes difficult to design successful clinical trials and have the right patient groups and objectives [360]. To successfully negotiate the complex road from discovery to clinically viable and morally sound skin products, researchers, industry stakeholders, and regulatory agencies must work together.

### 4.8. Other emerging applications

Bioprinting has ushered in a new era of innovation in disease modeling and cancer research within the realm of skin studies (figure 1(d)). Through the incorporation of 3D tumor models, researchers gain a comprehensive understanding of cancer proliferation, metastasis, and responses to therapeutic interventions. An example of this capability is evident in the introduction of melanoma into human *in-vitro* skin equivalents, showcasing the adaptability and relevance of bioprinting in this field.

In 2020, Liu *et al* introduced a reproducible bioprinting technique for creating hSKEs with varying cellular complexity, including vascularized full-thickness skin models [361]. This innovation allowed for the modeling of atopic dermatitis (AD) and the evaluation of preclinical relevance through testing anti-inflammatory drugs. The results demonstrated enhanced physiological relevance, offering a scalable approach for high-throughput drug screening in a clinically-relevant 3D tissue microenvironment. Building upon this progress, Lègues *et al* in the same year presented a 3D bioprinted immune skin model for efficient drug and ingredient screening, catering to both normal and inflamed skin conditions [362]. The model exhibited robust structure, reproducibility advantages, and effective assessment of immune response, making it a promising platform for safety and efficacy screening, particularly in addressing skin inflammation associated with global pollution. In 2022, de Andres *et al* took a significant step forward by developing a bioprinted malignant melanoma model that faithfully replicated the tumor microenvironment [363]. The tri-layered constructs showcased distinct drug responses compared to traditional 2D culture. Upon implantation into mice, the model demonstrated representative tumor growth, establishing itself as a valuable platform for drug screening and advancing melanoma research. Around the same time, Browning *et al* [364] bioprinted a skin model for cutaneous squamous cell carcinoma (cSCC) [364]. Validated through various analyses, this model allowed non-destructive assessment of chemotherapeutic effects, providing valuable insights for cancer research and drug development in cSCC. Expanding the scope of melanoma research, Duan *et al* bioprinted GelMA/PEGDA (polyethylene (glycol) diacrylate) constructs to establish an *in-vitro* melanoma model [365]. These constructs exhibited favorable mechanical properties and biocompatibility, with melanoma cells displaying enhanced proliferation and differentiation in 3D compared to traditional 2D culture. Importantly, the study highlighted the utility of the 3D bioprinted model for drug testing, emphasizing the increased resistance of A375 cells to the anti-cancer drug luteolin in 3D compared to 2D.

Developing advanced *in-vitro* systems that accurately replicate human skin is also crucial for other emerging areas, such as microbiome studies [366]. Challenges with human skin explants, such as preservation, availability, and variability, limit their widespread use in microbiome research. Therefore, 3D bioprinted models offer insights into permeation, safety, and toxicology, given the skin’s role as a robust barrier. Integrating the complete human skin microbiota into these models is vital for enhancing skin cell growth and cohesion, ultimately improving barrier function. Ensuring the inclusion of microbiota communities, including features like sebaceous glands, will be also crucial. These models hold great promise for exploration of interactions among immune cells, skin tissue and the microbiome.

### 4.9. The incorporation of biosensor technologies

The fusion of skin bioprinting with biosensors not only advances the capabilities of regenerative medicine but also heralds a revolutionary approach to wound healing and personalized healthcare. In the realm of regenerative medicine, this innovative integration creates a dynamic interplay between engineered smart skin tissue and advanced biosensing capabilities. The incorporation and integration of biosensors into bioprinted smart skin tissues bring forth a new era in healthcare. Techniques such as incorporating biosensors into the bioink or integrating them seamlessly with the bioprinted skin enable real-time monitoring of physiological parameters. This active monitoring goes beyond mimicking human skin; it actively modulates the healing process, providing opportunities for more efficient and patient-centered regenerative medicine.

The biosensors utilized in this multimodal approach include resonant biosensors, optical biosensors, thermal detection biosensors, ion-sensitive biosensors, and electrochemical biosensors. Each type offers unique advantages in detecting various analytes and contributes to precise tissue engineering. This integration of diverse biosensor types with bioprinted smart skin tissues facilitates real-time monitoring of wound healing processes. Moreover, this approach allows for adaptive responses to changing variables within the wound environment while simulating the native skin structures. This significant advancement in healthcare holds the promise of more effective, patient-centered regenerative medicine. In a related breakthrough, the development of a novel ultrastable plasmonic bioink for point-of-care biosensors, as presented by Yin *et al*, further contributes to this future [367]. This bioink, featuring antibodies encapsulated in an organosiloxane polymer, ensures exceptional stability, and serves as a sensitive nanotransducer for label-free biochemical detection. Plasmonic biochips fabricated with this bioink demonstrate superior stability, marking a significant step forward in cost-effective, multiplexed biosensor production for early disease diagnosis.

Over the past few decades, various biosensors have emerged, each playing a crucial role in this transformative process. Among them, resonant biosensors utilize acoustic wave transducers and bioelements, typically antibodies, to detect changes in membrane mass when reacting with an analyte [368]. Optical biosensors, another common class, leverage light and diffraction gradients to amplify reactions with bioelements, enhancing sensitivity [369]. Thermal detection biosensors, measuring molar enthalpy changes, find primary application in sensing pesticides and pathogenic bacteria. Ion-sensitive biosensors, employing field-effect transistors, detect charged analytes by monitoring surface electrical potential changes, commonly used for detecting pH variations [370]. The electrochemical biosensor, measuring electrons/ions produced or consumed in a reaction, offers diverse electrical measurements through various subclasses.

The integration of these diverse biosensor types with bioprinted smart skin tissues holds immense potential for real-time monitoring of wound healing processes. This multimodal approach, combining precise tissue engineering with biosensing technologies, enables adaptive responses to changing variables within the wound environment while faithfully simulating the native skin structures. This significant advancement in healthcare not only enhances the efficiency of regenerative medicine but also fosters a patient-centered approach, paving the way for a more personalized and effective future in healthcare.

#### 4.9.1. Biosensors for wound healing and regenerative medicine.

Biosensors play a crucial role in wound healing and regenerative medicine, providing essential capabilities for early detection of biomarkers associated with inflammation, infection, and tissue repair [371]. These devices offer real-time monitoring, enabling healthcare providers to optimize treatment strategies based on individual patient responses [372]. By facilitating remote monitoring, biosensors empower patients to recover at home while ensuring healthcare professionals receive timely data for proactive intervention. The objective and quantitative assessment of wound healing progress offered by biosensors reduces reliance on subjective evaluations, enhancing accuracy in gauging actual healing status [373]. In regenerative medicine, these sensors contribute to the development of customized therapies by monitoring molecular and cellular events critical to tissue regeneration [374]. Biosensors not only improve patient outcomes by preventing complications and promoting personalized care but also hold the potential to reduce healthcare costs associated with prolonged hospitalizations and complications [375].

Different wound healing stages are characterized by specific biomarkers such as pH, temperature, bacterial infection, and tissue oxygenation, offering valuable insights into the wound status [376]. Monitoring these biomarkers, individually or in combination, is crucial for effective wound management [377]. The pH of wound exudate serves as a significant biomarker, offering insights into infection and healing status [378]. Acute wounds typically maintain an acidic environment to inhibit pathogenic microorganism growth and promote cell proliferation and tissue remodeling [379]. In contrast, chronic wounds exhibit an alkaline environment, fostering pathogenic bacterial growth [380]. Chronic wounds, posing significant healthcare challenges globally with high morbidity and mortality rates, benefit from accurate, timely detection of infections and real-time monitoring of wound healing biomarkers. Monitoring wound bed pH can aid in detecting bacterial colonization and initiating timely interventions. Various pH sensors, including colorimetric and electrochemical types, have been integrated into bandages for healthcare applications, addressing different aspects of wound monitoring [381].

**Figure 5.**
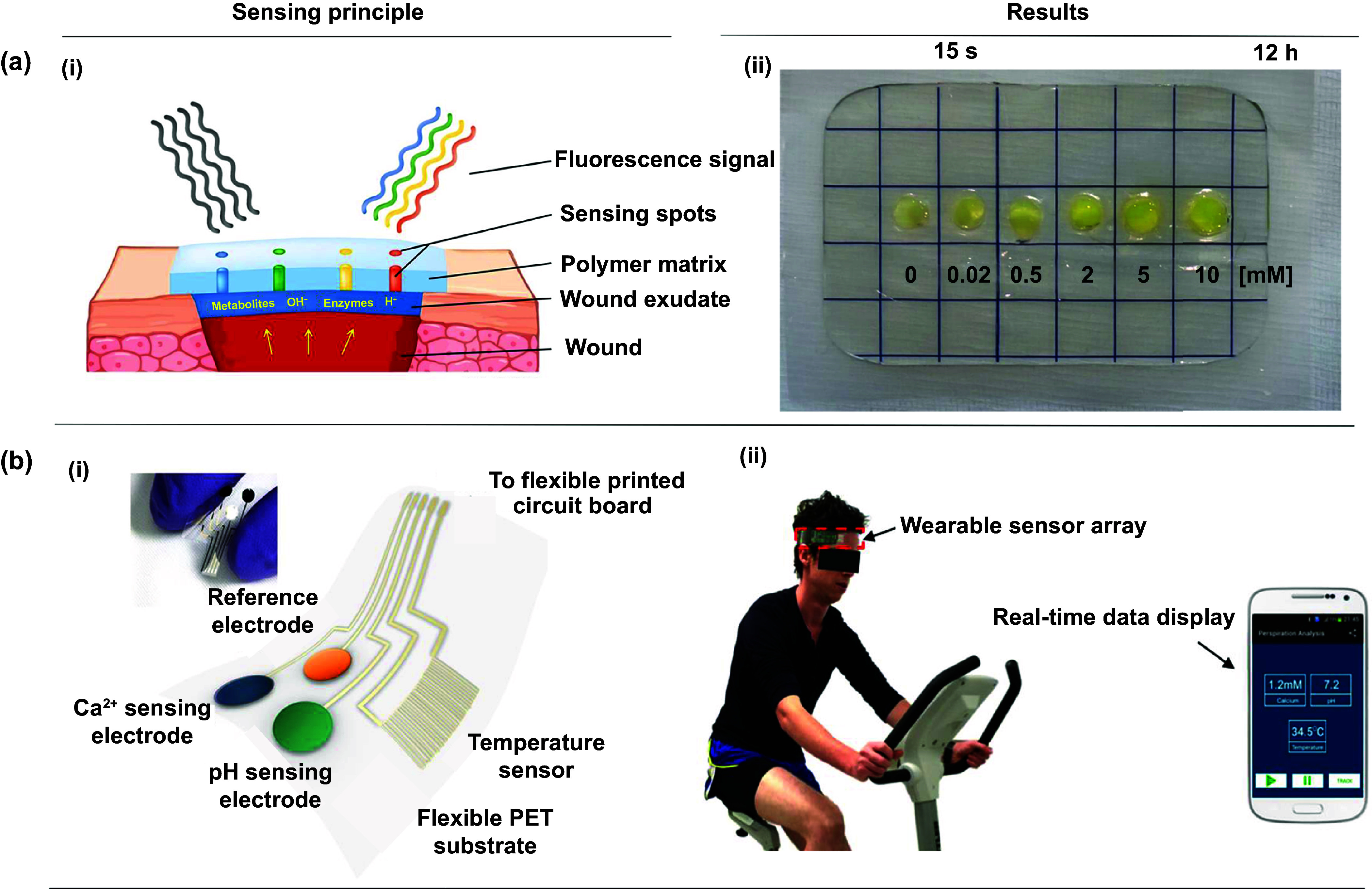
Examples of non-invasive sensing approaches for wound monitoring and wearable sensor arrays (a) Wound monitoring with pH and glucose levels. (i) Diagram illustrating the sensing approach for non-invasive wound monitoring, centered around the measurement of pH values and glucose concentrations, (ii) an image displaying the specialized wound pad designed for analyzing glucose concentrations. Gradations of glucose concentrations range from left to right: 0 mM, 0.02 mM, 0.5 mM, 2 mM, 5 mM, and 10 mM. Reprinted from [382], © 2016 Elsevier B.V. All rights reserved. (b) Flexible sensor array with wearable data capture. (i) Illustration of a flexible sensor array on a flexible polyethylene terephthalat (PET) substrate, incorporating sensors for Ca^2+^, pH, and temperature. The inset displays a photograph of the implemented flexible sensor array, (ii) during stationary cycling, a subject wears a wearable multiplexed sensing system on the forehead. Data are directly read through a customized application and stored on a mobile phone. Reprinted with permission from [385]. Copyright (2016) American Chemical Society.

In recent advancements, Jankowska *et al* presented an innovative wound monitoring technology (figure 5(a)) integrating a pH-sensitive dye and a glucose-detection enzyme system in a hydrogel matrix [382]. This versatile system enables simultaneous qualitative and quantitative assessment of wound status, offering a practical solution for chronic wound management. Moving forward to 2019, Ashley *et al* introduced biosensors for real-time lactate and oxygen monitoring, emphasizing applications in wound monitoring [383]. These compact, soft sensors with lattice-structured and membrane features exhibit robustness in mechanical testing and hold potential for on-skin biofluid monitoring. In 2021, Xia *et al* developed flexible thread-based electrochemical oxygen sensors, providing *in-situ* monitoring of oxygen levels crucial for wound healing [384]. These sensors, with minimal invasiveness and cost-effective fabrication, display high sensitivity and spatial accuracy, making them promising for wound monitoring and organ-on-a-chip studies. Additionally, Nyein *et al* pioneered a wearable platform (figure 5(b)) for simultaneous Ca^2+^ and pH monitoring in body fluids, demonstrating high repeatability [385]. Their flexible sensors allow real-time analysis of sweat, urine, and tears, showcasing practical applications in continuous disease diagnosis and personal health monitoring during activities, such as exercise.

### 4.10. Limitations and critical concerns

Despite significant advancements in 3D bioprinting, researchers still face several challenges that need to be overcome. One major concern is the lack of vascularization and innervation in bioprinted tissues. Without a functional blood vessel network, these constructs cannot effectively absorb nutrients, and their functional expression is limited, hampering their integration within the body [386–388]. Achieving the fabrication of skin constructs that encompass HFs, SGs, and sebaceous glands remains a complex challenge. Additionally, reproducing the natural color and texture of the native skin poses further difficulties. Moreover, the viability and proliferation of cells can be compromised by numerous factors, including the bioprinting method employed, bioink and crosslinking strategies, and types of seeded cells. Another limitation pertains to patient safety. The process of skin bioprinting is still in its initial stages of development, and potential safety concerns may arise when bioprinted skin is directly applied to patients in clinical studies. It is crucial to address these concerns to ensure the safety and effectiveness of the bioprinted skin for clinical applications in the future.

To successfully bioprint skin, a large number of healthy and functional skin cells are required. These cells, including fibroblasts and KCs, are essential components of the skin. However, obtaining enough of these cells for extensive bioprinting still presents a significant logistical challenge. To meet the demands of the bioprinting process, efficient cell sourcing and expansion techniques are required. The distinct biomechanical characteristics of the skin, a highly dynamic and adaptable organ, help to explain its special functions. Both functionally and aesthetically bioprinted skin materials must achieve the ideal balance of elasticity, strength, and flexibility. Research is still being conducted to create biomaterials that accurately mimic these characteristics. While the epidermis can be successfully recreated using contemporary bioprinting techniques, replicating the inner layers, particularly the dermis and subcutaneous tissue, is still a difficult task. The structure, functionality, and overall appearance of the skin are significantly influenced by these inner layers. The microarchitecture of the skin is extremely complex and includes elements like HFs, SGs, and a vast network of blood vessels. This level of detail reproduction in the bioprinted skin is a technical achievement that calls for improvements in accuracy, resolution, and biomaterial compatibility. It is crucial to develop a functional vascular system in the bioprinted skin. This entails precise bioprinting of capillaries, blood vessels, and a vast network that can effectively move immune cells, nutrients, and oxygen throughout the tissue. For bioprinted skin to be long lasting and integrate with the host’s circulatory system, it must successfully vascularize. The tricky inner microarchitecture of the bioprinted skin must be designed and optimized using cutting-edge computational modeling and simulation techniques. These models help scientists better comprehend the complex cellular interactions within the tissue and direct the bioprinting procedure. Modern analytical techniques, such as molecular analysis, histological evaluations, and advanced imaging, are crucial for assessing the performance, safety, and quality of the bioprinted skin. These methods are crucial for quality assurance and confirming the performance and viability of the tissue. Obtaining regulatory approval for bioprinted skin products requires navigating convoluted processes to guarantee that the finished products are effective for the medical or cosmetic uses to which they are to be put. To successfully complete this process, strict safety standards, quality assurance procedures, and ethical considerations must be established.

In the pursuit of seamlessly integrating biosensors with bioprinted smart skin tissues for continuous monitoring, overcoming challenges in material selection and functionality is paramount. Achieving a biosensing platform that requires minimal skin contact and operates continuously demands careful consideration of stability-related features, such as biocompatibility and antifouling capabilities. Hydrophilic polymers are favored for their properties and specific bioreceptor immobilization techniques, like orientated antibodies, enhance sensitivity, crucial for detecting minute bioanalyte changes. Nanomaterials play a pivotal role in improving overall performance. However, developing miniaturized biosensors utilizing micro- and nano-technologies presents challenges in multifunctional integration, while non-invasive skin biofluid collection remains a persistent concern. Electrochemical biosensors offer advantages with simpler circuitry and smaller sample sizes, but optical biosensors face difficulties with external light sources. Skin-mountable biosensors, though promising, encounter issues related to cost and manufacturing time. While certain materials like precious metals contribute to higher costs, projects utilizing cost-effective alternatives, such as carbon-based substrates and graphene sensors, have alleviated this concern.

Safety and potential rejection by the subject’s body are critical considerations for skin-attached or skin-implanted biosensors, particularly with tattoo and patch-based implementations. Concerns regarding patient reactions to temporary tattoo paper or patch adhesives highlight the importance of comfort in data collection. Additionally, addressing data transfer limitations, especially in biosensors lacking wireless capabilities, is crucial for efficient and streamlined data collection [389, 390]. Despite these challenges, various studied projects have reported positive and successful outcomes, showcasing the tremendous potential of biosensors in the future of healthcare. Overcoming these limitations remains a key focus in refining biosensor iterations, recognizing that each type of biosensor comes with its own set of benefits and drawbacks. Selecting the appropriate biosensor type and implementation method is crucial for achieving optimal outcomes in continuous monitoring and personalized healthcare.

### 4.11. Future perspective

As bioprinting becomes widely accessible in the not-too-distant future, the landscape of healthcare is poised for a revolutionary transformation. This innovative technology has the potential to address a wide range of pressing healthcare issues, including the reconstruction of burn wounds, the development of functional organs, and even personalized cancer treatment. Here, we delve more deeply into the plethora of opportunities that bioprinting presents. Burn injuries have long-posed challenging problems for medical professionals. A promising solution is provided by the development of bioprinting, which makes it possible to precisely reconstruct damaged skin that is molded to specific contours of a patient’s wound. This increases functionality while also enhancing the aesthetic results, lowering the possibility of complications and long-term problems. A recurring problem in healthcare has been the lack of organ donors around the world. The possibility of building functional hearts, kidneys, livers, and other essential organs from a patient’s cells becomes a reality with bioprinting. This ground-breaking method lessens the burden of organ scarcity while also saving countless lives. In recent years, groundbreaking advancements in 3D bioprinting and iPSCs have paved the way for revolutionary applications in personalized medicine. The potential to produce organs and tissues from patient-derived cells holds significant implications for delivering customized medications, thereby reducing adverse reactions and complications post-implantation. This technology aims to engineer organs with optimal functionality, mirroring their natural counterparts efficiently.

These collective advancements showcase the evolving landscape of 3D bioprinting and iPSCs in creating sophisticated models for drug screening and disease research, holding promise for the future of personalized medicine and improved patient outcomes. Looking ahead, the future of bioprinting holds immense promise and potential for transforming the landscape of regenerative medicine and personalized healthcare. Continued advancements in technology and research are likely to lead to even more sophisticated and complex 3D bioprinted models that closely mimic the complexity of the native human skin.

## 5. Conclusion

In conclusion, this in-depth review navigates the realm of skin bioprinting marking a pivotal juncture in dermatological research and offering unprecedented opportunities for inventive solutions. The examination extends to the intricacies of pigmentation, HFs, sweat glands, and vascularization, providing a holistic view of the field’s current landscape. As we present the latest advancements in clinical translation, a spotlight is cast on the promising horizons within regenerative medicine, underscoring the potential impact of skin bioprinting on transformative therapeutic applications. Furthermore, the review accentuates the pivotal role of biosensor technologies in augmenting the wound healing process, fostering a deeper understanding of their integration for enhanced clinical outcomes. By critically addressing limitations and concerns, this synthesis serves as a guiding compass for researchers, clinicians, and industry stakeholders, facilitating a nuanced comprehension of the challenges and future trajectories in skin bioprinting, ultimately steering the field towards groundbreaking advancements in regenerative medicine.

## References

[1] Fodor L, Dumitrascu D, Fodor L, Ullmann Y (2019). Skin anatomy. Aesthetic Applications of Intense Pulsed Light.

[2] Weng T T (2021). 3D bioprinting for skin tissue engineering: current status and perspectives. J. Tissue Eng..

[3] Park W, Gao G, Cho DW (2021). Tissue-specific decellularized extracellular matrix bioinks for musculoskeletal tissue regeneration and modeling using 3D bioprinting technology. Int. J. Mol. Sci..

[4] Yan WC, Davoodi P, Vijayavenkataraman S, Tian Y, Ng W C, Fuh J Y H, Robinson K S, Wang CH (2018). 3D bioprinting of skin tissue: from pre-processing to final product evaluation. Adv. Drug Deliv. Rev..

[5] Cai R X, Gimenez-Camino N, Xiao M, Bi S G, Divito K A (2023). Technological advances in three-dimensional skin tissue engineering. Rev. Adv. Mater. Sci..

[6] Kamadjaja D (2017). Tissue engineering in maxillofacial bone reconstruction. J. Stem Cell Res. Tissue Eng..

[7] Zhang Z F, Feng Y H, Wang L, Liu D X, Qin C C, Shi Y B (2022). A review of preparation methods of porous skin tissue engineering scaffolds. Mater. Today Commun..

[8] Fernandes S, Vyas C, Lim P, Pereira R F, Virós A, Bártolo P (2022). 3D bioprinting: an enabling technology to understand melanoma. Cancers.

[9] Ng W L, Qi J T Z, Yeong W Y, Naing M W (2018). Proof-of-concept: 3D bioprinting of pigmented human skin constructs. Biofabrication.

[10] Pourchet L J, Thepot A, Albouy M, Courtial E J, Boher A, Blum L J, Marquette C A (2017). Human skin 3D bioprinting using scaffold-free approach. Adv. Healthcare Mater..

[11] Lee HR, Park J A, Kim S, Jo Y, Kang D, Jung S (2021). 3D microextrusion-inkjet hybrid printing of structured human skin equivalents. Bioprinting.

[12] El-Serafi A T, El-Serafi I T, Elmasry M, Steinvall I, Sjöberg F (2017). Skin regeneration in three dimensions, current status, challenges and opportunities. Differentiation.

[13] Zhang B, Luo Y C, Ma L, Gao L, Li Y T, Xue Q, Yang H Y, Cui Z F (2018). 3D bioprinting: an emerging technology full of opportunities and challenges. Bio-Des. Manuf..

[14] Chameettachal S, Yeleswarapu S, Sasikumar S, Shukla P, Hibare P, Bera A K, Bojedla S S R, Pati F (2019). 3D bioprinting: recent trends and challenges. J. Indian Inst. Sci..

[15] Ozbolat I T (2016). 3D Bioprinting: Fundamentals, Principles and Applications.

[16] Mehrotra P (2016). Biosensors and their applications—a review. J. Oral Biol. Craniofac. Res..

[17] Hasan A, Nurunnabi M, Morshed M, Paul A, Polini A, Kuila T, Al Hariri M, Lee YK, Jaffa A A (2014). Recent advances in application of biosensors in tissue engineering. BioMed Res. Int..

[18] Ramesh M, Janani R, Deepa C, Rajeshkumar L (2023). Nanotechnology-enabled biosensors: a review of fundamentals, design principles, materials, and applications. Biosensors.

[19] Xie Z L, Gao M, Lobo A O, Webster T J (2020). 3D bioprinting in tissue engineering for medical applications: the classic and the hybrid. Polymers.

[20] Shopova D, Yaneva A, Bakova D, Mihaylova A, Kasnakova P, Hristozova M, Sbirkov Y, Sarafian V, Semerdzhieva M (2023). (Bio)printing in personalized medicine—opportunities and potential benefits. Bioengineering.

[21] Kanitakis J (2002). Anatomy, histology and immunohistochemistry of normal human skin. Eur. J. Dermatol..

[22] Halprin K M (1972). Epidermal “turnover time”—a re‐examination. Br. J. Dermatol..

[23] Kumar M S A, Buote N J (2024). The skin. Techniques in Small Animal Wound Management.

[24] Holte K, Biswas A, Herrington C S (2020). The skin. Muir’s Textbook of Pathology.

[25] Chu D H, Wolff K, Goldsmith L A, Katz S I, Gilchrest B A, Paller A S, Leffell D J (2012). Overview of biology, development, and structure of skin. Fitzpatrick’s Dermatology in General Medicine.

[26] Watt F M, Crompton M J, Dexter T M, Wright N A (1998). Epidermal stem cells: markers, patterning and the control of stem cell fate. Phil. Trans. R. Soc. B.

[27] Walko G, Castañón M J, Wiche G (2015). Molecular architecture and function of the hemidesmosome. Cell Tissue Res..

[28] Cichorek M, Wachulska M, Stasiewicz A, Tymińska A (2013). Skin melanocytes: biology and development. Postepy Dermatol. Alergol..

[29] Haass N K, Herlyn M (2005). Normal human melanocyte homeostasis as a paradigm for understanding melanoma. J. Investig. Dermatol. Symp. Proc..

[30] Yousef H, Alhajj M, Sharma S (2020). Anatomy, skin (integument), epidermis. StatPearls.

[31] Hashemi P, Pulitzer M P, Scope A, Kovalyshyn I, Halpern A C, Marghoob A A (2012). Langerhans cells and melanocytes share similar morphologic features under *in vivo* reflectance confocal microscopy: a challenge for melanoma diagnosis. J. Am. Acad. Dermatol..

[32] Bliss D (2005). Layers of the skin. https://visualsonline.cancer.gov/details.cfm?imageid=4362.

[33] Peltonen S, Raiko L, Peltonen J, Mueller E J (2010). Desmosomes in developing human epidermis. Dermatol. Res. Pract..

[34] Roger M (2019). Bioengineering the microanatomy of human skin. J. Anat..

[35] Arda O, Göksügür N, Tüzün Y (2014). Basic histological structure and functions of facial skin. Clin. Dermatol..

[36] Jaitley S, Saraswathi T R (2012). Pathophysiology of Langerhans cells. J. Oral Maxillofac. Pathol..

[37] Breathnach A S (1977). Variations in ultrastructural appearance of Langerhans cells of normal human epidermis. Br. J. Dermatol..

[38] Matoltsy A G (1976). Keratinization. J. Invest. Dermatol..

[39] Kolarsick P A J, Kolarsick M A, Goodwin C (2011). Anatomy and physiology of the skin. J. Dermatol. Nurses’ Assoc..

[40] Nguyen A V, Soulika A M (2019). The dynamics of the skin’s immune system. Int. J. Mol. Sci..

[41] Elias P M (2012). Structure and function of the stratum corneum extracellular matrix. J. Invest. Dermatol..

[42] Nicol N H (2005). Anatomy and physiology of the skin. J. Dermatol. Nurses.

[43] Mauldin E A, Peters-Kennedy J, Maxie M G (2016). Integumentary system. Jubb, Kennedy & Palmer’s Pathology of Domestic Animals: Volume 1.

[44] Amirlak B (2017). Skin anatomy: overview, epidermis, dermis. https://emedicine.medscape.com/article/1294744-overview.

[45] Brown T M, Krishnamurthy K (2019). Histology, dermis. StatPearls.

[46] Haydont V, Bernard B A, Fortunel N O (2019). Age-related evolutions of the dermis: clinical signs, fibroblast and extracellular matrix dynamics. Mech. Ageing Dev..

[47] Marks R, Knight A, Laidler P (1986). Atlas of Skin Pathology.

[48] Monteiro-Riviere N A, Hobson D W (1991). Comparative anatomy, physiology, and biochemistry of mammalian skin. Dermal and Ocular Toxicology.

[49] Rodrigues M, Kosaric N, Bonham C A, Gurtner G C (2019). Wound healing: a cellular perspective. Physiol. Rev..

[50] Darby I A, Laverdet B, Bonté F, Desmoulière A (2014). Fibroblasts and myofibroblasts in wound healing. Clin. Cosmet. Investig. Dermatol..

[51] Tottoli E M, Dorati R, Genta I, Chiesa E, Pisani S, Conti B (2020). Skin wound healing process and new emerging technologies for skin wound care and regeneration. Pharmaceutics.

[52] Hao R N, Cui Z Y, Zhang X D, Tian M, Zhang L Q, Rao F, Xue J J (2022). Rational design and preparation of functional hydrogels for skin wound healing. Front. Chem..

[53] Sorg H, Tilkorn D J, Hager S, Hauser J, Mirastschijski U (2017). Skin wound healing: an update on the current knowledge and concepts. Eur. Surg. Res..

[54] Goldman R (2004). Growth factors and chronic wound healing: past, present, and future. Adv. Skin Wound Care.

[55] Zhao R L, Liang H, Clarke E, Jackson C, Xue M L (2016). Inflammation in chronic wounds. Int. J. Mol. Sci..

[56] Zeng R J, Lin C Q, Lin Z H, Chen H, Lu W Y, Lin C M, Li H H (2018). Approaches to cutaneous wound healing: basics and future directions. Cell Tissue Res..

[57] Eming S A, Martin P, Tomic-Canic M (2014). Wound repair and regeneration: mechanisms, signaling, and translation. Sci. Transl. Med..

[58] Almeida D, Sanjuan-Alberte P, Silva J C, Ferreira F C (2024). 3D (bio)printing of magnetic hydrogels: formulation and applications in tissue engineering. Int. J. Biosci..

[59] Germain N, Dhayer M, Dekiouk S, Marchetti P (2022). Current advances in 3D bioprinting for cancer modeling and personalized medicine. Int. J. Mol. Sci..

[60] Hosseini B S T, Meadows K, Gabriel V, Hu J G, Kim K (2024). Biofabrication of cellulose-based hydrogels for advanced wound healing: a special emphasis on 3D bioprinting. Macromol. Biosci..

[61] Umur E, Bayrak E, Arslan F, Bulut S B, Baysoy E, Kaleli-Can G, Ayan B (2023). Advances in three dimensional bioprinting for wound healing: a comprehensive review. Appl. Sci..

[62] Raffetto J D, Ligi D, Maniscalco R, Khalil R A, Mannello F (2021). Why venous leg ulcers have difficulty healing: overview on pathophysiology, clinical consequences, and treatment. J. Clin. Med..

[63] Bishop E S, Mostafa S, Pakvasa M, Luu H H, Lee M J, Wolf J M, Ameer G A, He TC, Reid R R (2017). 3-D bioprinting technologies in tissue engineering and regenerative medicine: current and future trends. Genes Dis..

[64] Fayyazbakhsh F, Leu M C (2020). A brief review on 3D bioprinted skin substitutes. Proc. Manuf..

[65] Kang M S, Jang J, Jo H J, Kim WH, Kim B, Chun HJ, Lim D, Han DW (2023). Advances and innovations of 3D bioprinting skin. Biomolecules.

[66] Masson J C (1918). Skin grafting. JAMA.

[67] Andreassi A, Bilenchi R, Biagioli M, D’Aniello C (2005). Classification and pathophysiology of skin grafts. Clin. Dermatol..

[68] Valencia I C, Falabella A F, Eaglstein W H (2000). Skin grafting. Dermatol. Clin..

[69] Barrati G E, Koopmann J C F (1984). Skin grafts: physiology and clinical considerations. Otolaryngol. Clin. North Am..

[70] Khan A A, Khan I M, Nguyen P P, Lo E, Chahadeh H, Cerniglia M, Noriega J A (2020). Skin graft techniques. Clin. Podiatr. Med. Surg..

[71] Adams D C, Ramsey M L (2005). Grafts in dermatologic surgery: review and update on full- and split-thickness skin grafts, free cartilage grafts, and composite grafts. Dermatol. Surg..

[72] Kimura N (2002). A microdissected thin tensor fasciae latae perforator flap. Plast. Reconstr. Surg..

[73] Koshima I, Inagawa K, Urushibara K, Moriguchi T (1998). Paraumbilical perforator flap without deep inferior epigastric vessels. Plast. Reconstr. Surg..

[74] Kim E, Drew P J (2022). Management of burn injury. Surgery.

[75] Sánchez P F, Brey E M, Briceño J C (2018). Endothelialization mechanisms in vascular grafts. J. Tissue Eng. Regener. Med..

[76] Bennett L (2007). Elastic Fibre Deposition in Healing Wounds and A Dermal Substitute, and the Consequences for Skin Grafting.

[77] Adigbli G, Alshomer F, Maksimcuka J, Ghali S, Kalaskar D M, Butler P E, Ghali S (2016). Principles of plastic surgery, wound healing, skin grafts and flaps. Textbook of Plastic and Reconstructive Surgery.

[78] Guogienė I, Kievišas M, Grigaitė A, Braziulis K, Rimdeika R (2018). Split-thickness skin grafting: early outcomes of a clinical trial using different graft thickness. J. Wound Care.

[79] Dai C, Shih S, Khachemoune A (2020). Skin substitutes for acute and chronic wound healing: an updated review. J. Dermatol. Treat..

[80] Boyce S T (1996). Cultured skin substitutes: a review. Tissue Eng..

[81] Boyce S T (2001). Design principles for composition and performance of cultured skin substitutes. Burns.

[82] Nyame T T, Chiang H A, Orgill D P (2014). Clinical applications of skin substitutes. Surg. Clin. North Am..

[83] European Commission Ban on animal testing. https://single-market-economy.ec.europa.eu/sectors/cosmetics/ban-animal-testing_en.

[84] Powley T (2015). Procter & Gamble puts skin in 3D bioprinting game. https://www.ft.com/content/02809474-ffe8-11e4-abd5-00144feabdc0.

[85] Sher D (2016). L’Oréal partners with poietis to develop 3D printed hair follicles. https://www.voxelmatters.com/loreal-partners-poietis-develop-3d-printed-hair-follicles/.

[86] L’Oréal Groupe Revolutionizing tissue engineering. https://www.loreal.com/en/news/research-innovation/revolutionizing-tissue-engineering/.

[87] Anon (2023). Chanel and LabSkin create 3D bioprinted skin with pigmentation spots. https://www.premiumbeautynews.com/en/chanel-and-labskin-create-3d,21634.

[88] Madeleine P (2023). Chanel has developed 3D bioprinted skin to improve its skincare projects. https://www.3dnatives.com/en/chanel-has-developed-3d-bioprinted-skin-270220234/.

[89] Mari A (2023). Brazil’s grupo boticário develops 3D skin with bioprinting technology. https://www.forbes.com/sites/angelicamarideoliveira/2023/12/08/brazils-grupo-boticrio-develops-3d-skin-with-bioprinting-technology/.

[90] CTIBiotech 3D bioprinted human skin—the future of cosmetic testing. https://bico.com/blog/3d-bioprinted-human-skin-the-future-of-cosmetic-testing/.

[91] Sher D (2019). BASF and CTIBiotech to Develop First Regenerating 3D Bioprinted Human Skin Model. https://www.basf.com/global/en/media/news-releases/2019/09/p-19-318.

[92] JALA Group (2017). JALA group announces successful printing of Asian skin using 3D bioprinting technology. https://www.prnewswire.com/news-releases/jala-group-announces-successful-printing-of-asian-skin-using-3d-bioprinting-technology-300407828.html.

[93] Listek V (2020). Bioprinted skin patches for diabetic foot ulcers commercialized by rokit healthcare. https://3dprint.com/269464/bioprinted-skin-patches-for-diabetic-foot-ulcers-commercialized-by-rokit-healthcare/.

[94] Lee H J (2019). An interview with Heon Ju Lee on ROCKIT Healthcare’s novel bioprinting treatment for dermal scarring. J. 3D Print. Med..

[95] Hancock A (2023). 3D bioprinting market size, share & trends analysis report by 2030. https://www.linkedin.com/pulse/3d-bioprinting-market-size-share-trends-analysis-report-hancock.

[96] Combellack E, Jessop Z M, Whitaker I S, Thomas D J, Jessop Z M, Whitaker I S (2018). The commercial 3D bioprinting industry. 3D Bioprinting for Reconstructive Surgery: Techniques and Applications.

[97] Kang HW, Lee S J, Ko I K, Kengla C, Yoo J J, Atala A (2016). A 3D bioprinting system to produce human-scale tissue constructs with structural integrity. Nat. Biotechnol..

[98] Ozbolat I T, Hospodiuk M (2016). Current advances and future perspectives in extrusion-based bioprinting. Biomaterials.

[99] Hölzl K, Lin S M, Tytgat L, Van Vlierberghe S, Gu L X, Ovsianikov A (2016). Bioink properties before, during and after 3D bioprinting. Biofabrication.

[100] Derakhshanfar S, Mbeleck R, Xu K G, Zhang X Y, Zhong W, Xing M (2018). 3D bioprinting for biomedical devices and tissue engineering: a review of recent trends and advances. Bioact. Mater..

[101] Cubo N, Garcia M, Del Cañizo J F, Velasco D, Jorcano J L (2017). 3D bioprinting of functional human skin: production and *in vivo* analysis. Biofabrication.

[102] Rimann M, Bono E, Annaheim H, Bleisch M, Graf-Hausner U (2016). Standardized 3D bioprinting of soft tissue models with human primary cells. J. Lab. Autom..

[103] Admane P, Gupta A C, Jois P, Roy S, Lakshmanan C C, Kalsi G, Bandyopadhyay B, Ghosh S (2019). Direct 3D bioprinted full-thickness skin constructs recapitulate regulatory signaling pathways and physiology of human skin. Bioprinting.

[104] Kim B S, Gao G, Kim J Y, Cho DW (2019). 3D cell printing of perfusable vascularized human skin equivalent composed of epidermis, dermis, and hypodermis for better structural recapitulation of native skin. Adv. Healthcare Mater..

[105] Ramesh S, Harrysson O L A, Rao P K, Tamayol A, Cormier D R, Zhang Y B, Rivero I V (2021). Extrusion bioprinting: recent progress, challenges, and future opportunities. Bioprinting.

[106] Chand R, Muhire B S, Vijayavenkataraman S (2022). Computational fluid dynamics assessment of the effect of bioprinting parameters in extrusion bioprinting. Int. J. Bioprinting.

[107] Boularaoui S, Al Hussein G, Khan K A, Christoforou N, Stefanini C (2020). An overview of extrusion-based bioprinting with a focus on induced shear stress and its effect on cell viability. Bioprinting.

[108] Ning L Q, Betancourt N, Schreyer D J, Chen X B (2018). Characterization of cell damage and proliferative ability during and after bioprinting. ACS Biomater. Sci. Eng..

[109] Emmermacher J, Spura D, Cziommer J, Kilian D, Wollborn T, Fritsching U, Steingroewer J, Walther T, Gelinsky M, Lode A (2020). Engineering considerations on extrusion-based bioprinting: interactions of material behavior, mechanical forces and cells in the printing needle. Biofabrication.

[110] Gerdes S, Ramesh S, Mostafavi A, Tamayol A, Rivero I V, Rao P (2021). Extrusion-based 3D (Bio)printed tissue engineering scaffolds: process-structure-quality relationships. ACS Biomater. Sci. Eng..

[111] Chung J H Y, Naficy S, Yue Z L, Kapsa R, Quigley A, Moulton S E, Wallace G G (2013). Bio-ink properties and printability for extrusion printing living cells. Biomater. Sci..

[112] Li Q, Zhang B, Xue Q, Zhao C X, Luo Y C, Zhou H Z, Ma L, Yang H Y, Bai D P (2021). A systematic thermal analysis for accurately predicting the extrusion printability of alginate-gelatin-based hydrogel bioinks. Int. J. Bioprinting.

[113] Ng W L, Wang S, Yeong W Y, Naing M W (2016). Skin bioprinting: impending reality or fantasy?. Trends Biotechnol..

[114] Murry C E, Keller G (2008). Differentiation of embryonic stem cells to clinically relevant populations: lessons from embryonic development. Cell.

[115] Moncal K K (2021). Tissue engineering: intra-operative bioprinting of hard, soft, and hard/soft composite tissues for craniomaxillofacial reconstruction. Adv. Funct. Mater..

[116] Lee W, Lee V, Polio S, Keegan P, Lee JH, Fischer K, Park JK, Yoo SS (2010). On-demand three-dimensional freeform fabrication of multi-layered hydrogel scaffold with fluidic channels. Biotechnol. Bioeng..

[117] Koch L (2012). Skin tissue generation by laser cell printing. Biotechnol. Bioeng..

[118] Shin S, Kwak H, Hyun J (2018). Melanin nanoparticle-incorporated silk fibroin hydrogels for the enhancement of printing resolution in 3D-projection stereolithography of poly(ethylene glycol)-tetraacrylate bio-ink. ACS Appl. Mater. Interfaces.

[119] Kang Y, Yeo M, Derman I D, Ravnic D J, Singh Y P, Alioglu M A, Wu Y, Makkar J, Driskell R R, Ozbolat I T (2024). Intraoperative bioprinting of human adipose-derived stem cells and extra-cellular matrix induces hair follicle-like downgrowths and adipose tissue formation during full-thickness craniomaxillofacial skin reconstruction. Bioact. Mater..

[120] Li X D, Liu B X, Pei B, Chen J W, Zhou D Z, Peng J Y, Zhang X Z, Jia W, Xu T (2020). Inkjet bioprinting of biomaterials. Chem. Rev..

[121] Rayleigh L (1891). Some applications of photography. Nature.

[122] Derby B (2008). Bioprinting: inkjet printing proteins and hybrid cell-containing materials and structures. J. Mater. Chem..

[123] Sweet R G (1965). High frequency recording with electrostatically deflected ink jets. Rev. Sci. Instrum..

[124] Schneider J M, Hendricks C D (1964). Source of uniform-sized liquid droplets. Rev. Sci. Instrum..

[125] Gudapati H, Dey M, Ozbolat I (2016). A comprehensive review on droplet-based bioprinting: past, present and future. Biomaterials.

[126] Derby B (2010). Inkjet printing of functional and structural materials: fluid property requirements, feature stability, and resolution. Annu. Rev. Mater. Res..

[127] Ng W L, Shkolnikov V (2024). Optimizing cell deposition for inkjet-based bioprinting. Int. J. Biosci..

[128] Takagi D, Lin W, Matsumoto T, Yaginuma H, Hemmi N, Hatada S, Seo M (2019). High-precision three-dimensional inkjet technology for live cell bioprinting. Int. J. Bioprinting.

[129] Cui X F, Boland T, D’Lima D D, Lotz M K (2012). Thermal inkjet printing in tissue engineering and regenerative medicin. Recent Pat. Drug Deliv. Formul..

[130] Lee W, Debasitis J C, Lee V K, Lee J H, Fischer K, Edminster K, Park J K, Yoo S S (2009). Multi-layered culture of human skin fibroblasts and keratinocytes through three-dimensional freeform fabrication. Biomaterials.

[131] Skardal A, Mack D, Kapetanovic E, Atala A, Jackson J D, Yoo J, Soker S (2012). Bioprinted amniotic fluid-derived stem cells accelerate healing of large skin wounds. Stem Cells Transl. Med..

[132] Yanez M, Rincon J, Dones A, De Maria C, Gonzales R, Boland T (2015). *In vivo* assessment of printed microvasculature in a bilayer skin graft to treat full-thickness wounds. Tissue Eng. A.

[133] Lee SG, Lee S, Bae HK, Lee K Y, Park C, Kim M S, Lee D H, Chung H M, Kim CY (2024). Evaluation of the therapeutic efficacy of human skin equivalents manufactured through droplet-based bioprinting/nebulization technology. Mol. Cell Toxicol..

[134] Lee V, Singh G, Trasatti J P, Bjornsson C, Xu X W, Tran T N, Yoo SS, Dai G H, Karande P (2014). Design and fabrication of human skin by three-dimensional bioprinting. Tissue Eng. C.

[135] Kim B S, Lee JS, Gao G, Cho DW (2017). Direct 3D cell-printing of human skin with functional transwell system. Biofabrication.

[136] Persaud A, Maus A, Strait L, Zhu D H (2022). 3D bioprinting with live cells. Eng. Regener..

[137] Lee V K, Dai G H (2017). Printing of three-dimensional tissue analogs for regenerative medicine. Ann. Biomed. Eng..

[138] Li M G, Tian X Y, Schreyer D J, Chen X B (2011). Effect of needle geometry on flow rate and cell damage in the dispensing-based biofabrication process. Biotechnol. Prog..

[139] Lee J M, Ng W L, Yeong W Y (2019). Resolution and shape in bioprinting: strategizing towards complex tissue and organ printing. Appl. Phys. Rev..

[140] Blaeser A, Campos D F D, Puster U, Richtering W, Stevens M M, Fischer H (2016). Controlling shear stress in 3D bioprinting is a key factor to balance printing resolution and stem cell integrity. Adv. Healthcare Mater..

[141] Ng W L, Xi H, Shkolnikov V, Goh G L, Suntornnond R, Yeong W Y (2021). Controlling droplet impact velocity and droplet volume: key factors to achieving high cell viability in sub-nanoliter droplet-based bioprinting. Int. J. Bioprinting.

[142] Graham A D, Olof S N, Burke M J, Armstrong J P K, Mikhailova E A, Nicholson J G, Box S J, Szele F G, Perriman A W, Bayley H (2017). High-resolution patterned cellular constructs by droplet-based 3D printing. Sci. Rep..

[143] Ozbolat I T, Ozbolat I T (2017). 5-Droplet-based bioprinting. 3D Bioprinting.

[144] Cheng E, Yu H R, Ahmadi A, Cheung K C (2016). Investigation of the hydrodynamic response of cells in drop on demand piezoelectric inkjet nozzles. Biofabrication.

[145] Tayari K, Chaoui M, Ghariani H, Lahiani M (2014). Influence of the cardiac activity on the surface impedance of a multilayer model.

[146] Ventura R D (2021). An overview of laser-assisted bioprinting (LAB) in tissue engineering applications. Med. Lasers.

[147] Dou C R, Perez V, Qu J, Tsin A, Xu B, Li J Z (2021). A state-of-the-art review of laser-assisted bioprinting and its future research trends. ChemBioEng Rev..

[148] Guillemot F, Souquet A, Catros S, Guillotin B (2010). Laser-assisted cell printing: principle, physical parameters versus cell fate and perspectives in tissue engineering. Nanomedicine.

[149] Kérourédan O, Hakobyan D, Rémy M, Ziane S, Dusserre N, Fricain JC, Delmond S, Thébaud N B, Devillard R (2019). *In situ* prevascularization designed by laser-assisted bioprinting: effect on bone regeneration. Biofabrication.

[150] Odde D J, Renn M J (2000). Laser-guided direct writing of living cells. Biotechnol. Bioeng..

[151] Kawecki F, Clafshenkel W P, Auger F A, Bourget JM, Fradette J, Devillard R (2018). Self-assembled human osseous cell sheets as living biopapers for the laser-assisted bioprinting of human endothelial cells. Biofabrication.

[152] Ozbolat I T (2017). 6-Laser-based bioprinting. 3D Bioprinting.

[153] Catros S (2012). Layer-by-layer tissue microfabrication supports cell proliferation *in vitro* and *in vivo*. Tissue Eng. C.

[154] Koch L (2010). Laser printing of skin cells and human stem cells. Tissue Eng. C.

[155] Gruene M, Pflaum M, Deiwick A, Koch L, Schlie S, Unger C, Wilhelmi M, Haverich A, Chichkov B N (2011). Adipogenic differentiation of laser-printed 3D tissue grafts consisting of human adipose-derived stem cells. Biofabrication.

[156] Michael S, Sorg H, Peck C T, Koch L, Deiwick A, Chichkov B, Vogt P M, Reimers K, Slominski A T (2013). Tissue engineered skin substitutes created by laser-assisted bioprinting form skin-like structures in the dorsal skin fold chamber in mice. PLoS One.

[157] Hopp B, Smausz T, Kresz N, Barna N, Bor Z, Kolozsvári L, Chrisey D B, Szabó A, Nógrádi A (2005). Survival and proliferative ability of various living cell types after laser-induced forward transfer. Tissue Eng..

[158] Barron J A, Krizman D B, Ringeisen B R (2005). Laser printing of single cells: statistical analysis, cell viability, and stress. Ann. Biomed. Eng..

[159] Ringeisen B R, Kim H, Barron J A, Krizman D B, Chrisey D B, Jackman S, Auyeung R Y C, Spargo B J (2004). Laser printing of pluripotent embryonal carcinoma cells. Tissue Eng..

[160] Barron J A, Young H D, Dlott D D, Darfler M M, Krizman D B, Ringeisen B R (2005). Printing of protein microarrays via a capillary-free fluid jetting mechanism. Proteomics.

[161] Zhang Z Y, Xu C X, Xiong R T, Chrisey D B, Huang Y (2017). Effects of living cells on the bioink printability during laser printing. Biomicrofluidics.

[162] Machekposhti S A, Movahed S, Narayan R J (2020). Physicochemical parameters that underlie inkjet printing for medical applications. Biophys. Rev..

[163] Li J P, Chen M J, Fan X Q, Zhou H F (2016). Recent advances in bioprinting techniques: approaches, applications and future prospects. J. Transl. Med..

[164] Vijayavenkataraman S, Lu W F, Fuh J Y H (2016). 3D bioprinting of skin: a state-of-the-art review on modelling, materials, and processes. Biofabrication.

[165] Pitsillides C M, Joe E K, Wei X B, Anderson R R, Lin C P (2003). Selective cell targeting with light-absorbing microparticles and nanoparticles. Biophys. J..

[166] Rider P, Kačarević Ž P, Alkildani S, Retnasingh S, Barbeck M (2018). Bioprinting of tissue engineering scaffolds. J. Tissue Eng..

[167] Murphy S V, Atala A (2014). 3D bioprinting of tissues and organs. Nat. Biotechnol..

[168] Pedde R D (2017). Emerging biofabrication strategies for engineering complex tissue constructs. Adv. Mater..

[169] Wang Z J, Abdulla R, Parker B, Samanipour R, Ghosh S, Kim K (2015). A simple and high-resolution stereolithography-based 3D bioprinting system using visible light crosslinkable bioinks. Biofabrication.

[170] Yeo M, Sarkar A, Singh Y P, Derman I D, Datta P, Ozbolat I T (2024). Synergistic coupling between 3D bioprinting and vascularization strategies. Biofabrication.

[171] Shanjani Y, Pan C C, Elomaa L, Yang Y (2015). A novel bioprinting method and system for forming hybrid tissue engineering constructs. Biofabrication.

[172] Raman R, Bashir R, Atala A, Yoo J J (2015). Stereolithographic 3D bioprinting for biomedical applications. Essentials of 3D Biofabrication and Translation.

[173] He Y, Gu Z M, Xie M J, Fu J Z, Lin H (2020). Why choose 3D bioprinting? Part II: methods and bioprinters. Bio-Des. Manuf..

[174] Hafa L, Breideband L, Posada L R, Torras N, Martinez E, Stelzer E H K, Pampaloni F (2024). Light sheet-based laser patterning bioprinting produces long-term viable full-thickness skin constructs. Adv. Mater..

[175] Fedorovich N E, Oudshoorn M H, van Geemen D, Hennink W E, Alblas J, Dhert W J A (2009). The effect of photopolymerization on stem cells embedded in hydrogels. Biomaterials.

[176] Wendland R J, Conway M T, Worthington K S (2024). Evaluating the polymerization effectiveness and biocompatibility of bio‐sourced, visible light‐based photoinitiator systems. J. Biomed. Mater. Res. A.

[177] Ng W L, Lee J M, Zhou M M, Chen YW, Lee KX A, Yeong W Y, Shen YF (2020). Vat polymerization-based bioprinting—process, materials, applications and regulatory challenges. Biofabrication.

[178] Levato R, Dudaryeva O, Garciamendez-Mijares C E, Kirkpatrick B E, Rizzo R, Schimelman J, Anseth K S, Chen S C, Zenobi-Wong M, Zhang Y S (2023). Light-based vat-polymerization bioprinting. Nat. Rev. Methods Primers.

[179] Bowser D A, Moore M J (2020). Biofabrication of neural microphysiological systems using magnetic spheroid bioprinting. Biofabrication.

[180] Samandari M, Mostafavi A, Quint J, Memić A, Tamayol A (2022). *In situ* bioprinting: intraoperative implementation of regenerative medicine. Trends Biotechnol..

[181] Wu Y, Ravnic D J, Ozbolat I T (2020). Intraoperative bioprinting: repairing tissues and organs in a surgical setting. Trends Biotechnol..

[182] Campbell P G, Weiss L E (2007). Tissue engineering with the aid of inkjet printers. Expert Opin. Biol. Ther..

[183] Binder K W, Zhao W X, Aboushwareb T, Dice D, Atala A, Yoo J J (2010). *In situ* bioprinting of the skin for burns. J. Am. Coll. Surg..

[184] Albanna M (2019). *In situ* bioprinting of autologous skin cells accelerates wound healing of extensive excisional full-thickness wounds. Sci. Rep..

[185] Albouy M (2022). A preliminary study for an intraoperative 3D bioprinting treatment of severe burn injuries. Plast. Reconstr. Surg..

[186] Wang H C, Lian Q, Li D C, Li C H, Zhao T Z, Liang J (2021). Multi-tissue layering and path planning of *in situ* bioprinting for complex skin and soft tissue defects. Rapid Prototyp. J..

[187] Jamróz W, Szafraniec J, Kurek M, Jachowicz R (2018). 3D printing in pharmaceutical and medical applications—recent achievements and challenges. Pharm. Res..

[188] Ajith G, Goyal A S, Rodrigues F C, Thakur G, Pal K, Banerjee I, Sarkar P, Bit A, Kim D, Anis A, Maji S (2021). 12-Natural Polysaccharides for Wound Healing. Food, Medical, and Environmental Applications of Polysaccharides.

[189] Onder O C, Batool S R, Nazeer M A (2022). Self-assembled silk fibroin hydrogels: from preparation to biomedical applications. Mater. Adv..

[190] Hakimi N, Cheng R, Leng L, Sotoudehfar M, Ba P Q, Bakhtyar N, Amini-Nik S, Jeschke M G, Günther A (2018). Handheld skin printer: *in situ* formation of planar biomaterials and tissues. Lab Chip.

[191] Ashammakhi N, Ahadian S, Pountos I, Hu SK, Tellisi N, Bandaru P, Ostrovidov S, Dokmeci M R, Khademhosseini A (2019). *In situ* three-dimensional printing for reparative and regenerative therapy. Biomed. Microdevices.

[192] Singh S, Choudhury D, Yu F, Mironov V, Naing M W (2020). *In situ* bioprinting—bioprinting from benchside to bedside?. Acta Biomater..

[193] Gungor-Ozkerim P S, Inci I, Zhang Y S, Khademhosseini A, Dokmeci M R (2018). Bioinks for 3D bioprinting: an overview. Biomater. Sci..

[194] Yin J, Yan M L, Wang Y C, Fu J Z, Suo H R (2018). 3D bioprinting of low-concentration cell-laden gelatin methacrylate (GelMA) bioinks with a two-step cross-linking strategy. ACS Appl. Mater. Interfaces.

[195] Masri S, Fauzi M B (2021). Current insight of printability quality improvement strategies in natural-based bioinks for skin regeneration and wound healing. Polymers.

[196] Karvinen J, Kellomäki M (2024). 3D-bioprinting of self-healing hydrogels. Eur. Polym. J..

[197] Ramiah P, du Toit L C, Choonara Y E, Kondiah P P D, Pillay V (2020). Hydrogel-based bioinks for 3D bioprinting in tissue regeneration. Front. Mater..

[198] Ullah F (2023). Development of highly-reproducible hydrogel based bioink for regeneration of skin-tissues via 3-D bioprinting technology. Int. J. Biol. Macromol..

[199] He P, Zhao J N, Zhang J M, Li B, Gou Z Y, Gou M L, Li X L (2018). Bioprinting of skin constructs for wound healing. Burns Trauma.

[200] Melchels F P W, Domingos M A N, Klein T J, Malda J, Bartolo P J, Hutmacher D W (2012). Additive manufacturing of tissues and organs. Prog. Polym. Sci..

[201] Gao T, Gillispie G J, Copus J S, Pr A K, Seol YJ, Atala A, Yoo J J, Lee S J (2018). Optimization of gelatin-alginate composite bioink printability using rheological parameters: a systematic approach. Biofabrication.

[202] Gelse K, Pöschl E, Aigner T (2003). Collagens—structure, function, and biosynthesis. Adv. Drug Deliv. Rev..

[203] Burgeson R E, Nimni M E (1992). Collagen types. molecular structure and tissue distribution. Clin. Orthop. Relat. Res..

[204] Chevallay B, Herbage D (2000). Collagen-based biomaterials as 3D scaffold for cell cultures: applications for tissue engineering and gene therapy. Med. Biol. Eng. Comput..

[205] Dong C J, Lv Y G (2016). Application of collagen scaffold in tissue engineering: recent advances and new perspectives. Polymers.

[206] Shpichka A, Butnaru D, Bezrukov E A, Sukhanov R B, Atala A, Burdukovskii V, Zhang Y Y, Timashev P (2019). Skin tissue regeneration for burn injury. Stem Cell Res. Ther..

[207] Mathew-Steiner S S, Roy S, Sen C K (2021). Collagen in wound healing. Bioengineering.

[208] Nocera A D, Comín R, Salvatierra N A, Cid M P (2018). Development of 3D printed fibrillar collagen scaffold for tissue engineering. Biomed. Microdevices.

[209] Yazdanpanah Z, Johnston J D, Cooper D M L, Chen X B (2022). 3D bioprinted scaffolds for bone tissue engineering: state-of-the-art and emerging technologies. Front. Bioeng. Biotechnol..

[210] Adhikari J, Roy A, Das A, Ghosh M, Thomas S, Sinha A, Kim J, Saha P (2021). Effects of processing parameters of 3D bioprinting on the cellular activity of bioinks. Macromol. Biosci..

[211] Stepanovska J, Supova M, Hanzalek K, Broz A, Matejka R (2021). Collagen bioinks for bioprinting: a systematic review of hydrogel properties, bioprinting parameters, protocols, and bioprinted structure characteristics. Biomedicines.

[212] Xu F, Dawson C, Lamb M, Mueller E, Stefanek E, Akbari M, Hoare T (2022). Hydrogels for tissue engineering: addressing key design needs toward clinical translation. Front. Bioeng. Biotechnol..

[213] Tripathi D, Sharma A, Tyagi P, Beniwal C S, Mittal G, Jamini A, Singh H, Tyagi A (2021). Fabrication of three-dimensional bioactive composite scaffolds for hemostasis and wound healing. AAPS PharmSciTech.

[214] Osidak E O (2019). Viscoll collagen solution as a novel bioink for direct 3D bioprinting. J. Mater. Sci., Mater. Med..

[215] Khan M M R, Amin M K, Chakraborty N, Deshmukh K, Pandey M (2023). Advances and prospects of biodegradable polymer nanocomposites for fuel cell applications. Biodegradable and Biocompatible Polymer Nanocomposites: Processing, Characterization, and Applications.

[216] Kokol V, Pottathara Y B, Mihelčič M, Perše L S (2021). Rheological properties of gelatine hydrogels affected by flow- and horizontally-induced cooling rates during 3D cryo-printing. Colloids Surf. A.

[217] Klouda L, Mikos A G (2008). Thermoresponsive hydrogels in biomedical applications. Eur. J. Pharm. Biopharm..

[218] Wang X H, Ao Q, Tian X H, Fan J, Tong H, Hou W J, Bai S L (2017). Gelatin-based hydrogels for organ 3D bioprinting. Polymers.

[219] Dell A C, Wagner G, Own J, Geibel J P (2022). 3D bioprinting using hydrogels: cell inks and tissue engineering applications. Pharmaceutics.

[220] Kačarević Ž P, Rider P M, Alkildani S, Retnasingh S, Smeets R, Jung O, Ivanišević Z, Barbeck M (2018). An introduction to 3D bioprinting: possibilities, challenges and future aspects. Materials.

[221] Li H J, Tan C, Li L (2018). Review of 3D printable hydrogels and constructs. Mater. Des..

[222] Xu L (2023). Bioprinting a skin patch with dual-crosslinked gelatin (GelMA) and silk fibroin (SilMA): an approach to accelerating cutaneous wound healing. Mater. Today Bio.

[223] Ng W L, Yeong W Y, Naing M W (2016). Polyelectrolyte gelatin-chitosan hydrogel optimized for 3D bioprinting in skin tissue engineering. Int. J. Bioprinting.

[224] Piola B, Sabbatini M, Gino S, Invernizzi M, Renò F (2022). 3D bioprinting of gelatin–xanthan gum composite hydrogels for growth of human skin cells. Int. J. Mol. Sci..

[225] Dzobo K, Motaung K S C M, Adesida A (2019). Recent trends in decellularized extracellular matrix bioinks for 3D printing: an updated review. Int. J. Mol. Sci..

[226] Kabirian F, Mozafari M (2020). Decellularized ECM-derived bioinks: prospects for the future. Methods.

[227] Jiang S J, Zhuang Y, Cai M, Wang X D, Lin K L (2023). Decellularized extracellular matrix: a promising strategy for skin repair and regeneration. Eng. Regener..

[228] Di Piazza E, Pandolfi E, Cacciotti I, Del Fattore A, Tozzi A E, Secinaro A, Borro L (2021). Bioprinting technology in skin, heart, pancreas and cartilage tissues: progress and challenges in clinical practice. Int. J. Environ. Res. Public Health.

[229] Kim B S, Das S, Jang J, Cho DW (2020). Decellularized extracellular matrix-based bioinks for engineering tissue- and organ-specific microenvironments. Chem. Rev..

[230] Pati F, Ha DH, Jang J, Han H H, Rhie JW, Cho DW (2015). Biomimetic 3D tissue printing for soft tissue regeneration. Biomaterials.

[231] Choudhury D, Tun H W, Wang T Y, Naing M W (2018). Organ-derived decellularized extracellular matrix: a game changer for bioink manufacturing?. Trends Biotechnol..

[232] Khoshnood N, Zamanian A (2020). Decellularized extracellular matrix bioinks and their application in skin tissue engineering. Bioprinting.

[233] Jorgensen A M, Chou Z S, Gillispie G, Lee S J, Yoo J J, Soker S, Atala A (2020). Decellularized skin extracellular matrix (dsECM) improves the physical and biological properties of fibrinogen hydrogel for skin bioprinting applications. Nanomaterials.

[234] Jang K S (2021). Therapeutic efficacy of artificial skin produced by 3D bioprinting. Materials.

[235] Wang W Q, Meng Q Y, Li Q, Liu J B, Zhou M, Jin Z, Zhao K (2020). Chitosan derivatives and their application in biomedicine. Int. J. Mol. Sci..

[236] Xia Y D, Wang D X, Liu D, Su J Y, Jin Y, Wang D, Han B B, Jiang Z P, Liu B (2022). Applications of chitosan and its derivatives in skin and soft tissue diseases. Front. Bioeng. Biotechnol..

[237] Feng P P, Luo Y, Ke C H, Qiu H F, Wang W, Zhu Y B, Hou R X, Xu L, Wu S Z (2021). Chitosan-based functional materials for skin wound repair: mechanisms and applications. Front. Bioeng. Biotechnol..

[238] Hafezi F, Shorter S, Tabriz A G, Hurt A, Elmes V, Boateng J, Douroumis D (2020). Bioprinting and preliminary testing of highly reproducible novel bioink for potential skin regeneration. Pharmaceutics.

[239] Zhu M, Hu T, Song W, Cui X L, Tian Y, Yao B, Wu M, Huang S, Niu Z W (2023). Guanidinylated/PEGylated chitosan in the bioink promotes the formation of multi-layered keratinocytes in a human skin equivalent. Carbohydr. Polym..

[240] Shaikh F M, Callanan A, Kavanagh E G, Burke P E, Grace P A, McGloughlin T M (2008). Fibrin: a natural biodegradable scaffold in vascular tissue engineering. Cells Tissues Organs.

[241] Kita R, Takahashi A, Kaibara M, Kubota K (2002). Formation of fibrin gel in fibrinogen-thrombin system: static and dynamic light scattering study. Biomacromolecules.

[242] Larsson U (1988). Polymerization and gelation of fibronogen in D_2_O. Eur. J. Biochem..

[243] Mobaraki M, Ghaffari M, Yazdanpanah A, Luo Y Y, Mills D K (2020). Bioinks and bioprinting: a focused review. Bioprinting.

[244] Babu S, Albertino F, Anarkoli A O, De Laporte L (2021). Controlling structure with injectable biomaterials to better mimic tissue heterogeneity and anisotropy. Adv. Healthcare Mater..

[245] Shpichka A (2020). Fibrin-based bioinks: new tricks from an old dog. Int. J. Bioprinting.

[246] Hoppenbrouwers T, Tuk B, Fijneman E M G, de Maat M P M, van Neck J W (2017). Fibrin improves skin wound perfusion in a diabetic rat model. Thromb. Res..

[247] Bacakova M, Musilkova J, Riedel T, Stranska D, Brynda E, Bacakova L, Zaloudkova M (2016). The potential applications of fibrin-coated electrospun polylactide nanofibers in skin tissue engineering. Int. J. Nanomed..

[248] Horch R E, Bannasch H, Stark G B (2001). Transplantation of cultured autologous keratinocytes in fibrin sealant biomatrix to resurface chronic wounds. Transplant. Proc..

[249] Cavallo A, Al Kayal T, Mero A, Mezzetta A, Guazzelli L, Soldani G, Losi P (2023). Fibrinogen-based bioink for application in skin equivalent 3D bioprinting. J. Funct. Biomater..

[250] Mazlyzam A L, Aminuddin B S, Fuzina N H, Norhayati M M, Fauziah O, Isa M R, Saim L, Ruszymah B H I (2007). Reconstruction of living bilayer human skin equivalent utilizing human fibrin as a scaffold. Burns.

[251] Petta D, D’Amora U, Ambrosio L, Grijpma D W, Eglin D, D’Este M (2020). Hyaluronic acid as a bioink for extrusion-based 3D printing. Biofabrication.

[252] Gopinathan J, Noh I (2018). Recent trends in bioinks for 3D printing. Biomater. Res..

[253] Si H P, Xing T L, Ding Y L, Zhang H B, Yin R X, Zhang W J (2019). 3D bioprinting of the sustained drug release wound dressing with double-crosslinked hyaluronic-acid-based hydrogels. Polymers.

[254] Bavaresco B, Comín R, Salvatierra N A, Cid M P (2020). Three-dimensional printing of collagen and hyaluronic acid scaffolds with dehydrothermal treatment crosslinking. Compos. Commun..

[255] Zhou Y, Fan Y C, Chen Z, Yue Z L, Wallace G (2022). Catechol functionalized ink system and thrombin-free fibrin gel for fabricating cellular constructs with mechanical support and inner micro channels. Biofabrication.

[256] Thakur B R, Singh R K, Handa A K, Rao M A (1997). Chemistry and uses of pectin—a review. Crit. Rev. Food Sci. Nutr..

[257] Suntornnond R, An J, Chua C K (2017). Bioprinting of thermoresponsive hydrogels for next generation tissue engineering: a review. Macromol. Mater. Eng..

[258] Pereira R F, Sousa A, Barrias C C, Bártolo P J, Granja P L (2018). A single-component hydrogel bioink for bioprinting of bioengineered 3D constructs for dermal tissue engineering. Mater. Horiz..

[259] Ridley B L, O’Neill M A, Mohnen D (2001). Pectins: structure, biosynthesis, and oligogalacturonide-related signaling. Phytochemistry.

[260] Pereira R F, Barrias C C, Bártolo P J, Granja P L (2018). Cell-instructive pectin hydrogels crosslinked via thiol-norbornene photo-click chemistry for skin tissue engineering. Acta Biomater..

[261] Jáuregui K M G, Cabrera J C C, Ceniceros E P S, Hernández J L M, Ilyina A (2009). A new formulated stable papin-pectin aerosol spray for skin wound healing. Biotechnol. Bioprocess Eng..

[262] Türkkan S, Atila D, Akdağ A, Tezcaner A (2018). Fabrication of functionalized citrus pectin/silk fibroin scaffolds for skin tissue engineering. J. Biomed. Mater. Res. B.

[263] Hospodiuk M, Dey M, Sosnoski D, Ozbolat I T (2017). The bioink: a comprehensive review on bioprintable materials. Biotechnol. Adv..

[264] Lee K Y, Mooney D J (2012). Alginate: properties and biomedical applications. Prog. Polym. Sci..

[265] Ramakrishnan R, Kasoju N, Raju R, Geevarghese R, Gauthaman A, Bhatt A (2022). Exploring the potential of alginate-gelatin-diethylaminoethyl cellulose-fibrinogen based bioink for 3D bioprinting of skin tissue constructs. Carbohydr. Polym. Technol. Appl..

[266] Rezvanian M, Amin M C I M, Ng SF (2016). Development and physicochemical characterization of alginate composite film loaded with simvastatin as a potential wound dressing. Carbohydrate Polym..

[267] Cheng L H H (2019). Properties of an alginate-gelatin-based bioink and its potential impact on cell migration, proliferation, and differentiation. Int. J. Biol. Macromol..

[268] Hashimoto T, Suzuki Y, Tanihara M, Kakimaru Y, Suzuki K (2004). Development of alginate wound dressings linked with hybrid peptides derived from laminin and elastin. Biomaterials.

[269] Millar S E (2002). Molecular mechanisms regulating hair follicle development. J. Invest. Dermatol..

[270] Cotsarelis G (2006). Epithelial stem cells: a folliculocentric view. J. Invest. Dermatol..

[271] Madaan A, Verma R, Singh A T, Jaggi M (2018). Review of hair follicle dermal papilla cells as *in vitro* screening model for hair growth. Int. J. Cosmet. Sci..

[272] Delevoye C (2014). Melanin transfer: the keratinocytes are more than gluttons. J. Invest. Dermatol..

[273] Swope V B, Supp A P, Boyce S T (2002). Regulation of cutaneous pigmentation by titration of human melanocytes in cultured skin substitutes grafted to athymic mice. Wound Repair. Regener..

[274] Lee A Y, Kim J Y, Park C D, Lee J H, Lee C H, Do A Y (2012). Co-culture of melanocytes with adipose-derived stem cells as a potential substitute for co-culture with keratinocytes. Acta Derm. Venereol..

[275] Has C, Nyström A (2015). Epidermal basement membrane in health and disease. Curr. Top. Membr..

[276] Amano S (2016). Characterization and mechanisms of photoageing-related changes in skin. Damages of basement membrane and dermal structures. Exp. Dermatol..

[277] Weinberg W C, Goodman L V, George C, Morgan D L, Ledbetter S, Yuspa S H, Lichti U (1993). Reconstitution of hair follicle development *in vivo*: determination of follicle formation, hair growth, and hair quality by dermal cells. J. Invest. Dermatol..

[278] Lee J, Böscke R, Tang PC, Hartman B H, Heller S, Koehler K R (2018). Hair follicle development in mouse pluripotent stem cell-derived skin organoids. Cell Rep..

[279] Jeong S, Na Y, Nam HM, Sung G Y (2023). Skin-on-a-chip strategies for human hair follicle regeneration. Exp. Dermatol..

[280] Aoki H, Hara A, Motohashi T, Osawa M, Kunisada T (2011). Functionally distinct melanocyte populations revealed by reconstitution of hair follicles in mice. Pigm. Cell Melanoma Res..

[281] Xiao SE, Miao Y, Wang J, Jiang W, Fan ZX, Liu XM, Hu ZQ (2017). As a carrier-transporter for hair follicle reconstitution, platelet-rich plasma promotes proliferation and induction of mouse dermal papilla cells. Sci. Rep..

[282] Liang Y H, Silva K A, Kennedy V, Sundberg J P (2011). Comparisons of mouse models for hair follicle reconstitution. Exp. Dermatol..

[283] Abaci H E, Coffman A, Doucet Y, Chen J, Jacków J, Wang E, Guo Z Y, Shin J U, Jahoda C A, Christiano A M (2018). Tissue engineering of human hair follicles using a biomimetic developmental approach. Nat. Commun..

[284] Zhao W X, Chen H Y, Zhang Y, Zhou D Z, Liang L, Liu B X, Xu T (2022). Adaptive multi-degree-of-freedom *in situ* bioprinting robot for hair-follicle-inclusive skin repair: a preliminary study conducted in mice. Bioeng. Transl. Med..

[285] Chen H Y, Zhang Y, Zhou D Z, Ma X X, Yang S M, Xu T (2022). Mechanical engineering of hair follicle regeneration by *in situ* bioprinting. Biomater. Adv..

[286] Nanmo A, Yan L, Asaba T, Wan L C, Kageyama T, Fukuda J (2023). Bioprinting of hair follicle germs for hair regenerative medicine. Acta Biomater..

[287] Kang D N (2023). 3D bioprinting of a gelatin-alginate hydrogel for tissue-engineered hair follicle regeneration. Acta Biomater..

[288] Catarino C M, Schuck D C, Dechiario L, Karande P (2023). Incorporation of hair follicles in 3D bioprinted models of human skin. Sci. Adv..

[289] Huang S, Wiszniewski L, Constant S, Kapetanović I M (2011). The use of *in vitro* 3D cell models in drug development for respiratory diseases. Drug Discovery and Development—Present and Future.

[290] Liu N B, Huang S, Yao B, Xie J F, Wu X, Fu X B (2016). 3D bioprinting matrices with controlled pore structure and release function guide *in vitro* self-organization of sweat gland. Sci. Rep..

[291] Wang R (2019). Redirecting differentiation of mammary progenitor cells by 3D bioprinted sweat gland microenvironment. Burns Trauma.

[292] Zhang Y J (2021). Using bioprinting and spheroid culture to create a skin model with sweat glands and hair follicles. Burns Trauma.

[293] Tarassoli S P, Jessop Z M, Al-Sabah A, Gao N, Whitaker S, Doak S, Whitaker I S (2018). Skin tissue engineering using 3D bioprinting: an evolving research field. J. Plast. Reconstr. Aesthet. Surg..

[294] Velasquillo C, Galue E A, Rodriquez L, Ibarra C, Ibarra-Ibarra L G (2013). Skin 3D bioprinting. applications in cosmetology. J. Cosmet. Dermatol. Sci. Appl..

[295] Olejnik A, Semba J A, Kulpa A, Dańczak-Pazdrowska A, Rybka J D, Gornowicz-Porowska J (2022). 3D bioprinting in skin related research: recent achievements and application perspectives. ACS Synth. Biol..

[296] Li L, Fukunaga-Kalabis M, Yu H, Xu X W, Kong J, Lee J T, Herlyn M (2010). Human dermal stem cells differentiate into functional epidermal melanocytes. J. Cell Sci..

[297] Li L, Fukunaga-Kalabis M, Herlyn M (2011). The three-dimensional human skin reconstruct model: a tool to study normal skin and melanoma progression. J. Vis. Exp..

[298] Duval C, Chagnoleau C, Pouradier F, Sextius P, Condom E, Bernerd F (2012). Human skin model containing melanocytes: essential role of keratinocyte growth factor for constitutive pigmentation-functional response to α-melanocyte stimulating hormone and forskolin. Tissue Eng. C.

[299] Dinella J, Koster M I, Koch P J (2014). Use of induced pluripotent stem cells in dermatological research. J. Invest. Dermatol..

[300] Bilousova G, Roop D R (2014). Induced pluripotent stem cells in dermatology: potentials, advances, and limitations. Cold Spring Harb. Perspect. Med..

[301] Itoh M, Kiuru M, Cairo M S, Christiano A M (2011). Generation of keratinocytes from normal and recessive dystrophic epidermolysis bullosa-induced pluripotent stem cells. Proc. Natl Acad. Sci. USA.

[302] Itoh M, Umegaki-Arao N, Guo Z Y, Liu L, Higgins C A, Christiano A M, Eckert R (2013). Generation of 3D skin equivalents fully reconstituted from human induced pluripotent stem cells (iPSCs). PLoS One.

[303] Muller Q, Beaudet MJ, De Serres-bérard T, Bellenfant S, Flacher V, Berthod F (2018). Development of an innervated tissue-engineered skin with human sensory neurons and Schwann cells differentiated from iPS cells. Acta Biomater..

[304] Guo Z Y (2021). Engineering human skin model innervated with itch sensory neuron-like cells differentiated from induced pluripotent stem cells. Bioeng. Transl. Med..

[305] Pappalardo A, Herron L, Cespedes D E A, Abaci H E (2021). Quantitative evaluation of human umbilical vein and induced pluripotent stem cell-derived endothelial cells as an alternative cell source to skin-specific endothelial cells in engineered skin grafts. Adv. Wound Care.

[306] Bezenah J R, Rioja A Y, Juliar B, Friend N, Putnam A J (2019). Assessing the ability of human endothelial cells derived from induced-pluripotent stem cells to form functional microvasculature *in vivo*. Biotechnol. Bioeng..

[307] Abaci H E, Guo Z Y, Coffman A, Gillette B, Lee WH, Sia S K, Christiano A M (2016). Human skin constructs with spatially controlled vasculature using primary and iPSC-derived endothelial cells. Adv. Healthcare Mater..

[308] Hafner AL (2016). Brown-like adipose progenitors derived from human induced pluripotent stem cells: identification of critical pathways governing their adipogenic capacity. Sci. Rep..

[309] Ahfeldt T (2012). Programming human pluripotent stem cells into white and brown adipocytes. Nat. Cell Biol..

[310] Bernareggi D, Pouyanfard S, Kaufman D S (2019). Development of innate immune cells from human pluripotent stem cells. Exp. Hematol..

[311] Nianias A, Themeli M (2019). Induced pluripotent stem cell (iPSC)–derived lymphocytes for adoptive cell immunotherapy: recent advances and challenges. Curr. Hematol. Malig. Rep..

[312] Jacków J (2019). CRISPR/Cas9-based targeted genome editing for correction of recessive dystrophic epidermolysis bullosa using iPS cells. Proc. Natl Acad. Sci. USA.

[313] Lee J, van der Valk W H, Serdy S A, Deakin C C, Kim J, Le A P, Koehler K R (2022). Generation and characterization of hair-bearing skin organoids from human pluripotent stem cells. Nat. Protocols.

[314] Hong ZX, Zhu ST, Li H, Luo JZ, Yang Y, An Y, Wang X, Wang K (2023). Bioengineered skin organoids: from development to applications. Mil. Med. Res..

[315] Nguyen D (2017). Cartilage tissue engineering by the 3D bioprinting of iPS cells in a nanocellulose/alginate bioink. Sci. Rep..

[316] Koch L, Deiwick A, Franke A, Schwanke K, Haverich A, Zweigerdt R, Chichkov B (2018). Laser bioprinting of human induced pluripotent stem cells—the effect of printing and biomaterials on cell survival, pluripotency, and differentiation. Biofabrication.

[317] Gu Q, Tomaskovic‐Crook E, Wallace G G, Crook J M (2017). 3D bioprinting human induced pluripotent stem cell constructs for *in situ* cell proliferation and successive multilineage differentiation. Adv. Healthcare Mater..

[318] Ji W Q, Hou B, Lin W G, Wang L L, Zheng W H, Li W D, Zheng J, Wen X J, He P (2020). 3D bioprinting a human iPSC-derived MSC-loaded scaffold for repair of the uterine endometrium. Acta Biomater..

[319] Lin W M, Chen M, Hu C, Qin S Y, Chu C Y, Xiang L, Man Y, Qu Y L (2018). Endowing iPSC-derived MSCs with angiogenic and keratinogenic differentiation potential: a promising cell source for skin tissue engineering. BioMed Res. Int..

[320] Arai K, Murata D, Verissimo A R, Mukae Y, Itoh M, Nakamura A, Morita S, Nakayama K, Matsusaki M (2018). Fabrication of scaffold-free tubular cardiac constructs using a Bio-3D printer. PLoS One.

[321] Ma X Y (2016). Deterministically patterned biomimetic human iPSC-derived hepatic model via rapid 3D bioprinting. Proc. Natl Acad. Sci. USA.

[322] Soman S S, Vijayavenkataraman S (2020). Applications of 3D bioprinted-induced pluripotent stem cells in healthcare. Int. J. Bioprinting.

[323] Shahin H, Elmasry M, Steinvall I, Söberg F, El-Serafi A (2020). Vascularization is the next challenge for skin tissue engineering as a solution for burn management. Burns Trauma.

[324] Cui H T, Nowicki M, Fisher J P, Zhang L G (2017). 3D bioprinting for organ regeneration. Adv. Healthcare Mater..

[325] Chen E P, Toksoy Z, Davis B A, Geibel J P (2021). 3D bioprinting of vascularized tissues for *in vitro* and *in vivo* applications. Front. Bioeng. Biotechnol..

[326] Hauser P V, Chang HM, Nishikawa M, Kimura H, Yanagawa N, Hamon M (2021). Bioprinting scaffolds for vascular tissues and tissue vascularization. Bioengineering.

[327] Tripathi S, Mandal S S, Bauri S, Maiti P (2023). 3D bioprinting and its innovative approach for biomedical applications. MedComm.

[328] Joshi A, Choudhury S, Gugulothu S B, Visweswariah S S, Chatterjee K (2022). Strategies to promote vascularization in 3D printed tissue scaffolds: trends and challenges. Biomacromolecules.

[329] Später T, Ampofo E, Menger M D, Laschke M W (2020). Combining vascularization strategies in tissue engineering: the faster road to success?. Front. Bioeng. Biotechnol..

[330] Frueh F S, Menger M D, Lindenblatt N, Giovanoli P, Laschke M W (2017). Current and emerging vascularization strategies in skin tissue engineering. Crit. Rev. Biotechnol..

[331] Kim B S, Ahn M, Cho WW, Gao G, Jang J, Cho DW (2021). Engineering of diseased human skin equivalent using 3D cell printing for representing pathophysiological hallmarks of type 2 diabetes *in vitro*. Biomaterials.

[332] Dai LG, Dai NT, Chen TY, Kang LY, Hsu SH (2022). A bioprinted vascularized skin substitute with fibroblasts, keratinocytes, and endothelial progenitor cells for skin wound healing. Bioprinting.

[333] Kim B S, Kwon Y W, Kong JS, Park G T, Gao G, Han W, Kim MB, Lee H, Kim J H, Cho DW (2018). 3D cell printing of *in vitro* stabilized skin model and *in vivo* pre-vascularized skin patch using tissue-specific extracellular matrix bioink: a step towards advanced skin tissue engineering. Biomaterials.

[334] Rimal R (2021). Dynamic flow enables long-term maintenance of 3-D vascularized human skin models. Appl. Mater. Today.

[335] Baltazar T, Jiang B, Moncayo A, Merola J, Albanna M Z, Saltzman W M, Pober J S (2022). 3D bioprinting of an implantable xeno‐free vascularized human skin graft. Bioeng. Transl. Med..

[336] Oliveira H, Médina C, Labrunie G, Dusserre N, Catros S, Magnan L, Handschin C, Stachowicz M L, Fricain JC, L’Heureux N (2022). Cell-assembled extracellular matrix (CAM): a human biopaper for the biofabrication of pre-vascularized tissues able to connect to the host circulation *in vivo*. Biofabrication.

[337] Karande P, Baltazar T, Merola J, Catarino C (2020). Breaking barriers—printing vascularized skin.

[338] Phua Q H, Han H A, Soh BS (2021). Translational stem cell therapy: vascularized skin grafts in skin repair and regeneration. J. Transl. Med..

[339] Yang R H, Yang S, Zhao J L, Hu X M, Chen X D, Wang J R, Xie J L, Xiong K (2020). Progress in studies of epidermal stem cells and their application in skin tissue engineering. Stem Cell Res. Ther..

[340] Wang R, Wang Y H, Yao B, Hu T, Li Z, Huang S, Fu X B (2019). Beyond 2D: 3D bioprinting for skin regeneration. Int. Wound J..

[341] Oualla-Bachiri W, Fernández-González A, Quiñones-Vico M I, Arias-Santiago S (2020). From grafts to human bioengineered vascularized skin substitutes. Int. J. Mol. Sci..

[342] Pirayesh A, Hoeksema H, Richters C, Verbelen J, Monstrey S (2015). Glyaderm^®^ dermal substitute: clinical application and long-term results in 55 patients. Burns.

[343] Shahrokhi S, Arno A, Jeschke M G (2014). The use of dermal substitutes in burn surgery: acute phase. Wound Repair. Regener..

[344] Ho J K, Shao H W, You C G, Pan X L, Wang X G, Chen G X, Khan A, Han C M (2019). Successful application of tissue engineering skin to third degree burn wound on lateral thorax: a case study. Biomed. J. Sci. Tech. Res..

[345] Demircan M, Cicek T, Yetis M I (2015). Preliminary results in single-step wound closure procedure of full-thickness facial burns in children by using the collagen-elastin matrix and review of pediatric facial burns. Burns.

[346] Liu J, Zhou Z T, Zhang M, Song F, Feng C, Liu H C (2022). Simple and robust 3D bioprinting of full-thickness human skin tissue. Bioengineered.

[347] Baltazar T (2020). Three dimensional bioprinting of a vascularized and perfusable skin graft using human keratinocytes, fibroblasts, pericytes, and endothelial cells. Tissue Eng. A.

[348] Gore D C (1997). Outcome and cost analysis for outpatient skin grafting. J. Trauma.

[349] Pearce F B, Richardson K A (2017). Negative pressure wound therapy, staged excision and definitive closure with split-thickness skin graft for axillary hidradenitis suppurativa: a retrospective study. J. Wound Care.

[350] Millás A, Lago J, Vasquez-Pinto L, Massaguer P, Maria-Engler S S (2019). Approaches to the development of 3D bioprinted skin models: the case of natura cosmetics. Int. J. Adv. Med. Biotechnol..

[351] Sarkiri M, Fox S C, Fratila-Apachitei L E, Zadpoor A A (2019). Bioengineered skin intended for skin disease modeling. Int. J. Mol. Sci..

[352] Vijayavenkataraman S (2017). 3D bioprinted skin: the first ‘to-be’ successful printed organ?. J. 3D Print. Med..

[353] Sekar M P, Budharaju H, Zennifer A, Sethuraman S, Vermeulen N, Sundaramurthi D, Kalaskar D M (2021). Current standards and ethical landscape of engineered tissues—3D bioprinting perspective. J. Tissue Eng..

[354] Ramadan Q, Zourob M (2021). 3D bioprinting at the frontier of regenerative medicine, pharmaceutical, and food industries. Front. Med. Technol..

[355] Jovic T H, Combellack E J, Jessop Z M, Whitaker I S (2020). 3D bioprinting and the future of surgery. Front. Surg..

[356] Varkey M, Atala A (2015). Organ bioprinting: a closer look at ethics and policies. Wake Forest J. Law Policy.

[357] FDA (2016). Technical considerations for additive manufactured devices draft guidance for industry and food and drug administration staff. https://www.fda.gov/regulatory-information/search-fda-guidance-documents/technical-considerations-additive-manufactured-medical-devices.

[358] Mullins E (2022). Scientific opinion on development needs for the allergenicity and protein safety assessment of food and feed products derived from biotechnology. EFSA J..

[359] Lorenz A, Raven M, Blind K (2019). The role of standardization at the interface of product and process development in biotechnology. J. Technol. Transfer.

[360] Fogel D B (2018). Factors associated with clinical trials that fail and opportunities for improving the likelihood of success: a review. Contemp. Clin. Trials Commun..

[361] Liu X, Michael S, Bharti K, Ferrer M, Song M J (2020). A biofabricated vascularized skin model of atopic dermatitis for preclinical studies. Biofabrication.

[362] Lègues M, Milet C, Forraz N, Berthelemy N, Pain S, André-Frei V, Cadau S, Mcguckin C (2020). The world’s first 3D bioprinted immune skin model suitable for screening drugs and ingredients for normal and inflamed skin.

[363] de Andrés J L, Ruiz-Toranzo M, Antich C, Chocarro-Wrona C, López-Ruíz E, Jiménez G, Marchal J A (2023). Biofabrication of a tri-layered 3D-bioprinted CSC-based malignant melanoma model for personalized cancer treatment. Biofabrication.

[364] Browning J R (2020). A 3D biofabricated cutaneous squamous cell carcinoma tissue model with multi-channel confocal microscopy imaging biomarkers to quantify antitumor effects of chemotherapeutics in tissue. Oncotarget.

[365] Duan J H, Cao Y Y, Shen Z Z, Cheng Y Q, Ma Z W, Wang L J, Zhang Y T, An Y C, Sang S B (2022). 3D bioprinted GelMA/PEGDA hybrid scaffold for establishing an *in vitro* model of melanoma. J. Microbiol. Biotechnol..

[366] Larson P J, Chong D, Fleming E, Oh J (2021). Challenges in developing a human model system for skin microbiome research. J. Invest. Dermatol..

[367] Yin Z, Guo H, Li Y X, Chiu J, Tian L M (2020). Ultrastable plasmonic bioink for printable point-of-care biosensors. ACS Appl. Mater. Interfaces.

[368] Mohanty S P, Kougianos E (2006). Biosensors: a tutorial review. IEEE Potentials.

[369] Singh A K, Mittal S, Das M, Saharia A, Tiwari M (2023). Optical biosensors: a decade in review. Alex. Eng. J..

[370] Yuqing M, Jianguo J G, Jianrong C (2003). Ion sensitive field effect transducer-based biosensors. Biotechnol. Adv..

[371] Youssef K, Ullah A, Rezai P, Hasan A, Amirfazli A (2023). Recent advances in biosensors for real time monitoring of pH, temperature, and oxygen in chronic wounds. Mater. Today Bio.

[372] Kim J, Campbell A S, de Ávila B E F, Wang J (2019). Wearable biosensors for healthcare monitoring. Nat. Biotechnol..

[373] Salvo P, Dini V, Kirchhain A, Janowska A, Oranges T, Chiricozzi A, Lomonaco T, Di Francesco F, Romanelli M (2017). Sensors and biosensors for C-reactive protein, temperature and pH, and their applications for monitoring wound healing: a review. Sensors.

[374] Starly B, Choubey A (2008). Enabling sensor technologies for the quantitative evaluation of engineered tissue. Ann. Biomed. Eng..

[375] Goode J A, Rushworth J V H, Millner P A (2015). Biosensor regeneration: a review of common techniques and outcomes. Langmuir.

[376] Van De Ven M (2014). Chronic wound healing and woundbed-biofilm interactions in silico. Biophys. J..

[377] Goto T, Saligan L N (2020). Wound pain and wound healing biomarkers from wound exudate: a scoping review. J. Wound Ostomy Continence Nurs..

[378] Gao Y J (2021). A flexible multiplexed immunosensor for point-of-care *in situ* wound monitoring. Sci. Adv..

[379] Mertz P M, Ovington L G (1993). Wound healing microbiology. Dermatol. Clin..

[380] Jones E M, Cochrane C A, Percival S L (2015). The effect of pH on the extracellular matrix and biofilms. Adv. Wound Care.

[381] Dargaville T R, Farrugia B L, Broadbent J A, Pace S, Upton Z, Voelcker N H (2013). Sensors and imaging for wound healing: a review. Biosens. Bioelectron..

[382] Jankowska D A, Bannwarth M B, Schulenburg C, Faccio G, Maniura-Weber K, Rossi R M, Scherer L, Richter M, Boesel L F (2017). Simultaneous detection of pH value and glucose concentrations for wound monitoring applications. Biosens. Bioelectron..

[383] Ashley B K, Brown M S, Park Y, Kuan S, Koh A (2019). Skin-inspired, open mesh electrochemical sensors for lactate and oxygen monitoring. Biosens. Bioelectron..

[384] Xia J F, Sonkusale S (2021). Flexible thread-based electrochemical sensors for oxygen monitoring. Analyst.

[385] Nyein H Y Y (2016). A wearable electrochemical platform for noninvasive simultaneous monitoring of Ca^2+^ and pH. ACS Nano.

[386] Nothdurfter D, Ploner C, Coraça-Huber D C, Wilflingseder D, Müller T, Hermann M, Hagenbuchner J, Ausserlechner M J (2022). 3D bioprinted, vascularized neuroblastoma tumor environment in fluidic chip devices for precision medicine drug testing. Biofabrication.

[387] Patra S, Young V (2016). A review of 3D printing techniques and the future in biofabrication of bioprinted tissue. Cell Biochem. Biophys..

[388] Hendrickx B, Vranckx J J, Luttun A (2011). Cell-based vascularization strategies for skin tissue engineering. Tissue Eng. B.

[389] Haleem A, Javaid M, Singh R P, Suman R, Rab S (2021). Biosensors applications in medical field: a brief review. Sens. Int..

[390] Bhatia D, Paul S, Acharjee T, Ramachairy S S (2024). Biosensors and their widespread impact on human health. Sens. Int..

